# Tissue-specific mRNA expression profiling in grape berry tissues

**DOI:** 10.1186/1471-2164-8-187

**Published:** 2007-06-21

**Authors:** Jerome Grimplet, Laurent G Deluc, Richard L Tillett, Matthew D Wheatley, Karen A Schlauch, Grant R Cramer, John C Cushman

**Affiliations:** 1 Department of Biochemistry and Molecular Biology, Mail Stop 200, University of Nevada, Reno, NV 89557, USA; 2 Department of Genetics and Genomics, Boston University School of Medicine, Boston, MA, 02118, USA

## Abstract

**Background:**

Berries of grape (*Vitis vinifera*) contain three major tissue types (skin, pulp and seed) all of which contribute to the aroma, color, and flavor characters of wine. The pericarp, which is composed of the exocarp (skin) and mesocarp (pulp), not only functions to protect and feed the developing seed, but also to assist in the dispersal of the mature seed by avian and mammalian vectors. The skin provides volatile and nonvolatile aroma and color compounds, the pulp contributes organic acids and sugars, and the seeds provide condensed tannins, all of which are important to the formation of organoleptic characteristics of wine. In order to understand the transcriptional network responsible for controlling tissue-specific mRNA expression patterns, mRNA expression profiling was conducted on each tissue of mature berries of *V. vinifera *Cabernet Sauvignon using the Affymetrix GeneChip^® ^*Vitis *oligonucleotide microarray ver. 1.0. In order to monitor the influence of water-deficit stress on tissue-specific expression patterns, mRNA expression profiles were also compared from mature berries harvested from vines subjected to well-watered or water-deficit conditions.

**Results:**

Overall, berry tissues were found to express approximately 76% of genes represented on the *Vitis *microarray. Approximately 60% of these genes exhibited significant differential expression in one or more of the three major tissue types with more than 28% of genes showing pronounced (2-fold or greater) differences in mRNA expression. The largest difference in tissue-specific expression was observed between the seed and pulp/skin. Exocarp tissue, which is involved in pathogen defense and pigment production, showed higher mRNA abundance relative to other berry tissues for genes involved with flavonoid biosynthesis, pathogen resistance, and cell wall modification. Mesocarp tissue, which is considered a nutritive tissue, exhibited a higher mRNA abundance of genes involved in cell wall function and transport processes. Seeds, which supply essential resources for embryo development, showed higher mRNA abundance of genes encoding phenylpropanoid biosynthetic enzymes, seed storage proteins, and late embryogenesis abundant proteins. Water-deficit stress affected the mRNA abundance of 13% of the genes with differential expression patterns occurring mainly in the pulp and skin. In pulp and seed tissues transcript abundance in most functional categories declined in water-deficit stressed vines relative to well-watered vines with transcripts for storage proteins and novel (no-hit) functional assignments being over represented. In the skin of berries from water-deficit stressed vines, however, transcripts from several functional categories including general phenypropanoid and ethylene metabolism, pathogenesis-related responses, energy, and interaction with the environment were significantly over-represented.

**Conclusion:**

These results revealed novel insights into the tissue-specific expression mRNA expression patterns of an extensive repertoire of genes expressed in berry tissues. This work also establishes an extensive catalogue of gene expression patterns for future investigations aimed at the dissection of the transcriptional regulatory hierarchies that govern tissue-specific expression patterns associated with tissue differentiation within berries. These results also confirmed that water-deficit stress has a profound effect on mRNA expression patterns particularly associated with the biosynthesis of aroma and color metabolites within skin and pulp tissues that ultimately impact wine quality.

## Background

The grape berry is considered to be mainly a sink for primary metabolites essential for plant survival (e.g., water, sugar, amino acids, minerals and micronutrients) [1]. However, the berry also synthesizes the major determinants of the aroma, color and flavor of wine. The three major tissues of the berry play different roles in seed dissemination, each helping to perpetuate grape species. This specialization has a direct impact on wine quality because each tissue contributes specific organoleptic properties to wine. The fruit exocarp (skin) contributes to the integrity of the whole berry by protecting inner tissues against mechanical damage or pathogen attack [2]. The color of the berry skin promotes seed dispersion by providing a high contrast between background foliage and fruits [3] as well as providing protection from UV light exposure [4]. Aromas are also involved in seed dispersion in that they serve as important attractants to dispersal agents when fruits are ripe and ready to consume, but are more susceptible to pathogen attack [5]. Aromas arise from volatile compounds, such as terpenes, norisoprenoids, and thiols stored as sugar or amino acid conjugates in the vacuoles of exocarp cells [6]. However, some volatile compounds accumulate differentially between the exocarp and mesocarp [7]. Biosynthesis of many volatile aroma compounds is induced after the initiation of berry ripening [8-11]. For wine making, the variability of skin composition plays an important role in determining the color, aroma, and other organoleptic properties of wine. The primary role of the pulp is to provide a high value nutritional content for dispersal agents, including high concentrations of free amino acids and hexose sugars. During wine production, the pulp tissue contributes the majority of sugars, which are transformed into alcohol during the fermentation process. The seed, which contains the embryo, is the main source of flavan-3-ol monomers and procyanidins (seed tannins). These compounds contribute important organoleptic properties of wine including bitterness and astringency [12].

Few studies have addressed tissue-specific gene expression patterns in the grape berry. The intercellular distribution of enzymes involved in amino acid, organic acid, and sugar metabolism was examined in developing grape seeds [13] and berries [14] using immunohistochemical localization analysis to assess the metabolism of nitrogenous assimilates and compartmentation of key processes in sugar, organic acid and amino acid metabolism. The mRNA expression patterns of genes encoding flavonoid 3'- and flavonoid 3',5'-hydroxylase, and dihydroflavonol 4-reductase (DFR) genes, key enzymes of the phenylpropanoid pathway, were followed in developing berries and found to be expressed predominantly in the seeds and skin of pre- and post-véraison berries [15,16]. Expression patterns of these 3'- and 3',5'-hydroxylase genes correlated well with the accumulation patterns of cyanidin (red)- and delphinidin (blue)-based anthocyanins. Using immunoelectron microscopy, enzymes of the phenylpropanoid pathway, such as phenylalanine ammonia-lyase (PAL) and 4-coumarate:coenzyme A ligase (4CL), were localized to the cell walls, parenchyma, and vascular tissues of the mesocarp close to the pericarp, whereas cinnamate-4-hydroxylase (C4H) was found primarily in the plastids and nuclei of this tissue [17]. *Vitis vinifera *mutations affecting tissue-specific characteristics of grapevine are rare. However, a fleshless berry mutant has been described recently that promises to be a useful tool towards understanding the molecular genetic basis of tissue-specificity within the context of berry development [18].

Functional genomic resources for *Vitis vinifera *and related species have proliferated rapidly within the last several years mainly in the form of large, publicly available expressed sequence tag (EST) databases [19,20]. The availability of such information has permitted large-scale mRNA expression profiling studies of gene expression profiles of flowers and berry skin development using cDNA or oligonucleotide microarrays [21-23]. Comprehensive transcript profiling using high-density microarrays has recently been used to investigate the effect of water-deficit and isoosmotic salinity stress on grapevine shoot tissues [24]. However, neither the tissue-specific expression patterns within berry tissues nor the effect of water-deficit stress on these tissues have been investigated in *V. vinifera*.

Regulated-deficit irrigation has been used advantageously to inhibit vine growth without fruit yield reductions, and make measurable improvements in grape quality, namely through increases in the total phenolic and anthocyanin content of fruit [25,26]. These compounds are major components of the sensory characteristics of wine, resulting in improved aroma, color and flavor, as well as enhancing the health benefits conferred by polyphenolic compounds with antioxidant activity [27]. In this study we report the large-scale mRNA expression profiles of three berry tissues using the GeneChip^® ^*Vitis vinifera *(Grape) Genome Array. Skin, pulp, and seeds of well-watered and water-deficit stressed vines of Cabernet Sauvignon were all profiled. Tissue-specific and water-deficit stress-induced changes in mRNA abundance have been identified that indicate which gene expression changes are important for alterations in organoleptic properties of wine produced from grapes subjected to water-deficit stress irrigation.

## Results and discussion

### Physiological results

Mature berry samples were harvested concurrently with the commercial harvest of the vineyard corresponding to stage 38 [28]. The stem water potential differences between well-watered plants and water-deficit plants were monitored as a comprehensive indication of water-deficit in the vines [29]. Stem water potentials were significantly more negative for water-deficit treated than for well-watered vines at the time of harvest (Table 1). Physiological data were used to confirm the developmental stage of the berry samples and to document the physiological differences between berries from well-watered and water-deficit stress treated vines. Brix values, an approximate measure of the mass ratio of dissolved solids (mostly sucrose) to water in fruit juices, were significantly different between the well-watered and water-deficit stress treated vines with water-deficit resulting in a 6° increase in refractive index. The increase in Brix for water-deficit-treated berries was most likely due to dehydration of the fruit as berry shrivel was observed. These values bracket the generally recommended value for harvest for Cabernet Sauvignon in California (i.e., 23° Brix). Berry diameter of fruit from well-watered vines was also found to be significantly larger than that of berries harvested from water-deficit stress treated vines (Table 1).

**Table 1:** Physiological data for berries harvested from vines grown under well-watered and water-deficit conditions.

Sample	Stem water potential (mPa)	Berry refractive index (°Brix)	Berry diameter (mm)
Well-watered vine	-0.70 ± 0.11^a^	19.97 ± 1.67^a^	12.30 ± 0.20^c^
Water-deficit vine	-1.08 ± 0.15^a^	26.14 ± 2.5^b^	10.90 ± 0.33^c^
T-test P-value	<0.001	<0.0001	<0.008

^a ^n = 6, ^b^n = 5, ^c^n = 90; ± standard deviation.

### Comparisons among berry tissues

The mRNA expression profiles of pulp, skin and seed were compared using the Affymetrix GeneChip^® ^*Vitis *genome array ver. 1.0. Testing was performed using biological triplicates for three tissue-specific contrasts under well-watered conditions and on the water status contrasts for each tissue type. A visual inspection of the distributions of raw perfect match (PM) probe-level intensities for all 18 arrays showed that pre-processed and normalized PM intensities using Robust Multi-Array Average (RMA) [30] were consistent across all arrays with no apparent outlying arrays. Digestion curves describing trends in RNA degradation between the 5' end and the 3' end in each probe set were examined and all 18 proved very similar. Correlations among biological replicates were good: Spearman coefficients ranged from 0.966 to 0.994; Pearson coefficients ranged between 0.982 and 0.993.

**Figure 1:**
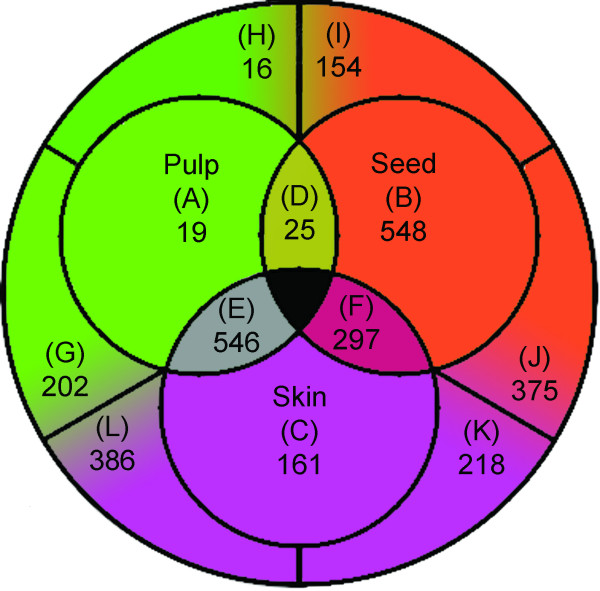
**Venn diagram of the 12 clusters of significantly differentially expressed probesets across tissues under well-watered conditions**. Clusters A, G, H correspond to significantly greater transcript abundance accumulation in pulp. Clusters B, I, J correspond to significantly greater transcript abundance accumulation in seed. Clusters C, L, K correspond to significantly greater transcript abundance accumulation in skin. Clusters F, J, K correspond to significantly lesser transcript abundance accumulation in pulp. Clusters E, G, L correspond to significantly lesser transcript abundance accumulation in seed. Clusters D, H, I correspond to significantly lesser transcript abundance accumulation in skin.

From the 14,650 *Vitis *genes represented on the array, 11,149 were found to be present in at least one of the three tissues for an overall mean call rate of 64%  per array (range 57%–69%). Of these, 6,683 genes (60%) were found to have statistically significant differential expression (p < 0.05) amongst the three tissues under well-watered conditions only. Of these, 3,191 probesets (28%) were shown to be differentially expressed amongst the three tissues with a two-fold or greater change in mRNA abundance. Thresholds of 1.5-fold and 1.75-fold ratios resulted in 5,337 and 4,021 probesets, respectively. Redundant probe sets representing the same tentative consensus (TC) sequences or Unigene were removed and the 2,947 genes with significant differential expression patterns of 2-fold or greater were grouped into 12 clusters by hierarchical clustering using the complete agglomeration method and Euclidean distance metric according to mRNA expression within each tissue in comparison with the other two (Figure 1; Additional file 1). This method was used as opposed to correlation-based clustering approaches (i.e., Pearson correlation) because it resulted in the best resolution of 12 distinct clusters illustrating tissue-specific expression patterns. mRNAs preferentially expressed in pulp (A), seed (B) and skin (C) clusters corresponded to 19, 548, and 161 Unigenes, respectively. The clusters D, E and F represented mRNA expressed in one tissue, but having similar expression levels in one of the other tissues. Cluster D corresponds to mRNAs that were under-expressed in skin (25 Unigenes); cluster E corresponds to mRNAs that were under-expressed in seed (546 Unigenes); cluster F corresponds to mRNAs that were under-expressed in pulp (297 Unigenes). Clusters G to L correspond to mRNAs that were differentially expressed in pulp and skin tissues, respectively. The largest difference in tissue-specific expression was between seed and pulp/skin where 2,394 (75%) of the Unigenes showed significant differentially expression between these two tissues. 1,231 probe sets were under-expressed in seed (clusters E, G, L) and 1,163 were over-expressed in seed (clusters B, I, J) (Figure 1). In contrast, far fewer genes exhibited differential mRNA expression in the pulp (38%; 250 probe sets over-expressed, clusters (A, G, H); 969 probe sets under-expressed, clusters F, J, K) and in the skin (33%; 835 probe sets over-expressed, clusters C, K, L; 209 probe sets under-expressed, clusters D, H, I). Skin tissue shared the highest proportion of genes, expressed in common with other tissues (74%), whereas the seed and pulp tissue shared only 55% and 46% of genes respectively, expressed in common with other tissues.

### Quantitative real-time RT-PCR

**Figure 2:**
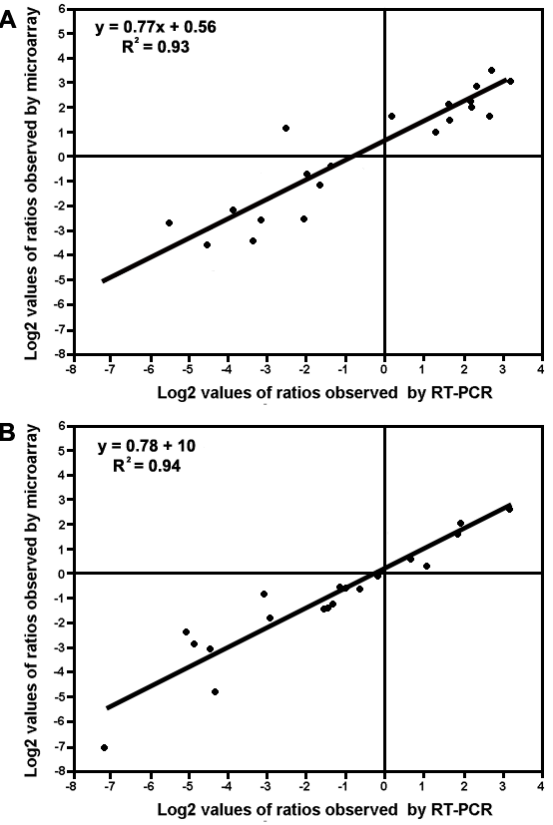
**Comparison of gene expression ratios reported by the Affymetrix GeneChip^® ^Vitis oligonucleotide microarray and by quantitative real-time RT-PCR**. Data were from 19 probesets and three different tissues (pulp, skin and seed) from well-watered vines including comparisons from two probesets and two tissues (pulp and skin) between well-watered and water-deficit stressed vines. The microarray log_2 _(expression ratio) values (y-axis) are plotted against the log_2 _(expression ratio) obtained by quantitative real-time RT-PCR (x-axis). A) Comparisons between pulp and skin. B) Comparisons between pulp and seed. Linear regression analysis showed an overall coefficient of variation of 0.93 and 0.94, respectively (formula insets).

To validate expression profiles obtained using the Affymetrix GeneChip^® ^*Vitis *oligonucleotide microarray, quantitative RT-PCR was performed on 19 genes using gene-specific primer pairs [Additional file 2]. Transcript abundance patterns were compared among tissues including two genes for which comparisons were confirmed between pulp and skin or pulp and seed tissues from berries harvested from well-watered and water-deficit stressed vines (Figure 2). Linear regression ([microarray value] = a [RT-PCR value] +b) analysis showed coefficients of variation of 0.93 or 0.94. However, significant differences were observed for several of these genes depending on the tissue examined. The transcript abundance in the seed for germin (TC45186) and nine-cis-epoxycarotenoid dioxygenase (NCED) (TC42536) were disproportionately higher when estimated by microarray.

### Functional categorization of differentially expressed genes among berry tissues

Functional categories of the genes in each tissue type were determined by amino acid sequence comparisons with *Arabidopsis thaliana *proteins as described previously [31]. Table 2 presents results from the top-level Munich Information Center for Protein Sequences (MIPS) functional categories (Table 2a) and sub-categories for the metabolism category (Table 2b) for the pulp (A, G, H), seed (B, I, J) and skin (C, L, K), clusters described above. Each column lists the relative number of transcripts (%) or "transcript fingerprint" more than two-fold over- or under-represented within each functional category by each specific tissue type. As a reference, the last column corresponds to the percentages of genes present within each categories of the grape Unigenes set from the Dana-Farber Cancer Institute Grape Gene Index [32], which includes ESTs from a wide range of organs and developmental stages organized into Unigenes. The skin transcript expression profile presented the most distant fingerprint from the global set, wherein the categories related to housekeeping processes, such as protein fate, cell fate, cell cycle and DNA processing, protein synthesis and activity, were under-represented. In contrast, functional categories including transport facilitation, energy, and amino acid metabolism were over-represented. Within the metabolism category, the subcategories of secondary, lipid, and amino acid metabolism were over-represented in this tissue. The pulp also exhibited a distinctive profile of disproportionately high relative abundance of transcripts encoding carbohydrate and secondary metabolism, whereas transcripts encoding transport facilitation, energy functions along with genes involve in secondary metabolism, lipid, amino acid biosynthesis, vitamins and cofactors, and nitrogen and sulfur metabolism exhibited disproportionately low relative abundance. In seeds, transcripts encoding secondary, amino acid, and nitrogen and sulfur metabolism were over-represented.

**Table 2:** Functional categories of the differentially expressed (p ≤ 0.05, 2-fold) mRNA among berry tissues from well-watered vines

**Functional category**	**% of Unigenes over expressed in:**			**% of Unigenes under expressed in:**			**% total in probesets**
	
*a) Top level*	Pulp	Skin	Seed	Pulp	Skin	Seed	
		
Protein with binding function	27.0 (64)	*20.1 (154*)	*25.0 (269)*	*21.9 (195)*	26.7 (52)	24.8 (281)	26.3 (3588)
Metabolism	27.0 (64)	**31.5 (241)**	**27.4 (295)**	**31.9 (284)**	28.2 (55)	25.7 (291)	20.9 (2851)
Protein fate	16.0 (38)	*12.2 (93)*	17.2 (185)	*12.4 (110)*	22.0 (43)	16.3 (185)	16.7 (2270)
Signal transduction mechanism	13.5 (32)	15.7 (120)	*11.0 (118)*	*11.7 (104)*	15.9 (31)	**18.4 (209**)	13.6 (1847)
Transcription	15.2 (36)	13.2 (101)	13.7 (148)	11.5 (102)	11.8 (23)	16.1 (183)	12.9 (1762)
Cell rescue, defense and virulence	10.5 (25)	**17.4 (133)**	**15.7 (169)**	**15.5 (138)**	14.4 (28)	12.9 (146)	11.1 (1509)
Development	13.9 (33)	11.8 (90)	12.1 (130)	*9.5 (85)*	9.7 (19)	13.2 (150)	11.8 (1611)
Cell fate	12.6 (30)	*8.6 (66)*	*9.4 (101)*	*8.5 (76)*	10.3 (20)	10.3 (117)	11.7 (1593)
Control of cellular organization	10.6 (25)	10.1 (77)	9.7 (105)	10.0 (89)	9.2 (18)	10.6 (120)	9.5 (1296)
Interaction with cellular environment	10.1 (24)	8.4 (64)	11.4 (123)	8.8 (78)	13.8 (27)	8.8 (100)	8.5 (1158)
Cell cycle and DNA processing	8.4 (20)	*4.4 (34)*	7.6 (82)	*3.9 (35)*	11.3 (22)	7.0 (80)	8.4 (1145)
Transport facilitation	7.2 (17)	**15.6(119)**	10.6 (114)	**15.4 (137)**	11.3 (22)	9.3 (106)	8.6 (1174)
Cellular transport	9.3 (22)	6.3 (48)	*5.6 (60*)	6.8 (60)	5.1 (10)	8 (91)	7.2 (982)
Protein synthesis	*3.8 (9)*	*2.6 (20)*	8.1 (87)	7.6 (68)	5.1 (10)	*3.4 (39)*	7.5 (1017)
Energy	4.6 (11)	**11.0 (84)**	8.4 (91)	**11.9 (106)**	9.2 (18)	6.9 (78)	7.0 (947)
Interaction with environment	8.9 (21)	7.8 (60)	9.3 (100)	6.6 (59)	11.3 (22)	8.9 (101)	7.0 (956)
Protein activity regulation	4.6 (11)	2.3 (18)	3.0 (32)	2.5 (22)	6.7 (13)	2.6 (29)	3.5 (478)
Cell type differentiation	3.0 (7)	2.0 (15)	2.1 (23)	1.6 (14)	2.6 (5)	1.8 (21)	1.9 (257)
Storage protein	0 (0)	0 (0)	0.7 (8)	0.1 (1)	0 (0)	0.1 (1)	0.3 (45)
Unknown function	16.9 (40)	11.0 (84)	11.0 (118)	11.5 (102)	14.4 (28)	13.5 (153)	9.6 (1306)
No hit	24.1 (57)	21.2 (162)	26.4 (284)	22.6 (201)	28.7 (56)	21.5 (244)	34.2 (4656)
*b) Metabolism*							
Carbohydrates	**14.8 (35)**	12.9 (99)	11.0 (118)	12.1 (108)	7.7 (15)	11.3 (128)	8.6 (1169)
Secondary metabolism	**9.3 (22)**	**9.4 (72)**	**8.7 (93)**	**10.4 (93)**	8.2(16)	6.5 (74)	4.8 (652)
Lipids	3.4 (8)	**8.4 (64)**	5.8 (62)	**8.5 (76)**	4.6 (9)	5 (57)	4.6 (632)
Amino acids	1.7 (4)	**6.5 (50)**	**6.3 (68)**	**7.4 (66)**	6.7 (13)	4.9 (55)	3.8 (511)
Vitamins and cofactors	3.8 (9)	3.9 (30)	3.3 (36)	**5.4 (48)**	1.5 (3)	2.4 (27)	2.4 (326)
Phosphate	3.4 (8)	4.3 (33)	2.7 (29)	2.1 (19)	4.6 (9)	4.3 (49)	3.2 (433)
Nucleotides	4.6 (11)	2.6 (20)	3.5 (38)	3.7 (33)	3.1 (6)	2.7 (31)	2.9 (401)
Nitrogen and sulfur	0.4 (1)	1.8 (14)	**3.1 (33)**	**3.3 (29)**	3.6 (7)	1.3 (15)	1.4 (184)

Unigenes were grouped according to Figure 1. The functional categories were assigned according to the MIPS functional catalog as a) top level categories and b) second level categories within the metabolism category. The values in parentheses correspond to the total number of Unigenes observed in each category. The last column corresponds to the relative percentage within each category of all 13,622 Unigenes present on the *Vitis *Genechip^® ^genome array. Typefaces indicate functional categories that are significantly over- (bold) or under- (italic) represented when ompared to the overall distribution of all 13,622 Unigenes using a Fisher's exact test. Underscores indicate over- or under-representation, respectively, of more than 1.5-fold the percentage expected in that category based on the overall distribution of all *Vitis *Unigenes.

### Tissues-specific mRNA expression of flavonoid pathway genes

**Figure 3:**
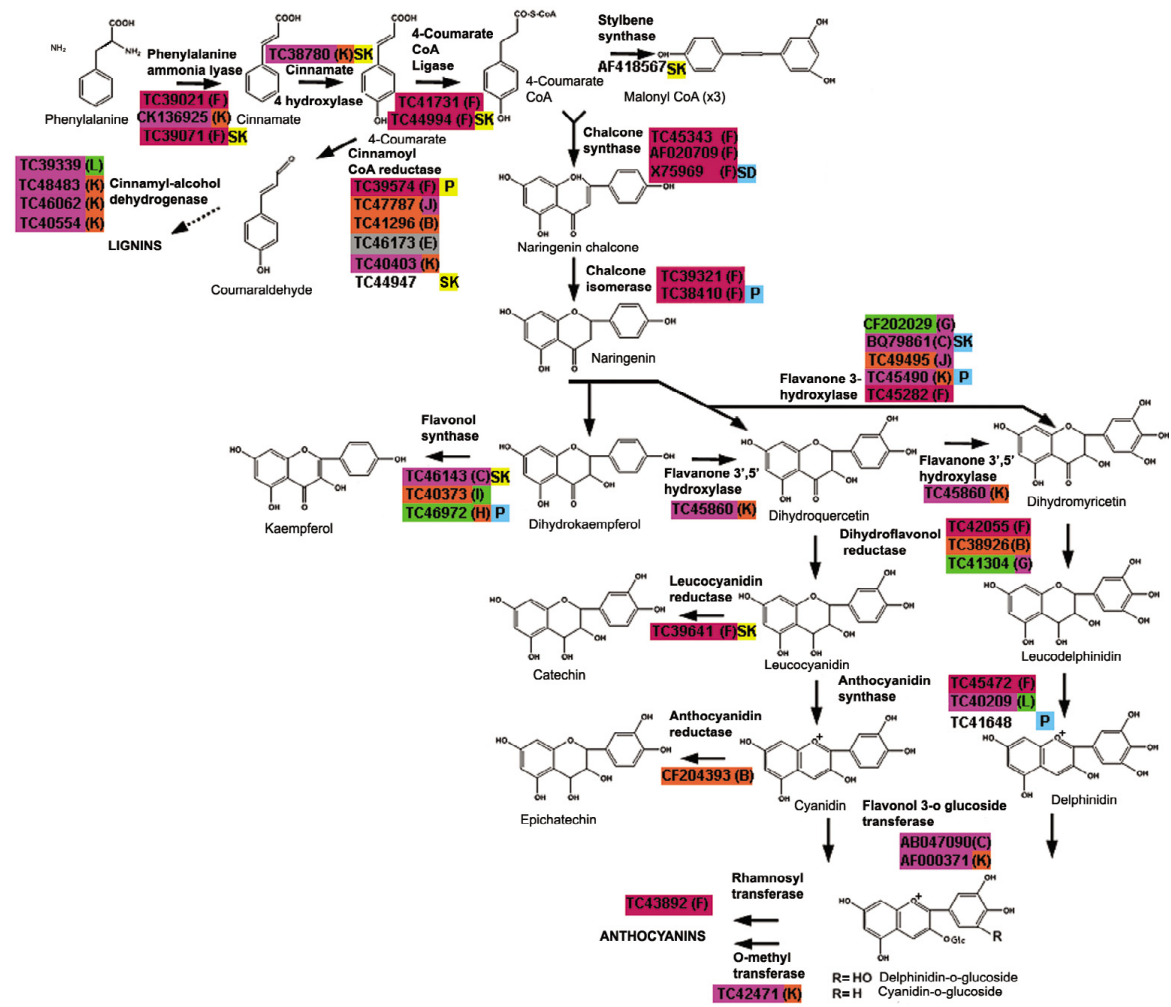
**mRNAs differentially expressed across tissues under well-watered and water-deficit stress within a simplified flavonoid biosynthetic pathway**. Color codes correspond to those used in Figure 1: Purple (skin-specific), green (pulp-specific), and brown (seed-specific). Letters in parentheses following the Unigene name correspond to the cluster assignment. Intermediates colors indicate genes expressed equally within two tissues. The three genes that lack color-coding were not differentially expressed. Yellow and blue squares indicate mRNAs over-expressed under water-deficit and well-watered tissues, respectively, with in tissue type indicated inside the square (P: pulp; SK: skin; SD: seed).

Phenolic compounds are the major wine constituents responsible for organoleptic properties such as color and astringency. Moreover, a majority of phenolic compounds in wine are derived from flavonoids (tannins). For red grapes, roughly 30–40% of the total phenolic content is located in the skins and 60–70% in the seeds [33]. Thirty-nine Unigenes encoding biosynthetic enzymes of the flavonoid pathway were found to exhibit differential mRNA expression patterns among different berry tissues (Figure 3; Additional file 3, Table 1). Consistent with the location of accumulation of flavonoid pigments, the majority of the genes encoding flavonoid biosynthetic enzymes were specifically expressed in the skin or seed (skin > seed >> pulp).

### General phenylpropanoid pathway genes

For the first eight enzymatic steps of the initial phenylpropanoid pathway from phenylalanine ammonia lyase (PAL) to flavanone-3-hydroxylase (F3H), and then leading to anthocyanin and tannin biosynthesis, 15 of 16 Unigenes identified exhibited skin-specific or skin-preferential mRNA expression, with only one enzyme (F3H) encoded by a Unigene (CF202029) showing pulp-specific expression (Figure 3; Additional file 3, Table 1). Two F3H-related dioxygenases, also homologous to an ethylene-forming enzyme, were expressed predominantly in the skin (BQ798614) or seed (TC49495), whereas one F3H isozyme (CF202029) was expressed specifically in the pulp. Skin- (BQ798614) or seed-specific (TC49495) expression of these F3H isozymes may affect the final flavonoid composition in specific tissues. The specific activity of these potential F3H isozymes must be determined definitively.

Hydroxylation of dihydrokaempferol on the 3' or 5' position of the B-ring by flavanoid 3' hydroxylase (F3'H) and/or flavanoid 3'5' hydroxylase (F3'5'H) yields precursors for orange (pelargonidin), red-magenta (cyanidin), and purple-mauve (delphinidin) anthocyanidin pigments. The specific expression of the F3'H/F3'5'H (TC45860) in the skin suggests that the enzyme encoded by this gene may account for the high proportion of dihydromyricetin-derived anthocyanidins in skin [34], and of dihydroquercitin-derived flavan-3ols in seed (Figure 3). Tannins derived from dihydroquercitin are found predominantly in the seed [35,36]. Surprisingly, none of the dihydroflavonol reductase isozyme genes were found to be skin-specific, with one Unigene being expressed in both skin and seed (TC42055), one specifically in the seed (TC38926), and one specifically expressed in the pulp (TC41304).

### Lignin biosynthesis pathway

The biosynthesis of monolignols requires two enzymatic steps that branch off the general phenylpropanoid pathway and are catalyzed by cinnamoyl-CoA Reductase (CCR) and cinnamyl alcohol dehydrogenase (CAD). CCR, a key enzyme in the formation of lignin monomers, plays a major role in determining total lignin content and quality of soluble phenolic content in tomato [37]. Of the five isogenes of CCR identified, only two genes showed seed-specific expression (TC41296 and TC47787) and one showed skin-specific expression (TC40043), whereas two others exhibited mixed expression in the pulp/skin or skin/seed (Figure 3; Additional file 3, Table 1). The four CAD isozymes all showed preferential mRNA accumulation in skin (TC48483, TC46062, TC40554 and) or skin/pulp (TC39339). These expression patterns may be related to vascular bundle formation, which occurs specifically in these tissues as observed in strawberry [38]. However, CADs may also be involved in the synthesis of cinnamyl alcohol derivatives responsible for fruit flavor and aroma [39].

### Flavonol biosynthesis pathway

Three different isogenes encoding flavonol synthase (FLS), the enzyme responsible for the conversion of dihydroflavonols to flavonols, were identified with each being expressed preferentially in the three different tissues examined (Figure 3; Additional file 3, Table 1). Flavonols (e.g., quercetin, kaempferol, and myricetin) are important co-pigments thought to stabilize the anthocyanin pigments in wine. The skin-specific isogene (TC46143) corresponds to VvFLS1 described previously [15,40] and has the same expression pattern described in these earlier studies. A second isoform (AY257979) described in [40] was barely detectable during berry development and showed very low expression in the present study as well. Two additional FLS isoforms were expressed preferentially in the pulp (TC46972) and seed (TC40373). As quercetin does not accumulate in the seed or pulp in detectable amounts [40], these isoforms are likely to encode dioxygenase activities for other dihydroflavonol substrates.

### Anthocyanidin biosynthesis pathway

Two genes were identified that encoded anthocyanidin synthase (ANS) or leucoanthocyanidin dioxygenase (LDOX), the enzyme responsible for the formation of UV-absorbing anthocyanins (e.g., cyanidin, dephinidin) (Figure 3; Additional file 3, Table 1). One isogene was expressed predominantly in the skin (TC45472) consistent with earlier observations [15,41]. However, a second isogene was expressed in both skin and pulp (TC40209), but neither gene showed expression in seeds of ripe berries, as also previously observed [41]. UDP-glucose:flavonoid 3-O-glucosyltransferase (UFGT), which catalyzes the formation of anthocyanin glucosides, has only been reported to be expressed in red grape varieties following véraision [34,41,42]. Two isogenes encoding this enzyme (AF000371 and AB047090) were expressed predominantly in skin tissues of ripe berries (Figure 3; Additional file 3, Table 1). Five additional glucosyl transferases were differentially expressed in this experiment (data not shown), but their relationship with the anthocyanin biosynthetic pathway is not clear. Surprisingly, we also identified a gene encoding anthocyanidin rhamnosyltransferase (RT) (TC43892), which was expressed in the seed and skin, because the presence of diglucanosylated anthocyanidins has not been reported for *V. vinifera*. However, other *Vitis *species, such as *V. rotundifolia*, do accumulate this type of decorated anthocyanidin [43]. One O-methyltransferase gene (TC42471) having a potential role in anthocyanidin methylation [44] was expressed largely in the skin. No genes with tissue-specific expression patterns encoding 5-glucosyl-acyltransferases, which are responsible for the further substitution of anthocyanin-3-glucosides, were identified [45].

#### Proanthocyanidins biosynthesis

Two enzymes, leucoanthocyanidin reductase (LAR), which catalyzes the formation of catechin-derived compounds [46], and anthocyanidin reductase (ANR), which catalyzes the formation of epicatechin-derived compounds [47], have been identified in plants. Only a single LAR isogene (TC39641) was expressed differentially in the seed and skin. The expression of TC39641 was significantly low in pulp and the differential expression observed between seed and skin was not significant. However, this difference was validated to be significantly higher in seeds than in skin by real-time qRT-PCR (Figure 2). A second isogene encoding LAR (TC47624) did not appear to be expressed in ripe grape berry (data not shown) consistent with previous studies [41]. The harvest date chosen in the present study was not optimal for studying tannin biosynthesis-related mRNA expression, because LAR gene expression and tannin accumulation occurs predominantly during the very early stages of berry development up to two weeks prior to véraision [35,41]. The mRNA expression of the ANR (CF204393) clearly showed that this Unigene is seed-specific (Figure 3; Additional file 3, Table 1) and detectable well after véraison, in contrast to earlier studies [41] where this gene was detectable by qRT-PCR up to 2–3 weeks post-véraison.

### Cell wall metabolism

The composition of grape berry cell walls is of interest due to their importance in wine production, as wine polysaccharides play important roles in the intrinsic organoleptic (e.g., mouth-feel) properties of wine [48]. During berry development modifications of the cell wall allow cell expansion within the skin and the pulp tissues. Cellular multiplication occurs during a short period of time during very early stages of berry development (e.g., stage 27) [28]. Thereafter, berry growth is due to cell expansion alone, which can account for more than a 300-fold increase in the volume of mesocarp cells during berry enlargement. Within the skin and pulp, the cell wall undergoes depolymerization after véraision, and most differentially expressed mRNAs were observed in one or both of these tissues (Additional file 3, Table 2). In particular, galacturonan content increases and becomes more soluble as ripening progresses, whereas arabinogalactan type I content decreases [49]. Activities and mRNA expression of several cell wall enzymes are known to increase during berry development including β-galactosidase, pectin methylesterase, polygalacturonidase, pectate lyase, and xyloglucan endotransglycosylase [50]. Modification of the cell wall in seeds also occurs at germination and is not considered here.

Cell wall constituents in grape berry skin and pulp have been analyzed quantitatively for pectic polysaccharides [51] and for xyloglucans [52]. Differences in pectin composition have been observed between the skin and the pulp [51]. Seventy-five percent of the berry pectin is located in the skin, whereas it represents only 25% of the total fresh berry weight [51]. Although glycosyl-residue composition of the three types of pectin (e.g., homogalacturonans, rhamnogalacturonan type I (RG-I), and rhamnogalacturonan type II (RG-II) cell wall material are similar between pulp and skin tissues, pulp tissues contain two-fold more buffer-soluble arabinogalactan proteins (AGPs) and pectin than skin tissues [51]. The expression of pulp-specific polygalacturonase (AY043233, TC47738) and skin-specific polygalacturonase (TC46487), pectate lyase (TC45703), and pectin methylesterase (TC47054) could be related either to depolymerization of common pectic polysaccharides occurring specifically within each tissue, or to the depolymerization of tissue-specific polysaccharides (Additional file 3, Table 2). mRNAs encoding pectin modification enzymes were not strongly expressed in seed suggesting that these activities are not important in this tissue, with the exception of one pectinesterase (TC46866) and two pectin methylesterase inhibitor genes (CF373209, TC48184). The exact role of these proteins in pectin depolymerization remains unknown.

Expansins play important roles in cell wall loosening via non-enzymatic mechanisms and are involved in cell expansion and other developmental events wherein cell-wall modification occurs [53]. Several expansin genes were specifically expressed in the skin (TC38403, BQ794765, TC38813, and TC46110), whereas others were preferentially expressed in the pulp (TC38812) or seed (TC40522, TC46149). In grape berries, xyloglucans are noncellulosic polysaccharides that account for about 8% of cell wall polysaccharides in the pulp and skin. Xyloglucans isolated from mesocarp and exocarp cell walls of grape berries are composed of seven types of oligosaccharides in similar proportions in skin and pulp, except for XXFG, which is more abundant in pulp, and XLFG, which is more abundant in skin [51]. Xyloglucan endotransglycosylases, which hydrolyze and transglycosylate xyloglucans, were encoded by multiple isogenes expressed predominantly in the pulp (TC46220, TC45396, and AB074999) or skin (TC38792). However two isogenes were expressed specifically in the seed (TC45154, CF206328). Glucan endo-1,3-beta-glucosidase gene expression occurred primarily in the pulp and skin, whereas Beta-D-galactosidase (TC46813) expression occurred mainly in the seed (Additional file 3, Table 2). Three isogenes encoding cellulases were also identified that were expressed preferentially in the pulp and skin tissues (TC45088, TC45090, and TC47657). Cellulase isogene expression has not been described previously in ripe berries [49].

### Photosynthesis and carbon assimilation

During berry development, transcripts encoding proteins with photosynthesis-related functions are most strongly expressed at flowering and two-weeks after flowering and then decline steadily in abundance throughout berry development [21,22]. However, the skin of ripe berries still contained a high proportion of transcripts for Unigenes encoding proteins with functions related to photosynthesis and carbon assimilation relative to the pulp and the seed (Additional file 3, Table 3). In particular, the skin showed greater expression of several light harvesting and photosystem components relative to other tissues. However, the pulp showed high expression of a light harvesting pigment protein (TC47896), and the seed showed high expression of two subunits of the photosystem II oxygen evolving complex (TC38964, TC44135). Many transcripts encoding enzymes of the Calvin-Benson cycle were also expressed preferentially in the skin, although some isogenes appeared to be preferentially expressed in the seed (e.g., pyruvate kinase, TC42635; glycerate-3-phosphate dehydrogenase, TC40753; and fructose-1,6-bisphosphatase TC40284). Similarly, transcripts for several enzymes involved in malate biosynthesis, a major determinant of titratable acidity in grape, remain highly expressed in the skin. These expression patterns are consistent with the observation that after véraison malate concentrations increase in the skin, but decrease in other more internal, concentric regions of the berry [54]. Interestingly, pyruvate, orthophosphate dikinase (TC46453), a plastid-localized enzyme, is strongly expressed in the seed (Additional file 3, Table 3). In contrast to the overall breakdown in total malate after the onset of berry ripening, tartrate content remains relatively constant, but the innermost concentric regions surrounding the seed contained the highest tartrate concentrations [54]. Consistent with this observation, tartrate synthase transcripts (L-iditol 2-dehydrogenase, TC45858) were more abundant in seeds than in outer mesocarp and skin tissues (Additional file 3, Table 3).

### Isoprenoid metabolism

The isoprenoid pathway produces monoterpenes (C_10_), isopentenyl-pyrophophate (IPP) and its isomer, dimethylallyl pyrophosphate (DMAPP), are responsible for the formation of many volatile aroma precursors arising mainly from berry exocarp tissues. Compounds related to this metabolic pathway are present mainly in the exocarp. Therefore, most mRNAs involved in the isoprenoid pathway showed expression in both skin and pulp. For example, mRNAs involved in mevalonate biosynthesis were expressed preferentially in both skin and pulp tissues (TC49029, TC45366, TC47115, TC40923). IPP biosynthesis via the non-mevalonate pathway involves 1-deoxy-D-xylulose 5-phosphate synthase (TC48118), which was expressed primarily in the skin (Additional file 3, Table 4). Isopentenyl diphosphate isomerase (TC38778), which catalyzes the conversion of IPP to dimethylallyl diphosphate to form the basic C5 isoprene unit for IPP condensation, was preferentially expressed in the pulp and skin similar to other mevalonate pathway genes. In contrast, geranylgeranyl diphosphate synthase (TC40542), which catalyzes condensation of IPP to geranylgeranyl diphosphate (GGPP), was expressed in the seed.

Grape seed is known to contain a high concentration of sterols [55]. Squalene synthase (SS) (TC39028), which catalyzes the first committed step in sterol and triterpenoid biosynthesis, was expressed in both the seed and skin. In contrast, transcripts for squalene monooxygenases (TC40984 and TC43543) were preferentially expressed in the seed. Terpene synthases, which catalyze the formation of volatile compounds from C5, C10, C15, and C20 terpene skeletons from allylic prenyl diphosphate intermediates of the terpene biosynthetic pathway, are important for the production of floral sesquiterpene volatiles [56]. Terpene synthase (TC42197) and E-beta-ocimene synthase (TC40259) were preferentially expressed in the pulp suggesting that this is the formation site of these volatile compounds. Volatile metabolite analysis of this tissue will be needed to confirm this suggestion.

### Carotenoid and abscisic acid metabolism

Carotenoids are found mainly in the skin at levels that are typically 2–3 times that of those found in the pulp. Consistent with these localization patterns, the genes encoding carotenoid biosynthesis and catabolism showed mostly skin-specific expression patterns. Phytoene synthase (TC46134), which catalyzes the condensation of GGPP (C_20_) to form phytoene (C_40_), the basic precursor of all carotenoids, was expressed primarily in the skin (Additional file 3, Table 4). Zeta-carotene desaturase (TC48107), which catalyzes the symmetrical formation of two double bonds at each side of the molecule in a cis configuration leading to the formation of lycopene [57], was expressed in both the skin and the pulp. In contrast, beta-carotene hydroxylase (TC42069), which catalyzes the hydroxylation at the C-3 positions of both β- and ε-rings of carotenoid substrates leading to the formation of lutein, the most abundant carotenoid in plant photosynthetic tissues [58], was expressed mainly in seed tissues in mature berries.

Carotenoids, which are synthesized mainly during the prevéraison stages of berry development, become degraded following véraison to produce C_13_-norisoprenoids and other non-volatile and non-odorant glycosidic compounds, some of which give rise to odor and flavor compounds during the winemaking process. Enzymatic degradation of carotenoids is carried out by a collection of oxidases that lead to the formation of oxidized carotenoid (xanthophyll) pigments and C_13_-norisoprenoids, which is followed by glycosylation by glycosyltransferases [59]. Xanthophyll cycle enzymes, zeaxanthin epoxidases (TC46508, TC39841) and violoxanthin de-epoxidase (TC47195) (Additional file 3, Table 4), which only appear active in grape berries following véraison, exhibited preferential expression in the skin. Amongst the four Unigenes related to epoxycarotenoids dioxygenase, one (TC44975) corresponds to VvCCD1, whose gene product catalyzes the formation of apocarotenoids from a wide range of carotenoids, and which was preferentially expressed in the skin following véraison and may play a key role in the formation of flavor compounds [10].

### Pathogen-related proteins

Pathogen-related (PR) proteins are the most abundant class of proteins present in wine, and adversely affect the clarity and stability of wine [60,61]. During berry development, PR genes, including thaumatin-like (osmotins) and chitinases, are highly expressed in grape berry tissues as part of normal berry development [19,61-63] and are likely to play important roles in protecting against fungal pathogens and possibly other stresses. Most PR genes, designated as PR 1, 2, 3, 4 5, 6, 10, 14 according to the classification system of Van Loon and Van Strien [64], exhibited differential mRNA expression among various berry tissues (Additional file 3, Table 5). Interestingly, five beta-1–3 glucanase genes were expressed differentially among tissues, although no beta 1–3 glucanase activity was detectable at any stage of development of cv. Shiraz berries [65]. The observed difference could be related to a cultivar-specific activity or to post-translational regulation of beta-1–3 glucanase activity. Three chitinases (TC46138, TC39952, Z54234) were highly expressed in the seed and may be related to embryo formation [66] or fungal defense as observed for class III chitinases [67]. Three other chitinases, which are close homologs to Vvchi4, a gene overexpressed in the pericarp [65], were preferentially expressed in the skin (TC47573, TC38434) or skin and seed (AY137377). Thaumatin encoded by VvTL1 (AF003007), one of the most abundant berry proteins induced at the onset of fruit ripening, was expressed in the pericarp, but not detected in the seed [62]. Surprisingly, mRNA of VvTL1 (AF003007) and another thaumatin gene (AF178653) were expressed specifically in the seed. However, other thaumatin genes showed preferential mRNA expression in the skin and seed (AF532965, TC47844 and TC39447) or pericarp tissues (TC38862). A group of five protease inhibitors (AY156047, TC38119, TC39394, TC39799, TC42241), which are typically expressed in seeds, were expressed specifically in grape seed. However, one protease inhibitor gene (TC38691) was expressed preferentially in pericarp tissues. A group of four Bet v 1 allergen-related protein genes, which are known to be induced by a wide range of signals, such as wounding, pathogen attack, jasmonic acid and oxidative stress, showed a diverse range of tissue-specific expression patterns. Three lipid transfer protein genes (AF467945, TC38438, TC38608), which are thought to function in cuticle formation, in pathogen defense as efficient antifungal agents, and in long-distance systemic resistance signaling [68], were over expressed in the skin. One of these genes (AF467945) was shown previously to undergo increased mRNA expression in response to pathogen inoculation in grape berries, but did not appear to possess antifungal activity [69]. A fourth lipid transfer protein gene (TC46011) was expressed specifically in the pulp.

### Disease resistance and signaling proteins

Lectins are a diverse class of carbohydrate-binding proteins that play a wide variety of roles in cell-cell recognition events that trigger several important cellular processes [70]. Four (TC46148, TC48215, TC46871, TC47689) of the five lectins genes identified as differentially expressed in berries were expressed in the seed, as is typical for many plant lectin genes (Additional file 3, Table 5). However, lectin activity also has been detected in grape juice [71] and could be related to the pericarp-specific mRNA expression of one lectin gene (TC48059). An unknown pathogen-related protein, which is responsive to fungal infection, is expressed specifically in seed (TC38776). Four mRNAs (TC38857, TC42177, CF203567, TC40563), which encode dirigent proteins that catalyze the synthesis of lignan pinoresinols, are localized to sites of lignification within cell walls, and play a role in plant pathogen defense [72], were expressed in one or more tissue types within the berry. Five Avr9/Cf-9-related genes, which encode type I transmembrane glycoproteins carrying extracytoplasmic Leu-rich repeats (LRRs), a single pass membrane-spanning region, and a short cytoplasmic domain that has no similarity to known signaling domains confer resistance to the fungal pathogen (e.g., *Cladosporium fulvum*) through recognition of secreted avirulence (Avr) peptides [73], showed mRNA expression patterns in all three grape berry tissues. Three members of the disease resistance gene family NBS-LRR were over-expressed in pericarp tissues. One member of this gene family (TC40017) contains the TIR domain and could be classified in the TIR/NBS/LRR subclass [74], whereas two others (TC42078 and TC40208) did not contain this domain. Recently, NBS-LRR genes were implicated in having a dual role in both regulating developmental pathways and controlling resistance to fungal pathogens [75].

Two mRNAs (TC50577, TC41602) encoding protein homologs of the barley mildew resistance locus o (MLO) were over-expressed in the skin. Members of this plant-specific seven-transmembrane domain protein family are known to confer durable resistance to powdery mildew in Barley [75] and could be related to the powdery mildew resistance proteins occurring in mature grape berries [76]. Three Unigenes encoding enzymes involved in the metabolism of cyanogenic glucosides were differentially expressed in berry tissues. The gene encoding alpha-hydroxynitrile lyase (TC48696) was over-expressed in the seed, consistent with results from black cherry in which hydroxynitrile lyase activity was restricted to the seed [77]. Surprisingly, a cyanogenic beta-glucoside hydrolase transcript (TC41501) and a mandelonitrile lyase transcript (TC40872) were over-expressed in the skin. Enzymes encoded by theses genes could be related to benzaldehyde formation or glycoconjugated volatile release, but grape berries have not been shown previously to have cyanogenic properties. Another beta-glucosidase transcript (AY039034) related to amygdaline hydrolase [78] was expressed preferentially in the pulp and seed. However, the exact substrate specificity of these enzymes needs to be clarified. Finally, a cell wall or extra cellular beta-fructosidase (invertase) (TC42830) was over expressed in the skin and may function in bacterial pathogen defense by digesting bacterial cell surface slime coats consisting of alginate or levan [79].

### Aroma biosynthesis

In grape berries, volatile aroma compounds, such as terpenes, benzenoids, and phenylpropenoids, accumulate primarily in exocarp and mesocarp tissues [6,7] after the initiation of berry ripening [8-11]. In addition to the genes encoding terpene, phenylpropanoid, and carotenoid metabolism enzymes described above, several other candidate genes have been identified that may have roles in the production of aroma compounds. However, readers should be cautioned that little correlation between the level of sequence similarity and the structural similarity of their substrates has been observed for these protein families [80]. Two isogenes encoding orcinol-O-methyltransferases, which participate in the biosynthesis of the volatile compound 1,3,5-trimethoxybenzene, a compound not previously described in grape, showed differential expression in the seed (CF209780) and pulp (TC40103), respectively (Additional file 3, Table 6). Another enzyme, benzoyl coenzyme A: benzyl alcohol benzoyl transferase, which catalyzes the formation of benzylbenzoate, a volatile aroma compound [81] is encoded by a gene (TC51257), which was preferentially expressed in pericarp tissues. Two other mRNAs encoding members of a methyltransferase gene family, which catalyze the formation of small-molecule methyl esters using S-adenosyl-L-Met (SAM) as a methyl donor and carboxylic acid-bearing substrates as methyl acceptors [82], were differentially expressed in the seed (CA818350) and pericarp (TC39566), respectively. Fourteen cytochrome P450 genes were differentially expressed among various berry tissues, some of which may participate in the oxidative cleavage of fatty acids or hydroxylation of monoterpenes leading to the formation of plant volatiles [80]. Finally, eleven caffeic acid O-methyltransferase genes showed tissue-specific expression patterns (Additional file 3, Table 6), some of which may encode enzymes that catalyze the formation of scent compounds [80].

### Transport facilitation

A large number of genes encoding proteins with functions in the transport of water, ions, sugars, and non-specific substrates (ABC transporters) showed differential expression within berry tissues. Water channels or aquaporins are part of the major intrinsic protein (MIP), plasma membrane (PIP) or tonoplast intrinsic protein (TIP) protein family that facilitate the transport of water and other solutes through membranes. Two aquaporins (VvPIP2-1, TC38121; VvPIP2-2, TC38281) isolated previously from leaves of *Vitis *hybrid Richter-110 [83] were expressed primarily in pericarp tissues (Additional file 3, Table 7). Two other PIPs (PIP1a, AF188843; TC38445) and a TIP (TC38576) were expressed specifically in the skin. Another TIP (TC39811) was over-expressed in the seed and may be involved in metabolite transport in this structure [84].

Calcium is a ubiquitous second messenger, with the location and frequency of transient changes in cytosolic Ca^2+ ^concentrations ([Ca ^2+ ^]_cyt_) playing a central role in signal transduction in plant cells [85]. Maintenance of low Ca^2+ ^electrochemical activity in the Ca^2+^-responsive compartment is achieved by Ca^2+^-transporting ATPases, Ca^2+^/H^+ ^antiporters and K^+^-dependant Na^+^/Ca^2+ ^exchangers [85,86]. Five Ca^2+^-transporting ATPase isogenes were differentially expressed in various berry tissues (Additional file 3, Table 7). A Ca^2+^/H^+^-antiporter (TC46112) and a two-pore Ca^2+^-channel (TC48242) were expressed primarily in seeds, whereas a K^+^-dependant Na^+^/Ca^2+ ^exchanger was expressed specifically in the skin (TC39122).

Grapevine varieties are ranked moderately sensitive to salinity stress, with salt sensitivity resulting mainly from Cl^- ^rather than Na^+ ^toxicity[87]. Although no chloride transporters were identified as being differentially expressed in the current analysis, two Na^+^/H^+^ antiporters were differentially expressed in the pericarp (TC48358) and seed (TC41399), respectively. In plant cells, the maintenance of low cytoplasmic Na^+ ^and Cl^- ^concentrations and a high K^+^/Na^+ ^ratio is essential to cellular viability because K^+ ^counteracts the inhibitory effects of Na^+ ^(and Li^+^). Most plant cells maintain cytosolic K^+ ^concentrations in the range of 100–200 mM and Na^+ ^values in the low mM range (1–10 mM) up to a maximum of 100 mM [88]. Four of five K^+ ^transporters or channels were expressed primarily in the skin (TC49359, TC41344) or pulp (TC47746, AJ490336), whereas one was expressed primarily in the seed (TC47109). TC49359 and AJ490336 were most closely related to KT and SKOR potassium channels, respectively, whereas TC41344, TC47746 and TC47109 were most closely related to KUP/HAK/KT potassium transporters [89].

Three sulfate transporters, which are members of group 3 type sulfate transporters whose expression is not regulated by sulfate in *Arabidopsis *and *Brassica oleracea *[90], were expressed preferentially in the seed (TC47443, TC49555) and pulp (TC48754).

Two phosphate transporters were expressed specifically in the skin (TC42012, TC40312). These Unigenes are homologous to putative Na^+^/Pi symporters, however, this type of transport activity has not yet been demonstrated in vascular plants [91]. A third inorganic phosphate transporter (TC47178) was over-expressed in seed.

During berry development malate accumulation in the vacuole peaks at véraison and then declines, however, vacuolar ATPase (V-ATPase) and pyrophosphatase (V-PPase) activities and protein abundance increase following véraison [92]. A sodium-dicarboxylate cotransporter (TC41552), which is involved in the transport of malate in the vacuole [93], was expressed primarily in the seed. The pericarp-specific V-PPase (TC45835) encodes a key enzyme responsible for the energization of malate transport into the vacuole [92]. However, no V-ATPase genes were found to be expressed differentially in berry tissues.

ATP-binding cassette (ABC) transporters are the largest known protein family and are mostly membrane-localized proteins that transport a broad range of substance across membranes. The 12 differentially expressed *Vitis *Unigenes were compared with the *Arabidopsis *ABC transporter family and grouped into known subfamilies [94]. Four (TC42025, TC43702, TC48111, TC48713) of the six Unigenes within the white-brown complex (WBC) subfamily were expressed in the skin. Four of them that are closely related to the ghWBC1 gene were over-expressed in the skin, whereas two others were expressed in the seed (TC49764) and pulp (TC40406), respectively. The TC47027 Unigene, which was preferentially expressed in skin and seed, is a close homolog to the AtMDR1 gene. This gene is known to modulate auxin transport and seedlings from a loss-of-function *Arabidopsis *mutant that exhibited low flavonoid accumulation [95]. Several other ABC transporter family genes showed differential tissue expression patterns, although the function of these family members remains obscure (Additional file 3, Table 7).

One of the main features of grape berry development is the accumulation of hexose sugars (i.e., Glc and Fru), which begins at véraison and continues throughout the ripening process. Sucrose derived from photosynthesis is transported to the berry via the phloem where it is cleaved to Glc and Fru, which accumulate in roughly equal amounts. Four hexoses transporters showed mRNA expression primarily in the pericarp (Additional file 3, Table 7). Two of these genes (TC41130, VvHT2; TC39483, VvHT6) exhibit increased expression during berry development [21]. A third gene (TC44413) shares 98% amino acid sequence identity with vvHT6. The function of a fourth putative sugar transporter (TC49698), which is annotated as an organic cation transporter and contains sequence similarity to other sugar transporters, will require experimental evidence to validate its function. Four other hexose transporters were expressed primarily in the seed, however, two of these also showed mRNA expression in the skin (TC47882, vvHT7) or pulp (TC40715). One of these genes (AF021810, VvSUC27) was shown previously to be expressed in the seed [96]. Enzymes associated with sucrose transport such as sucrose phosphate synthase (TC44139) are expressed preferentially in the pericarp and probably participate in the resynthesis of sucrose following its unloading into the fruit from the phloem [97].

### Hormone biosynthesis and regulation

A number of plant growth regulators including abscisic acid (ABA), auxin (indole-3-acetic acid [IAA]), ethylene, gibberellic acid, and brassinosteroids have been implicated in the control of berry development and ripening. However, the tissue-specificity of plant growth regulator signaling, biosynthetic, and target genes remains poorly understood. Auxin concentrations peak during flowering and early berry developmental stages and then decline to very low levels after véraison. Application of the synthetic auxin-like compound benzothiazole-2-oxyactetic acid (BTOA) delays both an increase in endogenous ABA content and ripening in berries as well as the accumulation of transcripts of ripening-associated genes [98]. A variety of auxin-induced or auxin-responsive proteins and proteins with putative auxin transport functions were preferentially expressed in the pulp and skin (Additional file 3, Table 8). However, a few auxin-induced/repressed proteins and carriers showed largely seed-specific expression. A nitrilase homolog (TC40938), which likely plays a role in auxin biosynthesis by catalyzing the conversion of indol-3-acetonitrile to IAA [99], was expressed in the skin and seed. Overall, these results suggest that most auxin signaling occurs in the berry pericarp.

ABA amounts in berries decrease after flowering, but then increase dramatically following véraison [98]. TC43255, TC42536 and TC48377, which encode nine-cis-epoxycarotenoid dioxygenases (NCED) [100], which catalyzes the key regulatory step in abscisic acid (ABA) biosynthesis, exhibited preferential expression in pulp tissue. ABA synthesis in the pulp may be important for embryo and endosperm formation during seed development, seed dormancy in mature berries, and for the promotion of fruit ripening. Several ABA regulatory factors, such as Abscisic acid insensitive 3/Viviparous 1 (ABI3/VP1)-related transcription factors (TC50572 and TC40343), which play central roles in the ABA-dependent establishment of desiccation tolerance and dormancy during zygotic embryogenesis [101], were expressed mainly in the seed. ABA- and dehydration-responsive genes that encode embryo-specific proteins (CB982875, RD22 BURP-domain containing proteins), and Em, a Group 1 late embryogenesis abundant (LEA) protein (TC41158), were expressed in the seed (Additional file 3, Table 9). Transcripts for proteins implicated in ABA-signaling of stress response pathways, such as calcium-dependent protein kinase (TC41302, AtCDPK1) [102], ACR proteins (TC41345, ACR8) [103], and serine/threonine protein kinases (TC49237, TC50832), were expressed in the pulp or skin. Other ABA- and dehydration-responsive transcripts, such as HVA22a (CB982969) and RD22 (TC38296, TC38295, and TC38630), were also expressed primarily in the pulp or skin.

Very little is known about the role of gibberellins in the development and maturation of grape berries except that they are thought to contribute to cell enlargement. Most Unigenes related to gibberellin (GA) biosynthesis and response exhibited very low mRNA expression in all tissues and only a few Unigenes were expressed differentially (Additional file 3, Table 10). This is not surprising as GA plays a role mostly during flowering and fruit set [104]. However, the GA-stimulated transcript 1 (GAST1) gene (TC38517), which is inhibited by ABA and induced by GA, was over-expressed in the seed. GA-insensitive (GAI1) genes encoding transcriptional repressors of GA responses (AY256862 and AF378125) were also expressed mainly in seed. Two different gibberellin 2-oxidase genes (TC42201 and TC47765) involved in GA biosynthesis showed differential expression in the skin and seed, respectively.

Methyl jasmonate and jasmonic acid (JA) is known to promote the synthesis and accumulation of terpenes in leaves and resveratrol in berry cell cultures of grape [105,106]. Three Unigenes involved in JA signaling all exhibited preferential expression in the pericarp: a) CORONATINE INSENSITIVE 1 (COI1) (TC38851), encoding a F-box-motif-containing protein involved in protein ubiquitination and degradation regulated by JAs and a central component of JA signaling pathways [107]; b) a mitogen-activated protein kinase 4 (TC46783) involved in the crosstalk between JA and ethylene [108]; and c) a carboxypeptidase (TC40697) involved in wound responses through JA signaling [109] (Additional file 3, Table 11). In contrast, mRNAs involved in the biosynthesis of JA showed differential mRNA expression patterns among the various tissues. For example, lipoxygenase/lipoxygenase (LOX) D genes (TC43235, TC44915, and CF405309), which belong to a family of JA-induced LOXs, which catalyze the regioselective and stereoselective dioxygenation of 1,4-pentadiene cis-polyunsaturated fatty acids into their corresponding hydroperoxy derivatives, and allene oxide synthase (TC49699), a cytochrome P-450 (CYP74A), which catalyzes the first step in the conversion of 13-hydroperoxy linolenic acid to jasmonic acid, were over expressed in the pericarp. In contrast, 12-oxophytodienoate reductase (OPR2, TC41392), which catalyze the reduction of double bonds adjacent to an oxo group in alpha, beta-unsaturated aldehydes or ketones, and 3-keto-acyl-CoA thiolase (TC46796), were expressed in the seed. Lack of expression in the pericarp suggests that the enzymes encoded by these genes are not likely to participate in the octadecanoid pathway that converts linolenic acid to JA. JA is known to induce the expression of a broad range of disease resistance related genes. Nine mRNAs presented pericarp-specific expression patterns similar to JA-regulated genes, although the distribution of JA amongst berry tissues is not known. JA is known to trigger increased phenylpropanoid abundance, however, as none of the JA-related genes were found to be differentially expressed between pulp and skin, conclusions about the involvement of JA in phenylpropanoids pathway cannot be drawn at the present time.

Although grape is considered a non-climacteric fruit, recent studies suggest that ethylene plays a critical role in grape berry development and ripening and is required for increased berry diameter, decreased berry acidity and anthocyanin accumulation in ripening berries [110]. Most mRNAs encoding genes involved in ethylene biosynthesis (S-adenosyl-L-methionine (SAM) synthetase 1 and 2 (TC38334 and TC38332); 1-aminocyclopropane-1-carboxylate (ACC) oxidase (TC45908), were preferentially expressed in the skin (Additional file 3, Table 12). Other components that control ethylene signaling and regulatory pathways, such as the ethylene receptor (TC50911), an ACC synthase repressor (ETO1, TC40150), and an EIN3 repressor (TC39880), a positive regulatory of the ethylene response, and an EREBP co-activator (TC45815), were expressed in the pericarp. Furthermore, the under-expression of the negative regulator CTR1 (TC48190) in these tissues suggests ethylene may be produced predominantly in the pericarp. A variety of other genes, identified putatively as ethylene response factors (ERFs) and the ethylene responsive proteins, exhibited diverse tissue-specific expression patterns, suggesting that their expression may also be controlled by other factors.

### Transcriptional regulation

A large number of transcription factors display diverse patterns of tissue-specific expression within berry tissues (Additional file 3, Table 13). Several Myb transcription factors, MybA2 (AB073013) and MybC (AB073014) have been characterized previously in grape [111]. The seed-specific expression of MybC was confirmed along with that of other Myb genes (TC39707, TC46393, TC46978). MybA2, which is responsible for the control of anthocyanin biosynthesis via the expression of UFGT, showed a skin-specific pattern of expression, rather than a pericarp-specific pattern of expression as was observed previously [111]. This dissimilarity in tissue-specificity could be attributed to species differences (*V. labrusca *vs. *V. vinifera*) or the hybridization detection method (Northern blot vs. microarray) employed. Several other Myb transcription factor genes also showed skin-specific expression patterns (TC48484, TC45624, and TC51437), whereas others were expressed preferentially in the pulp (TC48806, TC41451). The homolog of Myb factor (TC40617), which in *Antirrhinum *functions in petal development [112], is expressed mainly in the pericarp along with several other Myb genes (TC45686, TC49276) and a gene encoding a Myc anthocyanin regulatory protein (TC48362).

The WRKY transcription factor super family is involved in a diverse set of biological functions including pathogen defense, abiotic stress responses and plant development and displays diverse expression patterns within tissues [113]. The weak sequence homologies outside the zinc-finger and WRKY domains with other plant genes make it difficult to establish a concrete hypothesis about the functions of their grape homologs other than to note that most showed mRNA expression in the skin.

### Tissue-specific mRNA expression of water-deficit responsive genes

**Figure 4:**
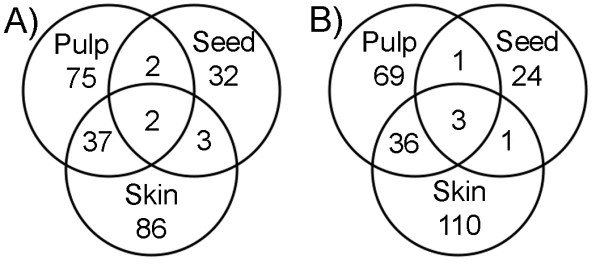
**Venn diagrams of differentially expressed mRNAs under water-deficit**. A) Unigenes over-expressed two-fold or greater among tissues under the well-watered condition. B) Unigenes over-expressed two-fold or greater among tissues under the water-deficit condition.

Water-deficit stress resulted in 699, 596, and 201 genes with statistically significant differential expression (p < 0.05) in pulp, skin, and seed, respectively. We next identified 237 (248 probesets) and 244 Unigenes (254 probesets) from berries harvested from well-watered or water-deficit-treated vines, respectively, which were differentially expressed (p < 0.05) amongst the three different berry tissues with a two-fold or greater change in mRNA abundance (Figure 4).

In berries from well-watered vines, 116, 39, and 128 transcripts were significantly over-represented in the pulp, seed, and skin, respectively (Figure 4A). A large number of genes were expressed in both pulp and skin (39), whereas very few genes displayed shared over-expression between the pulp and seed (4) or the skin and (5). Only two genes, TC41864 (Auxin response family protein) and TC40435 (Aldose 1-epimerase), showed over-expression in all three well-watered berry tissues. In berries from water-deficit treated vines, 109, 29, and 150 transcripts were significantly over-expressed in the pulp, seed, and skin, respectively (Figure 4B). As in well-watered berries, a large proportion of genes were expressed in both the pulp and skin (39) of berries from water-deficit treated vines, whereas very few genes displayed co-overexpression between the pulp and seed (4) or the skin and seed (4). Only three genes (AY037918, TC46456, TC41805), all of which are unknown or novel, showed over-expression in all three water-deficit stressed berry tissues.

### Functional categorization of differentially expressed genes among berry tissues in response to water-deficit stress treatment

Functional categories were assigned to the Unigenes that showed tissue-specific, differential expression in response to well-watered or water-deficit treatments according to the clusters defined in Figure 4. Pulp and skin tissues showed greater differential gene expression changes in response to water-deficit stress compared to seed tissue amongst different functional categories (Table 3). The largest number of instances of significantly increased transcript abundance in the pulp and the skin occurred within the metabolism (carbohydrate and secondary metabolism) and cell rescue, defense, and virulence (biotic stress response functions) categories. Notably, transcripts encoding transport facilitation-related functions were over-represented in the pulp of berries from well-watered vines, whereas those encoding energy-related functions were over-represented in the seeds of berries from well-watered vines. Some categories seem to participate little in water status related changes: proteins with binding function, protein fate (in well-watered pulp) and protein synthesis (in water-deficit skin). To gain a better understanding of the detailed functional roles of the genes that were differentially expressed across the three different berry tissues with a two-fold or greater change in mRNA abundance (Figure 4), we organized these genes into a series of functional categories (Additional file 4, Tables 14–21). A complete listing of all differentially expressed genes is presented (Additional Files 5, Tables 22–23).

**Table 3:** Functional categories of the differentially expressed (p ≤ 0.05, 2-fold) mRNA between pulp and skin from well-watered and water-deficit treated vines.

**Functional category**	**% Unigenes over expressed in pulp:**		**% Unigenes over expressed in skin:**		**% Unigenes over expressed in seed:**		**% total in probesets**
	
a) Top level	Well-watered	Water-deficit	Well-watered	Water-deficit	Well-watered	Water-deficit	
	
Protein with binding function	*19.8 (23)*	*11.0 (12)*	*10.2 (13)*	*11.9 (18)*	*17.5 (7)*	10.3 (3)	29.5 (5994)
Metabolism	**37.9 (44)**	**28.4 (31)**	23.4 (30)	**41.7 (63)**	42.5 (17)	6.9 (2)	20.9 (2851)
Protein fate	*8.6 (10)*	6.4 (7)	8.6 (11)	*5.3 (8)*	15 (6)	0 (0)	16.7 (2270)
Signal transduction mechanism	12.9 (15)	9.2 (10)	8.6 (11)	9.9 (15)	10 (4)	6.9 (2)	13.6 (1847)
Transcription	7.8 (9)	10.1 (11)	11.7 (15)	7.3 (11)	5 (2)	6.9 (2)	12.9 (1762)
Cell rescue, defense and virulence	**21.5 (25)**	**18.3 (20)**	11.7 (15)	**18.5 (28)**	22.5 (9)	10.3 (3)	11.1 (1509)
Development	12.9 (15)	7.3 (8)	10.9 (14)	*3.3 (5)*	12.5 (5)	3.4 (1)	11.8 (1611)
Cell fate	6.0 (7)	5.5 (6)	5.5 (7)	4.6 (7)	5 (2)	0 (0)	11.7 (1593)
Control of cellular organization	6.9 (8)	6.4 (7)	9.4 (12)	10.6 (16)	10 (4)	10.3 (3)	9.5 (1296)
Interaction with cellular environment	12.1 (14)	3.7 (4)	14.1 (18)	4.6 (7)	10 (4)	6.9 (2)	8.5 (1158)
Cell cycle and DNA processing	3.4 (4)	1.8 (2)	*1.6 (2)*	3.3 (5)	0 (0)	0 (0)	8.4 (1145)
Transport facilitation	19.8 (23)	8.3 (9)	13.3 (17)	7.9 (12)	12.5 (5)	3.4 (1)	8.6 (1174)
Cellular transport	8.6 (10)	1.8 (2)	4.7 (6)	4.0 (6)	0 (0)	0 (0)	7.2 (982)
Protein synthesis	*1.7 (2)*	2.7 (3)	2.3 (3)	4.0 (6)	10 (4)	0 (0)	7.5 (1017)
Energy	14.7 (17)	9.2 (10)	10.2 (13)	10.6 (16)	**22.5 (9)**	0 (0)	7.0 (947)
Interaction with environment	12.1 (14)	9.2 (10)	7.8 (10)	9.9 (15)	5 (2)	0 (0)	7.0 (956)
Protein activity regulation	1.7 (2)	0 (0)	3.1 (4)	0.7 (1)	2.5 (1)	0 (0)	3.5 (478)
Cell type differentiation	1.7 (2)	0 (0)	2.3(3)	0 (0)	0 (0)	0 (0)	1.9 (257)
Storage protein	0 (0)	0.9 (1)	0 (0)	0 (0)	0 (0)	6.9 (2)	0.3 (45)
Unknown function	13.8 (16)	5.5 (6)	14.8 (19)	13.9 (21)	2.5 (1)	10.3 (3)	9.6 (1306)
No hit	14.7 (17)	38.5 (42)	22.7 (29)	23.2 (35)	30 (12)	58.6 (17)	34.2 (4656)
b) Metabolism							
Carbohydrates	**19.8 (23)**	11.9 (13)	**13.3 (17)**	**17.9 (27)**	**17.5 (7)**	6.9 (2)	8.6 (1169)
Secondary metabolism	**13.8 (16)**	**12.8 (14)**	**9.4 (12)**	**22.5 (34)**	**15 (6)**	0 (0)	4.8 (652)
Lipids	10.3 (12)	7.3 (8)	3.9 (5)	6.0 (9)	10 (4)	0 (0)	4.6 (632)
Amino acids	6.9 (8)	3.7 (4)	5.5 (7)	7.3 (11)	7.5 (3)	0 (0)	3.8 (511)
Vitamins and cofactors	5.2 (6)	2.7 (3)	2.3 (3)	4.6 (7)	5 (2)	0 (0)	2.4 (326)
Phosphate	0.9 (1)	1.8 (2)	0 (0)	0 (0)	2.5 (1)	0 (0)	3.2 (433)
Nucleotides	4.3 (3)	2.7 (3)	3.9 (3)	3.3 (4)	7.5 (3)	0 (0)	2.9 (401)
Nitrogen and sulfur	4.6 (5)	2.6 (3)	3.3 (5)	3.9 (5)	7.5 (3)	0 (0)	1.4 (184)

Unigenes were grouped according to Figure 1. The functional categories were assigned according to the MIPS functional catalog as a) top level categories and b) second level categories within the metabolism category. The values in parentheses correspond to the total number of Unigenes observed in each category. The last column corresponds to the relative percentage within each category of all 13,622 Unigenes present on the *Vitis *Genechip^® ^genome array. Typefaces indicate functional categories that are significantly over- (bold) or under- (italic) represented when compared to the overall distribution of all 13,622 Unigenes using a Fisher's exact test. Underscores indicate over- or under-representation, respectively, of more than 1.5-fold the percentage expected in that category based on the overall distribution of all *Vitis *Unigenes.

### Phenylpropanoid pathway

Water-deficit stress had an overall profound effect on the mRNA expression of many genes within the general phenylpropanoid pathway. Enzymes for the first three steps of the phenylpropanoid pathway and stilbene synthase each were encoded by at least one Unigene that showed water-deficit-stress enhanced mRNA accumulation specifically in the skin (Figure 3; Additional file 4, Table 14) and may drive the increased accumulation of skin-derived anthocyanins observed following water-deficit stress in cv. Cabernet Sauvignon [114]. The increased mRNA abundance of stilbene synthase (AF418567) is similar to that observed in berries under water-deficit stress brought about by drying, UV-light and/or heat exposure and is expected to result in increased accumulation of resveratrol [115] (Additional file 4, Table 14). Chalcone synthase (X75969) mRNA abundance is elevated slightly by water-deficit stress in the skin. Eight mRNAs involved in lignin biosynthesis were over expressed in the water-deficit skin tissue with cinnamoyl CoA-reductase Unigenes being expressed in the pulp (TC39574) and skin (TC44947), respectively (Figure 3; Additional file 4, Table 14). In contrast to genes in the general phenylpropanoid and lignin biosynthetic pathways, many flavonoid biosynthetic genes showed reduced transcript abundance in response to water-deficit stress, with the exception of flavonol synthase (TC46143). Although no change of the expression of genes catalyzing intermediate steps within the flavonoid and proanthocyanidin pathways were observed, leucoanthocyanin reductase (TC39641) displayed increased mRNA abundance in response to water-deficit in the skin (Figure 3; Additional file 4, Table 14). This Unigene may contribute to the increased accumulation and polymerization of tannins within the skin that is known to occur in response to water-deficit [114,116]. Only one Unigene involved in anthocyanidin biosynthesis, UTP-glucose glucosyltransferase (TC38177), exhibited increased mRNA abundance in response to water-deficit in the skin. With the exception of a single chalcone synthase Unigene (X75969), no water-deficit responsive mRNA of the phenylpropanoid pathway was expressed differentially in the seed. Interestingly, a Myb transcription factor (VvMYBA2, TC48484), which showed a skin-specific expression pattern under well-watered conditions, exhibited a largely pulp-specific expression pattern under water-deficit treatment.

### Cell wall metabolism

Water deficit reduced berry size significantly (Table 1). This reduction in size correlated well with the reduction in mRNA abundance for genes encoding many cell wall metabolism enzymes including two polygalacturonase genes (TC47230, TC49646), a pectinmethylesterase gene (TC47054), and two expansins (TC38813, TC46110) (Additional file 4, Table 15). Inhibition of cell expansion can result from reduced cellular turgor as well as biochemical changes that affect cell expansion through processes that control wall extensibility. Microarray studies of shoot tissues of *Vitis *vines exposed to water-deficit stress also showed a decline in transcript abundance for cell wall metabolism genes [24], and similar processes appear to occur in berries.

### Transport facilitation

The transport of water and ions are also important determinants of berry size. Two aquaporin Unigenes displayed significantly decreased mRNA abundance either in both pulp and skin (TC45189) or in skin (TC38576) under water-deficit stress conditions and seem directly affected by the decrease of water flux (Additional file 4, Table 16). The TC45189 Unigene is closely related to the TIP3 homolog described previously in the Vitis hybrid Richter-110, whose mRNA expression was barely detected in different vegetative tissues [83]. A potassium transporter (TC48913) showed decreased abundance following water-deficit stress. The expression level of a homologous *M. crystallinum *gene increased in response to salt stress, suggesting its involvement in the regulation of cellular osmotic potential [117]. In contrast, a second potassium transporter-like protein (TC47109) showed increased mRNA abundance in the skin following water-deficit treatment.

### Sugar metabolism

Water-deficit increased Brix in berries (Table 1) consistent with previous observations on the effects of water-deficit of cv. Cabernet Sauvignon [114]. mRNA abundance of a pulp- and seed-specific sugar transporter (TC40715) and a pulp-specific vacuolar invertase (TC45440) decreased following water-deficit, suggesting that the difference of Brix may be related to the decrease of berry size rather than to an increase in hexose unloading into the berry (Additional file 4, Table 17). However, it remains possible that sugar content is modified as a result of water-deficit stress induction of other classes of sugars and sugar alcohols. In support of this hypothesis, mRNA abundance of Unigenes encoding a sorbitol transporter (TC38597) in the skin, and a sorbitol dehydrogenase (TC45858) in the pulp/skin, increased in response to water deficit stress, which could account for alterations in the sorbitol/sucrose ratio of the berry. Transcript abundance for UDP-galactose 4-epimerase (CF515277), which is responsible for the biosynthesis of UDP-Galactose, a precursor for the biosynthesis of numerous different carbohydrates including trehalose, also increased in berries in response to water deficit.

### Hormone biosynthesis and signaling

Water-deficit had a profound influence on the mRNA abundance of Unigenes encoding ethylene biosynthesis and response functions (Additional file 4, Table 18) suggesting that ethylene plays a critical role in grape berry development and ripening under water-deficit stress conditions. S-adenosyl-L-methionine (SAM) synthetase 1 (TC38334) and a water-deficit stress-inducible SAM synthetase Unigene (TC45963) were expressed preferentially in skin following water-deficit stress. 1-aminocyclopropane-1-carboxylate (ACC) oxidase (TC45908) and a water-deficit stress-inducible ACC oxidase Unigene (CF201799) were expressed preferentially in both pulp and skin (Additional file 4, Table 18). Two ethylene response factors (ERFs; TC47273 and TC41585) showed water-deficit-inducible mRNA expression primarily in the pulp.

Auxin concentrations are known to decline to very low levels after véraison [98] and are expected to decline in response to water-deficit. Consistent with this hypothesis was the decline in mRNA abundance for many auxin-induced or auxin-responsive proteins, with the exception of one auxin-induced protein (TC40686) and a PIN1-like auxin transport protein, which showed increased mRNA expression following water-deficit stress (Additional file 4, Table 18).

ABA amounts in berries decrease after flowering, but then increase dramatically following véraison [98] and are expected to increase following water-deficit stress. This predicted increase is likely mediated by the increased mRNA expression of only a single nine-cis-epoxycarotenoid dioxygenases (NCED) Unigene (TC48377), which exhibited mRNA expression in all tissues with preferential expression in the skin. ABA- and dehydration-responsive genes RD22 (TC38295, and TC38630) showed reduced mRNA abundance following water-deficit stress, whereas LTCOR11 (TC38517) mRNA abundance increased in skin and pulp. Few ABA-responsive genes were noted in seed tissues as this tissue is expected to have essentially completed its development at this late stage of berry maturation.

Messenger RNAs involved in the biosynthesis of JA showed differential mRNA expression patterns in response to water-deficit stress, with some lipoxygenase Unigenes (TC44915 and TC43235) being reduced, whereas another showed increased mRNA expression (CF405309). 12-oxophytodienoate reductase (OPR2, TC41392) mRNA expression was reduced in the seed, whereas allene oxide synthase (TC49699) was increased significantly in the skin.

### Disease resistance and signaling proteins

Whereas some pathogenesis-related proteins, chitinases, non-specific lipid transfer proteins, an allergen protein, and a gene encoding alpha-hydroxynitrile lyase (TC48696) were repressed in their mRNA expression following water-deficit treatment, a larger number of pathogenesis-related proteins exhibited increased mRNA expression (Additional file 4, Table 19). Specifically, multiple Unigenes encoding beta-1,3-glucanases, chitinases, thaumatin, thaumatin-like proteins, an Avr9/Cf-9-rapidly elicited protein, and disease resistance proteins were induced. The induction of this large number of PR genes is consistent with cross-talk between biotic and abiotic stress response pathways and suggests that water-deficit stress may result in improved resistance to microbial pathogens, although additional testing is needed to confirm this possibility.

### Aroma biosynthesis

Unigenes encoding enzymes likely to participate in the production of aroma compounds display both significantly decreased and increased mRNA abundance following water-deficit stress. Biosynthetic enzymes responsible for a number of volatile compounds showed significant reductions in mRNA following water-deficit stress including key enzymes involved in IPP biosynthesis via the non-mevalonate pathway such as 1-deoxy-D-xylulose 5-phosphate synthase (CF206716), (E)-beta-ocimene synthase (TC40259), which catalyzes the synthesis of a monoterpene olefin derived from geranyl diphosphate, a component of many floral scents, and zeaxanthin epoxidase (TC39841), a xanthophyll cycle enzyme, which results in the formation of oxidized carotenoids. In contrast, a S-adenosyl-L-Met (SAM) methyltransferase gene, which was expressed predominately in the seed (CA818350) under well-watered conditions, showed significant expression in the skin under water-deficit conditions. The mRNA abundance of beta-carotene hydroxylase (TC42069), which catalyzes the hydroxylation of the beta-rings of alpha- and beta-carotene and is required for synthesis of essentially all xanthophyll pigments, was also induced significantly in the pulp following water-deficit stress. Five caffeic acid O-methyltransferase genes showed increased mRNA abundance in water-deficit treated berries, mainly in the pulp and skin (Additional file 4, Table 20). Some of these may encode enzymes that catalyze the formation of scent compounds [80]. Finally, two cytochrome P450s (CF206021 and TC38220), which may participate in the oxidative cleavage of fatty acids or hydroxylation of monoterpenes leading to the formation of plant volatiles [80], were induced significantly in skin and pulp tissues. These findings suggest that the abundance of some volatile compounds will decline whereas the abundance of others will increase following water-deficit stress.

### Transcription factors

The abundance of mRNA for a number of transcription factors was influenced by water deficit with the vast majority exhibiting significant changes in either pulp or skin tissues (Additional file 4, Table 21). Within the WRKY transcription factor super family, only one gene showed reduced expression in response to water-deficit (BQ800205), whereas four WRKY genes showed increased mRNA abundance in pulp and/or skin tissue. The water-deficit stress-induced expression pattern of these family members is consistent with the proposed role of many members of this family being involved in pathogen defense and abiotic stress responses [113]. However, additional functional testing will be required to assess their function in grape berries. Two NAM (no apical meristem)-like transcription factors (TC43527 and TC42530) also exhibited dramatically increased mRNA abundance following water-deficit treatment. Specific members of this large family of plant-specific transcription factors have been shown to be involved in pathogen attack, wounding, and environmental stress responses [118].

## Conclusion

These results demonstrated that approximately 60% of all genes whose mRNA abundance could be monitored in grape berries exhibited significant differential expression among skin, pulp, or seed tissues with more than 28% of genes showing pronounced (2-fold or greater) differences in mRNA expression patterns in a particular tissue. The largest difference in tissue-specific expression was observed in the seed as compared to the pulp or skin. Each tissue displayed a unique repertoire of gene expression reflecting its functional role within the berry. The skin preferentially expressed genes with functions associated with pathogen resistance, flavonoid biosynthesis production, and cell wall modification, whereas pulp tissues preferentially expressed genes involved in cell wall function and transport processes. In contrast, seeds mainly expressed genes encoding phenylpropanoid biosynthetic enzymes, seed storage and embryo proteins. Water deficit had a profound effect on mRNA expression patterns affecting the mRNA abundance of 13% of genes with most of these changes being observed within the pulp and skin. In the skin and pulp, water-deficit significantly increased transcript abundance from several functional categories including those associated with the biosynthesis of phenylpropanoids, lignin, proanthocyanins as well as aroma metabolites. Such changes in gene expression may have a direct impact on the quality of wine produced from such berries. The extensive catalog of gene expression patterns defined here will serve as an invaluable reference for future investigations including the exploration of the complex transcriptional regulatory hierarchies that govern tissue-specific development and differentiation within berries.

## Methods

### Plant material

*Vitis vinifera *L. cv. Cabernet Sauvignon berry clusters were harvested on September 22, 2004 at developmental stage 38 [28] from 20-year-old vines in the Shenandoah Vineyard (Amador County, California) from vines grown on a quadralateral trellis systems and stored on ice for 5 hours. Pulp, skin and seeds were separated and frozen immediately in liquid nitrogen and stored at -80°C. One of the replicates for the well-watered seed was harvested a week before this vintage date. For the well-watered plants, irrigation was performed from stage 27 [28]. Water-deficit treated vines were never irrigated. Two berry clusters were harvested and combined for each treatment: one cluster from the sunny (Southern) side of the vine and one cluster from the shadier (Northern) side of the vine to minimize mRNA differences brought about by sun exposure.

### Stem water potential

Fully mature leaves were selected for stem water potential measurements [119]. A single leaf per plant was tightly zipped in a plastic bag to eliminate transpiration. Aluminum foil was then placed around the bag, deflecting light and heat. After two hours of equilibration time, the excised leaf was placed in a pressure chamber (3005 Plant Water Status Console, Soilmoisture Equipment Corp., Santa Barbara, CA, USA). The foil was removed before sealing the bagged leaf in the chamber. The balancing pressure required to visibly push stem xylem sap to the cut surface was recorded.

### Brix assay and berry size measurement

The Brix (total soluble solids) was assayed from juice crushed from harvested berries with a refractometer (BRIX30, Leica, Bannockburn, IL). Berry diameter was measured with a micrometer.

### RNA extraction and microarray hybridization

RNA were extracted with a modified Tris-LiCl method [120]. RNA was purified further using Qiagen RNeasy columns (Qiagen Inc., Valencia, CA). Messenger RNA was converted to cDNA using oligo dT primer containing a T7 RNA polymerase promoter sequence and reverse transcriptase. Biotinylated complementary RNAs (cRNAs) were synthesized *in vitro *using T7 RNA polymerase in the presence of biotin-labeled UTP/CTP, purified, fragmented and hybridized in the GeneChip^® ^*Vitis vinifera *(Grape) Genome Array ver. 1.0 cartridge (Affymetrix^®^, Santa Clara, CA). The hybridized arrays were washed and stained with Streptavidin Phycoerythrin and biotinylated anti-streptavidin antibody using an Affymetix Fluidics Station 400. Microarrays were scanned using a Hewlett-Packard GeneArray^® ^Scanner and image data were collected and processed on a GeneChip^® ^workstation using Affymetrix^® ^GCOS software.

### Microarray data quality control, processing, and analysis

GeneChip^® ^*Vitis vinifera *(Grape) Genome Arrays ver. 1.0 (Affymetrix^® ^Inc., Santa Clara, CA) were first inspected using a series of quality control steps. As recommended by the GeneChip^® ^Operating Software Users Guide, the levels of average background and noise (RawQ) were examined for consistency across all eighteen arrays. Average background values ranged from 28 to 68 when run on 10% PMT scanner settings, with mean value 43 and standard deviation 12. Noise levels, as given by the RawQ values, fell between 0.7 and 2.3, with a mean value of 1.3, and standard deviation of 0.4. The Present call rates were also consistent across the eighteen arrays, ranging from 57% to 69% (mean rate was 64%). All of the hybridization controls BioB, BioC, BioC and Cre were found to be present 100% of the time. Additionally, it was verified that signal intensities of BioC, BioD, and Cre increased, respectively. Lastly, 3' to 5' ratios of both actin and GAPDH were checked: the actin ratios were always less than 1.1; GAPDH ratios were always less than 1.2. Images of all arrays were examined, and no obvious scratches or spatial variation was observed. A visual inspection of the distributions of raw PM probe values for the eighteen arrays showed no outlying arrays. Similarly, digestion curves describing trends in RNA degradation between the 5' end and the 3' end of each probeset were generated, and all eighteen proved very consistent. Raw microarray data from this experiment are publicly available at the Plant Gene Expression database (PLEXdb) [121] and labeled "VV3: Grape berry tissues differentiation".

Raw intensity values were processed first by Robust Multi-Array Average (RMA) [30] using the R package affy [122]. Specifically, expression values were computed from raw *CEL *files by first applying the RMA model of probe-specific correction of PM (perfect match) probes. These corrected probe values were then normalized via quantile normalization, and a median polish was applied to compute one expression measure from all probe values. Resulting RMA expression values were log_2_-transformed. Distributions of expression values processed via RMA of all arrays were very similar with no apparent outlying arrays. Pearson correlation coefficients and Spearman rank coefficients were computed on the RMA expression values (log_2_-transformed) for each set of biological triplicates.

To determine whether genes were differentially expressed between the treatments (well-watered and drought stressed) and amongst the three berry tissues (pulp, skin, seed), an ANOVA was performed on the RMA expression values. (For an overview on the application of ANOVA to microarray data, please see [123] and [124]). The following model was used for this analysis: *y*_*ijk *_= *T*_*ik *_+ *R*_*jk *_+ (*TR*)_*ijk *_where *y*_*ijk *_denotes the log_2 _signal measured for experimental state *i*, berry region *j*, and biological replicate *k*, with 1 ≤ *i *≤ 2, 1 ≤ *j *≤ 3, and 1 ≤ *k *≤ 3. The terms *T*_*i*_  and *R*_*j *_measure the effect of the experimental treatment and berry tissue, respectively, and the interaction term (*TR*)_*ijk *_accounts for the interaction between treatment and tissue. An ANOVA was performed on each gene using the linear model above, and three contrasts based on differences between the two treatments for each individual region. The same model was also used to perform a simple 1-way analysis between the three tissues under well-watered conditions only. Testing was performed for three tissue-specific contrasts under well-watered conditions with 3 biological replicates: pulp versus skin; pulp versus seed; skin versus seed and on the water status contrasts for each tissue type with 3 biological replicates: pulp stressed versus pulp well-watered; seed stressed versus seed well-watered; skin stressed versus skin well-watered. The R package limma was used for ANOVA methods [124]. A multiple testing adjustment [125] was performed on the *t*-statistics of each contrast to adjust the false discovery rate, and on the 1-way ANOVA *F*-statistics. Differentially expressed genes with adjusted *p*-value < 0.05 were extracted for further inspection.

The normalized and filtered data sets were clustered via hierarchical clustering with the complete agglomeration method and Euclidean distance metric [126]. This method resulted in the best resolution of 12 distinct clusters illustrating tissue-specific expression patterns. Heatmaps were generated using the using the MultiExperiment Viewer (MEV) software package [127]. To test for significant differences in the representation of Unigenes within each functional category per cluster, a Fisher's exact test was performed for each functional category within each cluster against the expected number of Unigenes in that category based on the overall *Vitis *GeneChip^® ^genome array expression distribution (see last column of Tables 2 and 3). An adjustment of the false discovery rate was made to account for the multiple hypothesis tests [125].

### Unigene annotation and functional analysis

Unigene annotation was updated by nucleotide sequence query of the probe consensus sequence against the UniProt/TrEMBL, NCBI-nr and TAIR protein databases using BLASTX (e-value < 1e-05). Functional categories were assigned automatically by amino acid homology to *Arabidopsis thaliana *proteins categorized according to the Munich Information Center for Protein Sequences (MIPS) [128] Funcat 1.3 classification scheme [129]. Bibliographic searches were performed to assign functions to Unigenes without *Arabidopsis *homologs.

### Quantitative RT-PCR

First strand synthesis was performed on the triplicated mRNA pool used for microarray experiments using the IScript™ cDNA synthesis kit (Bio-Rad, Hercules, CA) according to the manufacturer instructions. Primers and cDNA were mixed with iTaq SYBR Green Supermix (Bio-Rad). Real time RT-PCR was performed with ABI PRISM 7000 Sequence Detection System (Applied Biosystems, Foster City, CA) under conditions of 50°C for 2 minutes, 95°C for 10 minutes, then 40 cycles of 95°C for 15 seconds and 53°C for 1 minute and analyzed with ABI PRISM 700 SDS software. Data were calculated from the calibration curve and normalized using the expression curve of a GTP binding protein (TC39426), whose mRNA presented an extremely low coefficient of variation (0.054; M value = 0.17) through microarray analysis according to [130]. Primers sequences are provided in Additional file 2.

## Authors' contributions

JG conceived the experimental design, performed microarray, bioinformatics and RT-PCR analyses, prepared figures and tables, and wrote initial manuscript draft. LGD set up mRNA extraction protocol. KAS performed statistical analysis. RLT performed database analysis and annotation. MDW acquired physiological data. JCC, KAS, and GRC supervised its design and coordination and participated in the preparation of the manuscript. JCC finalized the written manuscript and conceived the study.

## Supplementary Material

Additional file 1 Heatmap and dendrogram of the 12 hierarchical clusters (A-L) defined for the three major tissues of grape berry (pulp, skin, seed) by clustering of log_2 _ratios of RMA values relative to the average value among the three tissues. The color scale indicates the extent of expression change: black (0), red (1+) to green (-1). Click here for file

Additional file 2 Table of primers used for quantitative reverse transcription-polymerase chain reaction (qRT-PCR). Click here for file

Additional file 3 Tables 1–13. Expression and putative function of relevant transcripts differentially expressed among tissues in berries harvested from well-watered vines. Click here for file

Additional file 4 Tables 14–21. Expression and putative function of relevant transcripts differentially expressed in one or more berry tissues according to water status. Click here for file

Additional file 5 Tables 22 and 23. Expression and putative function of all differentially expressed transcripts expressed in one or more berry tissues according to water status. Click here for file
