# Supplementary figures and images for: Tissue-specific mRNA expression profiling in grape berry tissues
Source: BMC Genomics. 2007 Jun 21;8:187. doi: 10.1186/1471-2164-8-187 (PMC1925093; doi:10.1186/1471-2164-8-187)

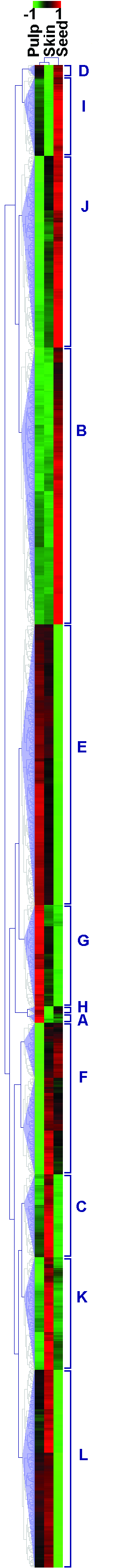

Supplement: Additional file 1 — Heatmap and dendrogram of the 12 hierarchical clusters (A-L) defined for the three major tissues of grape berry (pulp, skin, seed) by clustering of log2 ratios of RMA values relative to the average value among the three tissues. The color scale indicates the extent of expression change: black (0), red (1+) to green (-1). [file 1471-2164-8-187-S1.tiff]
