# Supplementary material for: Tissue-specific mRNA expression profiling in grape berry tissues
Source: BMC Genomics. 2007 Jun 21;8:187. doi: 10.1186/1471-2164-8-187 (PMC1925093; doi:10.1186/1471-2164-8-187)
Supplement: Additional file 2 — Table of primers used for quantitative reverse transcription-polymerase chain reaction (qRT-PCR). [file 1471-2164-8-187-S2.doc]

**Additional file 1:**

## Primers used for quantitative reverse transcription-polymerase chain reaction (RT-PCR).

| **Unigene** | **Description** | Forward primer | **Reverse primer** |
| --- | --- | --- | --- |
| AB073013 | Myb factor | 5’-gatgtgggcttctgggatac-3’ | 5’-agggagtagagtatgaatgcaaga-3’ |
| TC38121 | Aquaporin | 5’-tcaaggctcttgggtcattc-3’ | 5’-tcatgccctcatccatatca-3’ |
| TC50577 | Mlo | 5’-aaagcgagctcatggctaaa-3’ | 5’-ttgactcactgcccttgttg-3’ |
| TC38365 | Fructose bisphosphate aldolase | 5’-gctcagctcgggaagtacac-3’ | 5’-tgcaagtgcagcagcttagt-3’ |
| TC38541 | Pectate lyase | 5’-gagccaagtcctcttccat-3’ | 5’-cccattcaattaactgagatttac-3’ |
| TC38296 | RD22 | 5’-tgctcaaagttaagccagga-3’ | 5’-gcgcggaactaacaaatctc-3’ |
| TC41344 | Potassium transporter | 5’-cattcctcatgctgctcttg-3’ | 5’-atcaagctgcataggcacat-3’ |
| TC44975 | Carotenoid cleavage dioxygenanse | 5’-aggtgacgggcattatcaag-3’ | 5’-aggctcacgagggacaaag-3’ |
| TC45186 | Germin | 5’-aacgatttgccaactgaactg-3’ | 5’-cttgatgggtccaaaggaat-3’ |
| TC45472 | Anthocyanidin synthase | 5’-ccaggaggctctactctcca-3’ | 5’-tgggaagcttgaaacacagac-3’ |
| TC46783 | MAP kinase 4 | 5’-catctggagggaatccgtaa-3’ | 5’-aaaccctgcaaatggttctg-3’ |
| TC42536 | NCED | 5’-gtggaaggcaaggaaatgaa-3’ | 5’-ggcagagatgtgaaggaagg-3’ |
| TC41552 | Sodium-dicarboxylate transporter | 5’-caaatccctgcaaccagtct-3’ | 5’-aatatgccaagagccaatgc-3’ |
| TC46366 | Wound induced protein | 5’-agagaggagaaggccaagca-3’ | 5’-ggaatatgccctccctggat-3’ |
| TC48190 | CTR1 | 5’-caagaaattggcggactaca-3’ | 5’-ttcccttgaggactgcttgt-3’ |
| TC50572 | ABI3 | 5’-gggagtggagctggtgaag-3’ | 5’-agtaaaggccacggagacg-3’ |
| CF204393 | Anthocyanidin reductase | 5’-cgggattgaagaaatttatgatg-3’ | 5’-tagggaagaagggacagcaa-3’ |
| TC39641 | Leucoanthocyanidin reductase | 5’-ttcgatgagttcgtggagaa-3’ | 5’-agagaactggcggtgatcag-3’ |
| TC40715 | Sugar transporter | 5’-ggaacaaatccaggctacca-3’ | 5’-ggtacgaatcagctgccata-3’ |
| TC39426 | GTP-binding protein | 5’-agccatgtatcttcaccaacct-3’ | 5’-acccagcactctgcagtctt-3’ |
