# Supplementary material for: Tissue-specific mRNA expression profiling in grape berry tissues
Source: BMC Genomics. 2007 Jun 21;8:187. doi: 10.1186/1471-2164-8-187 (PMC1925093; doi:10.1186/1471-2164-8-187)
Supplement: Additional file 3 — Tables 1–13. Expression and putative function of relevant transcripts differentially expressed among tissues in berries harvested from well-watered vines. [file 1471-2164-8-187-S3.doc]

Table 1. Tissue specific expression of mRNA encoding flavonoid biosynthetic enzymes. Values are expressed as log2 ratio from the average expression of mRNA among tissues. Color code for tables 1-13: bright green: value<-1; green: -1<value<-0.5; light green: -0.5<value<0; light red 0<value<0.5; red: 0.5<value<1; bright red: 1< value. Vv indicates a significant match with a *Vitis vinifera* gene.

| **Cluster** | **Unigene** | **Affy probe** | **Top match N°** | **Description** | **Pulp** | **Skin** | **Seed** |
| --- | --- | --- | --- | --- | --- | --- | --- |
| *General phenylpropanoid pathway* | | | | | | | |
| F | TC39021 | 1619642_at | AAK62030 | Phenylalanine ammonia-lyase 1 | -4.48 | 1.2 | -0.62 |
| K | CK136925 | 1613113_at | AAQ74878 | Phenylalanine ammonia lyase | -3.76 | 1.38 | -1.61 |
| F | TC39071 | 1610206_at | BAA05643 | Phenylalanine ammonia lyase | -1.52 | 0.44 | 0.37 |
| K | TC38780 | 1616191_s_at | AAG10196 | Cinnamate-4-hydroxylase | -2.17 | 1.13 | -0.77 |
| F | TC41731 | 1619320_at | CAC36095 | 4-coumarate:Coenzyme A ligase | -1.93 | 0.92 | -0.25 |
| F | TC44994 | 1609307_at | AAC39366 | 4-coumarate:Coenzyme A ligase | -1.53 | 0.97 | -0.52 |
| *Lignin biosynthesis* | | | | | | | |
| E | TC46173 | 1608094_at | AAT39157 | Cinnamoyl CoA reductase | 0.42 | 0.48 | -1.93 |
| B | TC41296 | 1616445_at | CAB87637 | Cinnamoyl CoA reductase- | -0.96 | -1.1 | 1.02 |
| J | TC47787 | 1619513_at | AAM13142 | Cinnamoyl-CoA reductase | -1.5 | -0.73 | 1.03 |
| F | TC39574 | 1619065_at | AAP55155 | Cinnamoyl-CoA reductase | -1.3 | 0.64 | 0.05 |
| K | TC40043 | 1608048_at | AAP55155 | Cinnamoyl-CoA reductase | -2.8 | 1.29 | -1.3 |
| K | TC48483 | 1620650_s_at | AAD10327 | Cinnamyl alcohol dehydrogenase | -1.03 | 0.83 | -0.45 |
| K | TC46062 | 1613900_at | AAK28509 | Cinnamyl alcohol dehydrogenase | -0.74 | 0.77 | -0.52 |
| L | TC39339 | 1615439_at | CAA44216 | Cinnamyl-alcohol dehydrogenase | 0.17 | 0.39 | -0.82 |
| K | TC40554 | 1621227_at | AAC06319 | Cinnamyl-alcohol dehydrogenase | -0.36 | 0.59 | -0.47 |
| *Flavonoid biosynthesis* | | | | | | | |
| F | TC45343 | 1615447_at | BAB84111 | Chalcone synthase (Vv) | -4.23 | 1.28 | -0.95 |
| F | AF020709 | 1607732_at | AAB72091 | Chalcone synthase (Vv) | -5.12 | 1.18 | -0.49 |
| F | X&5969 | 1617019_at | CAA53583 | Chalcone synthase (Vv) | -4.36 | 0.39 | 0.71 |
| F | TC39321 | 1615912_at | CAA53577 | Chalcone isomerase (Vv) | -2.45 | 0.76 | 0.17 |
| F | TC38410 | 1620424_at | AAL36093 | Chalcone isomerase (Vv) | -1.47 | 0.61 | 0.15 |
| F | TC45490 | 1607607_s_at | AAT68774 | Flavanone 3-hydroxylase | -1.23 | 0.71 | -0.09 |
| K | TC45282 | 1607739_at | CAA53579 | Flavanone 3-hydroxylase (Vv) | -2.42 | 1.16 | -0.77 |
| G | CF202029 | 1608379_at | AAK622420 | Flavanone 3-hydroxylase | 0.57 | -0.17 | -0.67 |
| C | BQ798614 | 1612699_at | AAD43161 | Ethylene-forming-enzyme dioxygenase | -1.03 | 1.11 | -1.5 |
| J | TC49495 | 1622850_at | AAB88878 | Ethylene-forming-enzyme dioxygenase | -1.99 | -0.53 | 1.04 |
| K | TC45860 | 1611847_at | AAP31058 | Flavonoid 3',5'-hydroxylase | -5.58 | 1.54 | -3.82 |
| H | TC46972 | 1608791_at | AAO63023 | Flavonol synthase | 0.61 | -1.2 | 0.05 |
| I | TC40373 | 1610610_at | XP_48298 | Flavonol synthase | -0.2 | -0.51 | 0.51 |
| L | TC46143 | 1618551_at | AAP86222 | Flavonol synthase (Vv) | -1.61 | 1.3 | -2.23 |
| I | TC38926 | 1620675_at | CAA72420 | Dihydroflavonol 4-reductase(Vv) | -0.36 | -0.6 | 0.64 |
| G | TC41304 | 1621163_at | AAT39306 | Dihydroflavonol-4-reductase | 0.46 | -0.05 | -0.6 |
| F | TC42055 | 1611309_at | BAD45548 | Dihydroflavonol-4-reductase | -1.39 | 0.25 | 0.52 |
| *Anthocyanin biosynthesis* | | | | | | | |
| K | TC45472 | 1609765_s_at | BAC07545 | Anthocyanidin synthase | -3.54 | 1.25 | -0.89 |
| E | TC40209 | 1614441_at | AAK63997 | Anthocyanidin synthase | 0.27 | 0.45 | -1.21 |
| C | AF000371 | 1617171_s_at | AAB81682 | UDP glucose:flavonoid 3-O-glucosyltransferase (Vv) | -4.15 | 1.5 | -3.09 |
| L | AB047090 | 1619788_at | BAB41017 | UDP-glucose:flavonoid 3-O-glucosyltransferase (Vv) | -1.46 | 1.21 | -1.61 |
| F | TC43892 | 1619698_at | AAU09445 | UDP-rhamnose: rhamnosyltransferase | -2.22 | 0.37 | 0.58 |
| *Proanthocyaidin biosynthesis* | | | | | | | |
| K | TC39641 | 1615174_s_at | AAX12185 | Leucoanthocyanidin reductase | -1.60 | 0.21 | 0.59 |
| B | CF204393 | 1612134_at | BAD89742 | Anthocyanidin reductase (Vv) | -1.17 | -1.48 | 1.14 |
| K | TC42471 | 1616898_at | AAN61072 | O-methyltransferase | -4.98 | 1.51 | -3.07 |

Table 2. Tissue specific expression of mRNA encoding cell wall metabolism enzymes. Values are expressed as log2 ratio from the average expression of mRNA among tissues. Vv indicates a significant match with a *Vitis vinifera* gene.

| **Cluster** | **Unigene** | **Affy probe** | **Top match N°** | **Description** | **Pulp** | **Skin** | **Seed** |
| --- | --- | --- | --- | --- | --- | --- | --- |
| *Pectin metabolism* | | | | | | | |
| L | TC49646 | 1621225_at | CAA23048 | Polygalacturonase | 0.17 | 0.67 | -1.82 |
| A | AY043233 | 1620305_at | AAK81876 | Polygalacturonase PG1 (Vv) | 1.31 | -2.58 | -1.5 |
| L | TC47230 | 1618849_at | CAA23048 | Polygalacturonase | 0.17 | 0.68 | -1.87 |
| G | TC47738 | 1608756_at | AAP33475 | Polygalacturonase- | 1.14 | -0.7 | -2.54 |
| F | TC46487 | 1620140_at | AAA34181 | Polygalacturonase | -1.27 | 0.83 | -0.31 |
| E | TC51164 | 1608781_at | CAB62015 | Endo-polygalacturonase- | 0.41 | 0.04 | -0.63 |
| L | TC38541 | 1618576_at | AAK66161 | Pectate lyase | 0.18 | 0.73 | -2.25 |
| K | TC45703 | 1618297_s_at | AAQ84042 | Pectate lyase | -2.26 | 1.3 | -1.63 |
| C | TC47054 | 1608799_at | NP_915049 | Pectin methylesterase | -1.02 | 1.12 | -1.58 |
| G | TC50564 | 1612672_at | CAA96434 | Pectin methylesterase | 0.5 | -0.11 | -0.61 |
| F | TC46866 | 1611095_a_at | BAA95794 | Pectinesterase DC1.2 | -2.24 | 0.11 | 0.77 |
| E | TC40743 | 1617785_at | BAA95794 | Pectinesterase DC1.2 | 0.46 | 0.17 | -0.99 |
| L | AY043232 | 1619468_at | AAK81875 | Pectin methylesterase inhibitor (Vv) | 0.3 | 0.6 | -1.95 |
| L | TC46390 | 1622535_at | AAO41962 | Pectin methylesterase inhibitor | -0.18 | 0.94 | -2.31 |
| E | TC40734 | 1606847_at | CAC69340 | Pectin methylesterase inhibitor | 0.43 | 0.15 | -0.89 |
| L | TC45270 | 1621352_at | CAC69338 | Pectin methylesterase inhibitor | 0.35 | 0.41 | -1.34 |
| C | TC39366 | 1610996_at | CAA59482 | Pectin methylesterase inhibitor | -0.96 | 1.15 | -1.89 |
| B | CF373209 | 1612763_at | CAB85625 | Pectin methylesterase inhibitor (Vv) | -2.48 | -2.54 | 1.41 |
| B | TC48184 | 1616154_at | NP_178270 | Pectin methylesterase inhibitor | 0.7 | -0.94 | 0.9 |
| *Xyloglucan metabolism* | | | | | | | |
| L | TC38403 | 1610418_at | AAO15998 | Expansin | 0.13 | 0.56 | -1.21 |
| L | BQ794765 | 1619010_s_at | BAC66695 | Expansin (Vv) | -0.1 | 0.72 | -1.25 |
| E | TC38812 | 1608074_s_at | BAC66694 | Expansin (Vv) | 0.84 | -0.15 | -1.67 |
| B | TC40522 | 1613527_at | AAK48847 | Expansin | -3.42 | -3.87 | 1.5 |
| C | TC38813 | 1617150_at | BAC66694 | Expansin (Vv) | -2.2 | 1.43 | -3.52 |
| I | TC46149 | 1607674_at | CAE12163 | Expansin | -0.15 | -0.89 | 0.64 |
| L | TC38624 | 1610071_at | AAA50175 | Expansin | 0.3 | 0.33 | -0.95 |
| C | TC46110 | 1620840_at | AAM89261 | Expansin | -2.03 | 1.34 | -2.19 |
| B | TC45154 | 1618848_at | AAS46241 | Xyloglucan endotransglucosylase | -2.56 | -2.73 | 1.42 |
| G | TC46220 | 1620096_at | CAB77806 | xyloglucan endotransglucosylase | 0.69 | -0.3 | -0.8 |
| K | TC38792 | 1617739_at | AAC09388 | xyloglucan endotransglycosylase | -0.91 | 0.96 | -0.92 |
| E | TC45396 | 1615809_at | AAB18365 | Xyloglucan endotransglycosylase | 0.56 | -0.06 | -0.8 |
| E | AB074999 | 1613415_at | BAB78506 | Xyloglucan endotransglycosylase (Vv) | 0.58 | 0.03 | -1.06 |
| D | TC38793 | 1608945_at | AAA81350 | Xyloglucan endotransglycosylase | 0.08 | -1.29 | 0.62 |
| I | TC42884 | 1614426_at | AAA81350 | Xyloglucan endotransglycosylase | 0.11 | -0.76 | 0.57 |
| B | CF206328 | 1609909_s_at | AAO65151 | Xyloglucan endotransglycosylase | -0.45 | -0.52 | 0.65 |
| G | CF372200 | 1618150_at | AAF80591 | Xyloglucan endotransglycosylase | 0.68 | -0.15 | -1.02 |
| E | TC45088 | 1616957_at | AAD31066 | glycosyl hydrolase / cellulase | 0.76 | 0.16 | -2.39 |
| E | TC45090 | 1620050_at | AAD31066 | glycosyl hydrolase / cellulase | 0.34 | 0.19 | -0.76 |
| E | TC47657 | 1606832_at | AAP40422 | glycosyl hydrolase / cellulase | 0.54 | 0.35 | -1.87 |
| E | TC39280 | 1616192_at | CAC43565 | Glucan endo-1,3-beta-glucosidase | 0.59 | 0 | -1.01 |
| L | TC42155 | 1607374_at | AAQ17461 | Glucan endo-1,3-beta-glucosidase | 0.1 | 0.43 | -0.79 |
| J | TC46813 | 1619383_at | BaD91080 | Beta-D-galactosidase | -1.21 | -0.47 | 0.88 |

Table 3. Tissue specific expression of mRNA encoding proteins involved in photosynthesis and carbon assimilation. Values are expressed as log2 ratio from the average expression of mRNA among tissues. Vv indicates a significant match with a *Vitis vinifera* gene.

| **Cluster** | **Unigene** | **Affy probe** | **Top match N°** | **Description** | **Pulp** | **Skin** | **Seed** |
| --- | --- | --- | --- | --- | --- | --- | --- |
| *Photosynthesis* | | | | | | | |
| C | TC39094 | 1622534_at | AAO49652 | Photosystem I-N subunit | -0.9 | 1.04 | -1.28 |
| K | TC38439 | 1613773_s_at | CAA59409 | Protein of photosystem II | -0.79 | 0.93 | -0.97 |
| F | TC38326 | 1622302_s_at | BAA96365 | Oxygen evolving enhancer protein 1 | -3.16 | 1.22 | -0.84 |
| J | TC44135 | 1612480_at | AAW28572 | Oxygen evolving enhancer protein 3 | -1.1 | -0.37 | 0.82 |
| F | TC38964 | 1613428_at | AAW80966 | Chloroplast oxygen-evolving protein 16 kDa subunit | -3.01 | -0.53 | 1.13 |
| K | TC45298 | 1613494_s_at | BAA89317 | 23kDa polypeptide of the oxygen-evolving complex of photosystem II | -2.82 | 1.21 | -0.89 |
| L | TC38668 | 1607793_at | AAP23938 | One helix protein | -0.23 | 0.79 | -1.27 |
| K | TC45056 | 1611733_s_at | CAD89270 | Photosystem I reaction centre PSI-D subunit precursor | -1.73 | 1.19 | -1.25 |
| F | TC47099 | 1621374_at | AAV44065 | Chlorophyll synthase | -0.76 | 0.48 | 0.02 |
| E | TC47896 | 1615283_at | AAL36411 | Light harvesting pigment protein | 0.57 | 0.14 | -1.26 |
| K | TC45098 | 1609044_at | S14305 | Chlorophyll a/b-binding | -0.91 | 0.89 | -0.71 |
| *Malate biosynthesis* | | | | | | | |
| I | TC41812 | 1613513_at | AAL08235 | Alanine aminotransferase | -0.39 | -0.84 | 0.75 |
| F | TC45311 | 1618004_s_at | BAD30627 | Alanine aminotransferase | -1.76 | 0.03 | 0.75 |
| F | TC39041 | 1611274_at | CAA52614 | Malate dehydrogenase (NADP+) | -0.86 | 0.59 | -0.08 |
| J | TC46453 | 1612414_at | CAA55143 | Pyruvate,orthophosphate dikinase | -3.4 | -1.04 | 1.27 |
| L | TC40018 | 1608100_at | AAL83719 | PEP carboxylase (Vv) | -0.76 | 1.16 | -2.46 |
| L | TC44116 | 1616325_at | AAS67005 | Phosphoenolpyruvate carboxylase | -0.26 | 0.76 | -1.09 |
| B | TC39883 | 1621397_at | AAA79371 | Aspartate aminotransferase | -0.65 | -0.57 | 0.76 |
| B | TC46518 | 1607149_at | BAD54126 | Aspartate transaminase, mitochondrial | -0.32 | -0.47 | 0.56 |
| I | TC45858 | 1613165_s_at | AAK43976 | L-iditol 2-dehydrogenase | -1.66 | -3.24 | 1.37 |
| *Calvin Benson cycle* | | | | | | | |
| B | TC42635 | 1609079_at | AAK56244 | Pyruvate kinase | -0.62 | -0.53 | 0.73 |
| E | TC43227 | 1613060_at | CAB66395 | Pyruvate kinase | 0.59 | 0.2 | -1.54 |
| L | TC40404 | 1614016_at | CAA82223 | Pyruvate kinase, plastid isozyme | 0.27 | 0.32 | -0.87 |
| L | TC38352 | 1614178_at | AAP83929 | Rubisco activase alpha form | -0.75 | 1.11 | -2.02 |
| K | TC38353 | 1616918_s_at | AAP83929 | Rubisco activase alpha form | -3.84 | 1.48 | -2.9 |
| B | TC40753 | 1618095_at | CAC80377 | Glyceraldehyde-3-phosphate dehydrogenase | -0.41 | -0.44 | 0.6 |
| K | TC38629 | 1617428_at | AAA84543 | Glyceraldehyde-3-phosphate dehydrogenase B subunit | -1.64 | 1.09 | -0.86 |
| C | TC38365 | 1616002_s_at | AAM46780 | Fructose bisphosphate aldolase | -1.76 | 1.31 | -2.19 |
| C | TC38366 | 1621944_at | AAM46780 | Fructose-bisphosphate aldolase | -1.72 | 1.32 | -2.3 |
| B | TC40284 | 1612272_at | CAA54265 | Fructose-1,6-bisphosphatase | -1.69 | -1.21 | 1.17 |
| C | TC45985 | 1622065_at | AAD25541 | Fructose-1,6-bisphosphatase | -1.28 | 1.14 | -1.37 |
| F | TC38767 | 1617670_at | AAA66289 | Triosephosphate isomerase, chloroplast isozyme | -0.9 | 0.23 | 0.37 |
| K | TC38991 | 1609568_at | AAO33154 | Transketolase | -0.71 | 0.9 | -0.92 |
| F | TC38803 | 1618284_at | AAO22558 | Sedoheptulose-1,7-bisphosphatase | -2.35 | 0.65 | 0.3 |
| F | TC45008 | 1618020_x_at | BAA23214 | Small subunit of ribulose-1,5-bisphosphate carboxylase | -4.57 | 0.86 | 0.19 |
| I | TC46859 | 1607831_at | NP_172452 | Rubisco subunit binding-protein beta subunit | -1.24 | 0.04 | 0.63 |

Table 4. Tissue specific expression of mRNA encoding proteins involved in terpenoid pathways. Values are expressed as log ratio from the average expression of mRNA among tissues. Vv indicates a significant match with a *Vitis vinifera* gene.

| **Cluster** | **Unigene** | **Affy probe** | **Top match N°** | **Description** | **Pulp** | **Skin** | **Seed** |
| --- | --- | --- | --- | --- | --- | --- | --- |
| *Isoprenol pathway* | | | | | | | |
| C | TC48118 | 1617024_at | AAQ84169 | 1-deoxy-D-xylulose 5-phosphate synthase | -2.28 | 1.4 | -2.7 |
| F | TC50523 | 1607622_at | AAL37041 | 3-hydroxy-3-methylglutaryl coenzyme A reductase | -0.7 | 0.38 | 0.11 |
| L | TC49029 | 1614923_at | AAB69727 | 3-hydroxy-3-methylglutaryl coenzyme A reductase | 0.3 | 0.32 | -0.94 |
| L | TC45366 | 1608252_s_at | AAS46245 | HMG-CoA synthase | 0.22 | 0.41 | -0.98 |
| L | TC47115 | 1621391_at | AAL18925 | Mevalonate kinase | 0.09 | 0.49 | -0.93 |
| L | TC40923 | 1616679_at | AAL18927 | Mevalonate disphosphate decarboxylase | -0.05 | 0.56 | -0.83 |
| L | TC38778 | 1606675_at | BAB40974 | Isopentenyl diphosphate isomerase 2 | 0.25 | 0.3 | -0.78 |
| *Sterol biosynthesis* | | | | | | | |
| F | TC39028 | 1607421_at | BAA22559 | Squalene synthase | -0.65 | 0.44 | 0.01 |
| B | TC40984 | 1612781_at | CAD23248 | Squalene monooxygenase 2 | -0.91 | -1.44 | 1.07 |
| I | TC43543 | 1615654_at | CAD23248 | Squalene monooxygenase 2 | -0.84 | -1.48 | 1.06 |
| G | TC42197 | 1615604_at | AAS66357 | Terpene synthase (Vv) | 0.6 | -0.35 | -0.5 |
| G | TC40259 | 1616910_at | AAT86042 | E-beta-ocimene synthase | 0.65 | -0.22 | -0.8 |
| *Carotenoids biosynthesis* | | | | | | | |
| B | TC40542 | 1610176_at | BAB60678 | Geranylgeranyl diphosphate synthase | -1.13 | -1.03 | 1.04 |
| K | TC46134 | 1620751_at | BAA84763 | Phytoene synthase | -1.01 | 0.92 | -0.73 |
| L | TC48107 | 1621899_at | AAQ04224 | Zeta-carotene desaturase ZDS1 | 0.16 | 0.56 | -1.31 |
| B | TC42069 | 1619371_at | AAM77007 | Beta-carotene hydroxylase (Vv) | -1.28 | -1.47 | 1.16 |
| *Carotenoids catabolism* | | | | | | | |
| C | TC46508 | 1618171_s_at | AAR11195 | Zeaxanthin epoxidase (Vv) | -0.58 | 0.87 | -1 |
| K | TC39841 | 1619925_at | CAA65048 | zeaxanthin epoxidase | -0.68 | 0.74 | -0.5 |
| K | TC47195 | 1611998_at | AAL67858 | Violaxanthin de-epoxidase | -0.53 | 0.59 | -0.32 |
| L | TC44975 | 1610743_s_at | AAX48772 | Carotenoid cleavage dioxygenase (Vv) | 0.04 | 0.67 | -1.37 |
| G | TC43255 | 1610455_at | CAB07784 | Nine-cis-epoxycarotenoid dioxygenase | 1.07 | -0.51 | -2.31 |
| G | TC42536 | 1607029_at | CAC34025 | Nine-cis-epoxycarotenoid dioxygenase | 1.02 | -0.57 | -1.72 |
| G | TC48377 | 1608022_at | AAR11193 | Nine-cis-epoxycarotenoid dioxygenase (Vv) | 0.52 | -0.07 | -0.71 |

Table 5. Tissue specific expression of mRNA encoding proteins involved in pathogen response. Values are expressed as log ratio from the average expression of mRNA among tissues. Vv and Vr indicate a significant match with *Vitis vinifera* and Vitis riparia genes, respectively.

| **Cluster** | | **Unigene** | | **Affy probe** | **Top match N°** | | **Description** | | **Pulp** | **Skin** | | **Seed** | |
| --- | --- | --- | --- | --- | --- | --- | --- | --- | --- | --- | --- | --- | --- |
| *Pathogenesis-related (PR) proteins* | | | | | | | | | | | | | |
| K | | TC47119 | | 1611058_at | CAD60273 | | PR1 (Vv) | | 0.67 | 0.79 | | -0.65 | |
| K | | TC45727 | | 1620063_at | CAB91554 | | PR2 Beta 1-3 glucanase (Vv) | | -2.96 | 1.32 | | -1.41 | |
| B | | TC44117 | | 1619916_s_at | AAR06588 | | PR2 beta-1,3-glucanase (Vv) | | 0.66 | -0.65 | | 0.79 | |
| B | | TC46036 | | 1610324_a_at | S31196 | | PR2 beta-1,3-glucanase | | -0.81 | -1.15 | | 0.98 | |
| E | | TC44867 | | 1608203_at | AAK85402 | | PR2 beta-1,3-glucanase | | 0.47 | 0.04 | | -0.77 | |
| B | | TC46138 | | 1614551_at | AAQ56598 | | PR3 Chitinase | | -2.91 | -2.75 | | 1.44 | |
| I | | TC39952 | | 1611552_at | CAA77656 | | PR3 Acidic chitinase III | | -0.54 | -1.01 | | 0.86 | |
| J | | Z54234 | | 1620505_at | CAA90970 | | PR3 Basic Chitinase (Vv) | | -1.3 | -0.08 | | 0.72 | |
| L | | TC47573 | | 1606625_at | AAM95447 | | PR3 Class IV chitinase (Vv) | | 0.04 | 0.62 | | -1.2 | |
| C | | TC38434 | | 1621319_s_at | AAQ10093 | | PR3 Class IV chitinase (Vv) | | -0.38 | 0.63 | | -0.56 | |
| F | | AY137377 | | 1617192_at | AAM95447 | | PR3 Class IV chitinase (Vv) | | -0.97 | 0.24 | | 0.39 | |
| K | | TC38693 | | 1618835_s_at | AAC33732 | | PR4 PR-4 type protein (Vv) | | -2.26 | 1.2 | | -1.03 | |
| B | | AF003007 | | 1616413_at | AAB61590 | | PR5 Thaumatin VVTL1 (Vv) | | -0.39 | -0.47 | | 0.6 | |
| B | | AF178653 | | 1613180_at | AAD55090 | | PR5 Thaumatin (Vr) | | -1.66 | -1.12 | | 1.15 | |
| F | | AF532965 | | 1620390_s_at | AAQ10092 | | PR5 Thaumatin (Vv) | | -1.28 | 0.47 | | 0.26 | |
| L | | TC38862 | | 1622374_at | AAB41124 | | PR5 Thaumatin | | 0.37 | 0.53 | | -1.94 | |
| F | | TC47844 | | 1618871_at | CAB37522 | | PR5 Thaumatin | | -0.98 | 0.43 | | 0.2 | |
| F | | TC39447 | | 1606794_at | AAQ10092 | | PR5 Thaumatin (Vv) | | -1.16 | 0.36 | | 0.34 | |
| B | | AY156047 | | 1611666_s_at | AAN85825 | | PR6 Protease inhibitor (Vv) | | -5.97 | -5.33 | | 1.57 | |
| B | | TC38119 | | 1619031_at | 1204170A | | PR6 Protease inhibitor | | -4.6 | -5.14 | | 1.55 | |
| B | | TC39394 | | 1620137_s_at | AAG17880 | | PR6 Kunitz trypsin inhibitor protein | | -2.69 | -2.89 | | 1.44 | |
| B | | TC39799 | | 1609648_at | 2111250B | | PR6 Trypsin inhibitor | | -2.96 | -2.82 | | 1.45 | |
| B | | TC42241 | | 1609395_at | AAR92225 | | PR6 Cystatin | | -1.38 | -1.24 | | 1.13 | |
| E | | TC38691 | | 1617860_at | CAA75593 | | PR6 Protease inhibitor | | 0.61 | 0.33 | | -2.19 | |
| B | | CF512529 | | 1610583_at | BAB09314 | | PR10 Bet v I allergen | | -5.12 | -5.15 | | 1.56 | |
| C | | TC38484 | | 1612444_at | CAC83581 | | PR10 Bet v I allergen | | -2.4 | 1.4 | | -2.56 | |
| B | | TC42776 | | 1614450_at | AAK14060 | | PR10 Bet v I allergen | | -4.63 | -4.19 | | 1.54 | |
| L | | TC45121 | | 1617876_a_at | CAB85634 | | PR10 Bet v I allergen | | 0.18 | 0.36 | | -0.78 | |
| F | | AF467945 | | 1609281_s_at | AAO33393 | | PR14 Lipid transfer protein (Vv) | | -2.27 | 0.97 | | -0.25 | |
| K | | TC38438 | | 1622416_at | AAG29777 | | PR14 Lipid transfer protein | | -1 | 0.87 | | -0.57 | |
| K | | TC38608 | | 1614230_at | CAH04984 | | PR14 Lipid transfer protein | | -0.99 | 1.01 | | -1.04 | |
| G | | TC46011 | | 1611350_at | CAA56113 | | PR14 Lipid transfer protein | | 0.59 | -0.21 | | -0.67 | |
| B | | TC41261 | | 1608026_at | BAB10833 | | PR15 Germin | | -5.23 | -4.34 | | 1.55 | |
| *Other genes potentially involved in pathogen resistance* | | | | | | | | | | | | | |
| B | | TC46148 | | 1614030_at | CAB44031 | | Lectin | | -1.15 | -2.42 | | 1.24 | |
| B | | TC48215 | | 1612062_at | AAC78508 | | Phloem-specific lectin | | -0.71 | -0.96 | | 0.91 | |
| B | | TC46871 | | 1617848_at | BAA06186 | | Lectin | | -4.79 | -5.1 | | 1.55 | |
| B | | TC47689 | | 1618698_at | AAL86739 | | Lectin | | -1.32 | -1.47 | | 1.16 | |
| E | | TC48059 | | 1607812_at | CAB69837 | | Lectin | | 0.67 | 0.13 | | -1.67 | |
| B | | TC38776 | | 1607645_at | AAD38290 | | Pathogenesis related protein | | -3.32 | -2.8 | | 1.46 | |
| K | | TC38857 | | 1619115_s_at | AAO39937 | | Dirigent protein | | -1.74 | 1.24 | | -1.54 | |
| F | | TC42177 | | 1618663_s_at | AAQ89609 | | Dirigent protein | | -2.67 | 0.39 | | 0.61 | |
| B | | CF203567 | | 1608771_x_at | AAQ89609 | | Dirigent protein | | -1.07 | -0.8 | | 0.96 | |
| K | | TC40563 | | 1621901_at | NP_176113 | | Dirigent protein | | 0.53 | 0.72 | | -0.6 | |
| G | | TC41586 | | 1620598_at | AAR36911 | | Disease resistance avr9/cf-9 | | 1.01 | -0.52 | | -1.76 | |
| G | | TC47283 | | 1622760_at | AAG43551 | | Disease resistance avr9/cf9 | | 0.95 | -0.44 | | -1.58 | |
| F | | TC38208 | | 1611432_at | AAV92890 | | Disease resistance avr9/cf9 | | -0.8 | 0.29 | | 0.27 | |
| J | | TC45755 | | 1610721_s_at | AAG43557 | | Disease resistance avr9/cf9 | | -0.55 | -0.13 | | 0.49 | |
| J | | TC46932 | | 1614256_at | AAG43557 | | Disease resistance avr9/cf9 | | -0.66 | -0.37 | | 0.67 | |
| E | | TC42078 | | 1606987_at | CAB80047 | | Disease resistance NBS-LRR | | 0.41 | 0.36 | | -1.38 | |
| E | | TC40208 | | 1610085_at | AAF66615 | | Disease resistance NBS-LRR | | 0.59 | -0.06 | | -0.92 | |
| E | | TC40017 | | 1610054_at | AAL07540 | | Resistance gene analog NBS5 LRR | | 0.41 | 0.12 | | -0.78 | |
| C | | TC50577 | | 1614541_at | AAP54849 | | Mlo (pathogen resistance, mildew) | | -1.62 | 1.23 | | -1.64 | |
| E | CD798952 | | 1615403_at | | | AAV92898 | | Avr9\/Cf-9 rapidly elicited protein 102 | 0.39 | | 0.06 | | -0.64 |
| B | | TC41602 | | 1619391_at | O49621 | | Mlo (pathogen resistance, mildew) | | 0.43 | -0.44 | | 0.61 | |
| B | | TC48696 | | 1611135_at | AAL36348 | | Alpha-hydroxynitrile lyase | | -3.49 | -2.57 | | 1.46 | |
| K | | TC41501 | | 1620058_at | AAP51059 | | Latex cyanogenic beta glucosidase | | -1.62 | 1.14 | | -1.08 | |
| K | | TC40872 | | 1606948_at | CAD41660 | | Mandelonitrile lyase | | -3.83 | 1.42 | | -1.98 | |
| H | | AY039034 | | 1620808_at | AAK72100 | | Beta-glucosidase (Vv) | | 0.43 | -0.86 | | 0.14 | |
| C | | TC42830 | | 1611027_at | AAD10960 | | Cell wall invertase precursor | | -1.27 | 1.18 | | -1.63 | |

Table 6. Tissue specific expression of mRNA encoding proteins involved in aroma biosynthesis. Values are expressed as log ratio from the average expression of mRNA among tissues. Vv indicates a significant match with a *Vitis vinifera* gene.

| **Cluster** | **Unigene** | **Affy probe** | **Top match N°** | **Description** | **Pulp** | **Skin** | **Seed** |
| --- | --- | --- | --- | --- | --- | --- | --- |
| B | CF209780 | 1620469_at | AAM23005 | Orcinol O-methyltransferase | -2.75 | -2.75 | 1.43 |
| G | TC40103 | 1621151_at | AAM23004 | Orcinol O-methyltransferase | 0.56 | -0.34 | -0.45 |
| E | TC51257 | 1606852_at | AAT68601 | Benzoyl coenzyme A: benzyl alcohol benzoyl transferase | 0.37 | 0.15 | -0.74 |
| B | CA818350 | 1612552_at | AAG51997 | S-adenosyl-L-methionine:salicylic acid carboxyl methyltransferase | -4.92 | -3.6 | 1.53 |
| L | TC39566 | 1616348_at | AAO45013 | S-adenosyl-L-methionine:benzoic acid /salicylic acid carboxyl methyltransferase | 0.3 | 0.58 | -1.84 |
| J | TC41000 | 1608603_at | BAD18975 | phloroglucinol O-methyltransferase | -0.82 | -0.55 | 0.81 |
| B | CF209780 | 1620469_at | AAM23005 | Orcinol O-methyltransferase | -2.75 | -2.75 | 1.43 |
| J | CF207053 | 1620342_at | AAG43822 | Caffeic acid O-methyltransferase | -4.16 | -1.66 | 1.39 |
| L | AF239740 | 1616434_s_at | AAF44672 | Caffeic acid O-methyltransferase (Vv) | -0.62 | 1.07 | -1.99 |
| F | TC39874 | 1619983_at | CAB64217 | Caffeic acid O-methyltransferase | -0.74 | 0.18 | 0.35 |
| J | TC42415 | 1607475_s_at | AAG43822 | Caffeic acid O-methyltransferase | -3.62 | 1.55 | 1.37 |
| L | TC45576 | 1619450_s_at | AAF44672 | Caffeic acid O-methyltransferase (Vv) | -0.53 | 1.02 | 1.83 |
| B | TC48824 | 1613542_at | BAC78827 | Caffeic acid O-methyltransferase | -1.34 | -1.6 | 1.19 |
| B | TC46152 | 1606891_at | CAA71876 | cytochrome P450 | -2.01 | -2.51 | 1.36 |
| L | BQ797168 | 1607359_s_at | BAB12433 | (S)-N-methylcoclaurine-3'-hydroxylase | 0.32 | 0.37 | -1.14 |
| K | CF605243 | 1607805_s_at | AAN05418 | cytochrome P450 | -0.83 | 0.93 | -0.92 |
| G | TC40368 | 1608099_at | AAB17070 | Cytochrome P450 | 0.64 | -0.37 | -0.58 |
| L | TC40120 | 1609099_at | AAF89209 | Cytochrome P450 | 0.4 | 0.55 | -2.19 |
| C | CF205577 | 1609712_at | AAN05418 | Cytochrome P450 | -0.5 | 0.87 | -1.1 |
| B | CF206136 | 1610044_s_at | CAA71877 | Cytochrome P450 | -2.62 | -2.93 | 1.44 |
| C | TC47343 | 1610182_at | BAA96885 | cytochrome P450 | -1.43 | 1.24 | -1.88 |
| C | CA817595 | 1610194_at | AAO43566 | Cytochrome P450 | -1.63 | 1.27 | -1.92 |
| B | TC46216 | 1610325_at | CAA50312 | P450 hydroxylase | -3.52 | -3.55 | 1.5 |
| L | TC40732 | 1610415_at | CAB85635 | ripening-related P-450 enzyme (Vv) | -0.27 | 0.91 | -1.78 |
| L | TC41014 | 1610541_at | CAB85635 | ripening-related P-450 enzyme (Vv) | 0.25 | 0.61 | -1.82 |
| L | TC38210 | 1611038_at | BAB33421 | Senescence-associated protein | 0.13 | 0.61 | -0.84 |
| K | TC40306 | 1611306_at | CAA50646 | cytochrome P450 | -4.64 | 1.52 | -3.49 |
| K | CF405451 | 1611426_at | CAB85635 | ripening-related P-450 (Vv) | -2.91 | 1.43 | -2.6 |
| C | CF404908 | 1611829_at | AAU05534 | Cytochrome P450 | -0.51 | 0.83 | -0.95 |
| C | CF206021 | 1612511_at | CAA71514 | Cytochrome P450 | -0.49 | 0.81 | -0.89 |
| B | TC39254 | 1613619_at | CAB38203 | Cytochrome p450 | -1.59 | -1.07 | 1.13 |
| C | TC49681 | 1617051_at | AAL24168 | cytochrome P450 | -1.18 | 1.13 | -1.45 |
| J | TC38220 | 1619263_at | T02955 | cytochrome P450 | -0.53 | -0.32 | 0.59 |
| F | TC45772 | 1619689_at | AAK92762 | cytochrome P450 | -0.53 | 0.49 | -0.14 |
| C | TC47905 | 1621989_at | AAK93657 | Cytochrome P450 | -0.23 | 0.52 | -0.49 |
| B | TC42214 | 1622316_at | NP_176674 | Cytochrome P450 | -1.23 | -0.86 | 1.02 |
| F | TC48079 | 1622811_at | AAK59583 | cytochrome p450 | -0.79 | 0.58 | -0.11 |
| L | TC46255 | 1613920_at | AAB94589 | CYP83D1p | 0.32 | 0.4 | -1.23 |

Table 7. Tissue specific expression of mRNA encoding proteins involved in metabolite transport facilitation. Values are expressed as log ratio from the average expression of mRNA among tissues. Vv indicates a significant match with a *Vitis vinifera* gene.

| **Cluster** | | **Unigene** | **Affy probe** | **Top match N°** | **Description** | | **Pulp** | **Skin** | **Seed** |
| --- | --- | --- | --- | --- | --- | --- | --- | --- | --- |
| *Aquaporins* | | | | | | | | | |
| L | | TC38121 | 1615808_s_at | AAV69744 | Aquaporin PIP2-1 (Vv) | | 0.29 | 0.39 | -1.1 |
| E | | CK138165 | 1622502_at | AAV69744 | Aquaporin (Vv) | | 0.59 | 0.04 | -1.09 |
| E | | TC38281 | 1612244_s_at | AAF71820 | Aquaporin PIP2-2 (vb x vr) | | 0.57 | 0.37 | -2.13 |
| L | | TC38445 | 1614489_at | AAF80556 | Plasma membrane aquaporin (Vv) | | -0.06 | 0.61 | -0.96 |
| C | | AF188843 | 1607432_s_at | AAF80556 | Plasma membrane aquaporin (Vv) | | -0.29 | 0.69 | -0.8 |
| K | | TC38576 | 1615829_s_at | AAF78757 | Aquaporin TIP3 (vb x vr) | | -0.81 | 0.94 | -0.98 |
| B | | TC39811 | 1619180_at | AAB84183 | Beta-tonoplast intrinsic protein | | -5.06 | -5.57 | 1.56 |
| Calcium | | | | | | | | | |
| B | | TC49544 | 1613997_at | AAL73984 | Type IIB calcium ATPase | | -0.49 | -0.77 | 0.77 |
| L | | TC48993 | 1609778_at | AAD31896 | Calcium ATPase | | 0.27 | 0.37 | -1 |
| L | | TC40014 | 1614588_s_at | AAM61435 | Ca2+-transporting ATPase | | -0.07 | 0.72 | -1.31 |
| I | | CF404214 | 1622073_at | BAB03036 | Ca2+-transporting ATPase | | 0.16 | -0.59 | 0.53 |
| C | | CD797529 | 1612232_at | BAB01709 | Ca2+-transporting ATPase | | -0.25 | 0.54 | -0.51 |
| B | | TC46112 | 1617237_s_at | BAA25753 | Ca2+/H+ exchanger | | -2.57 | -2.22 | 1.39 |
| J | | TC48242 | 1612823_at | BAD15100 | Two-pore calcium channel | | -0.52 | -0.23 | 0.54 |
| K | | TC39122 | 1607957_a_at | AAL58918 | Potassium-dependent sodium-calcium exchanger | | -0.59 | 0.72 | -0.53 |
| *Sodium* | |  |  |  |  | |  |  |  |
| E | | TC48358 | 1611614_at | BAB11467 | Na+/H+ antiporter protein | | 0.68 | 0.31 | -2.68 |
| B | | TC41399 | 1611173_at | AAM08405 | Na+/H+ exchanger 4 | | -2.8 | -3.19 | 1.46 |
| *Potassium* | |  |  |  |  | |  |  |  |
| L | TC49359 | | 1610366_at | CAC05489 | Potassium channel | 0.33 | | 0.68 | -2.81 |
| L | | TC41344 | 1610123_at | AAL32825 | Potassium transporter | | -0.26 | 0.95 | -2.13 |
| E | | TC47746 | 1607241_at | AAM20451 | Potassium transporter | | 0.54 | 0.1 | -1.09 |
| F | | TC47109 | 1615318_at | CAB40777 | Potassium transporter | | -4.37 | -0.64 | 1.21 |
| E | | AJ490336 | 1607037_at | CAD35400 | Shaker-like potassium channel (Vv) | | 0.48 | -0.02 | -0.69 |
| *Sulfate* | |  |  |  |  | |  |  |  |
| F | TC47443 | | 1620065_at | CAG17932 | Plasma membrane sulphate transporter | -3.61 | | 0.09 | 0.89 |
| B | | TC49555 | 1609903_at | BAA75015 | Sulfate transporter | | -1.29 | -1.45 | 1.15 |
| E | | TC48754 | 1608852_at | BAB21264 | Sulfate transporter Sultr3 | | 0.88 | -0.13 | -2.05 |
| *Phosphate* | | |  |  |  | |  |  |  |
| L | TC42012 | | 1621016_at | NP_197538 | Phosphate transporter | -0.13 | | 0.56 | -0.7 |
| C | | TC40312 | 1620378_at | AAP78931 | Na+-dependent inorganic phosphate cotransporter | | -0.34 | 0.82 | -1.15 |
| J | | TC47178 | 1607681_at | AAF74025 | Inorganic phosphate transporter | | -3.63 | -1.02 | 1.28 |
| *Malate* | |  |  |  |  | |  |  |  |
| J | TC41552 | | 1614820_at | BAA96091 | Sodium-dicarboxylate cotransporter | -3.54 | | -2.12 | 1.42 |
| L | | TC45835 | 1614834_at | CAD89675 | Vacuolar pyrophosphatase (Vv) | | 0.27 | 0.61 | -1.92 |
| *ABC transporters* | | | | | | | | | |
| F | TC42025 | | 1612744_at | AAP80385 | ABC transporter WBC11 | -4.54 | | 1.26 | -0.82 |
| F | | TC43702 | 1611325_at | AAP80385 | ABC transporter WBC11 | | -4.79 | 1.26 | -0.83 |
| C | | TC48111 | 1609973_at | XP_450986 | ABC transporter WBC11 | | -1.71 | 1.28 | -1.93 |
| K | | TC48713 | 1608408_at | AAL06532 | ABC transporterWBC15 | | -4.68 | 1.41 | -1.74 |
| J | | TC49764 | 1607632_at | NP_175745 | ABC transporter WBC25 | | -1.47 | -0.45 | 0.93 |
| G | | TC40406 | 1619718_at | AAM67104 | ABC transporter WBC13 | | 0.84 | -0.5 | -0.98 |
| F | | TC47027 | 1608714_at | AAD10836 | ABC transporter MDR1 | | -1.41 | 0.7 | 0.01 |
| J | | TC51669 | 1612678_at | AAO50638 | ABC transporter NAP3 | | -1.32 | -0.4 | 0.88 |
| L | | TC49064 | 1622620_at | CAB41856 | ABC-type transport ATH1 | | -0.04 | 0.7 | -1.3 |
| F | | TC40515 | 1619309_at | XP_468331 | ABC transporter NAP2 /GNC | | -0.64 | 0.49 | -0.07 |
| K | | CF415447 | 1615598_at | AAC32236 | ABC transporter PRD | | 0.61 | 0.58 | -0.24 |
| F | | TC38474 | 1611167_at | AAL08291 | ABC transporter GCN | | -0.72 | 0.17 | 0.34 |
| *Sugar* | |  |  |  |  | |  |  |  |
| L | TC41130 | | 1614764_at | AAT77693 | Hexose transporter HT2 (Vv) | 0.24 | | 0.37 | -0.94 |
| E | | TC39483 | 1619691_at | AAM19835 | Sugar transporter protein HT6 | | 0.54 | 0.26 | -1.52 |
| E | | TC44413 | 1615257_at | AAY40466 | Hexose transporter (Vv) | | 0.59 | 0.2 | -1.5 |
| E | | TC49698 | 1617169_at | AAL79578 | Organic cation transporter | | 0.75 | 0.16 | -2.34 |
| F | | TC47882 | 1620670_at | AAA79769 | Sugar carrier protein HT7 | | -1.46 | 0.07 | 0.67 |
| D | | TC40715 | 1611326_at | CAC00697 | Sugar transporter | | 0.13 | -1.38 | 0.5 |
| B | | TC42302 | 1613408_at | AAB68028 | Sugar transporter | | -1.53 | -2.28 | 1.29 |
| B | | AF021810 | 1615697_at | AAF08331 | Sucrose transporter Suc 27 (Vv) | | -1.61 | -2.14 | 1.29 |
| E | | TC44139 | 1614674_at | AAW82754 | Sucrose-phosphate synthase (Vv) | | 0.64 | 0.43 | -2.97 |

Table 8. Tissue specific expression of mRNA encoding proteins involved in auxin signaling and response. Values are expressed as log ratio from the average expression of mRNA among tissues. Vv indicates a significant match with a *Vitis vinifera* gene.

| **Cluster** | | **Unigene** | | **Affy Probe** | | **Top match N°** | | **Description** | | **Pulp** | | **Skin** | | **Seed** |
| --- | --- | --- | --- | --- | --- | --- | --- | --- | --- | --- | --- | --- | --- | --- |
| Auxin metabolism | | | | | | | | | |  | | | | |
| A | | TC38971 | | 1608579_at | | AAU09443 | | IAA beta-glucosyltransferase | | 0.55 | | -0.57 | | -0.22 |
| E | | TC51523 | | 1615645_at | | AAK97436 | | IAA amidohydrolase | | 0.68 | | 0.27 | | -2.42 |
| F | | TC40938 | | 1622360_at | | BAA11770 | | Nitrilase | | -4.08 | | 0.13 | | 0.88 |
| Auxin regulated | | | | | | | | | | | | | | |
| K | | TC45186 | | 1612090_s_at | | AAD00295 | | Auxin-binding protein ABP19 | | -0.61 | | 0.83 | | -0.83 |
| B | | CA811091 | | 1610989_at | | CAA39709 | | Auxin-induced protein | | -1.73 | | -1.75 | | 1.26 |
| G | | TC42718 | | 1620078_at | | AAC08401 | | Auxin-induced protein | | 0.58 | | -0.19 | | -0.67 |
| E | | TC40686 | | 1609591_at | | AAO63268 | | Auxin-induced protein 10A5. | | 0.54 | | 0.05 | | -0.99 |
| E | | AY082522 | | 1615728_at | | AAL92850 | | Auxin-induced protein IAA9 | | 0.57 | | 0.22 | | -1.53 |
| K | | TC47591 | | 1616717_at | | CAC84712 | | Aux/IAA protein | | -3.42 | | 1.46 | | -2.65 |
| E | | TC46029 | | 1618006_at | | AAL92850 | | Aux/IAA protein (Vv) | | 0.36 | | 0.15 | | -0.73 |
| E | | TC39319 | | 1619395_at | | CAD30274 | | IAA16 | | 0.49 | | 0.23 | | -1.23 |
| G | | TC48647 | | 1615321_at | | AAC49054 | | IAA13 | | 0.8 | | -0.23 | | -1.32 |
| G | | TC46080 | | 1620512_at | | AAC99773 | | IAA27 | | 0.95 | | -0.36 | | -1.76 |
| K | | TC40543 | | 1613054_at | | CAA18855 | | Auxin-regulated protein | | -1.64 | | 1.2 | | -1.37 |
| C | | TC39108 | | 1622100_at | | AAO63268 | | Auxin-regulated protein | | -0.31 | | 0.73 | | -0.91 |
| L | | TC39980 | | 1620726_at | | AAK96798 | | Auxin-regulated protein | | -0.08 | | 0.47 | | -0.59 |
| L | | TC46740 | | 1616104_at | | CAA18855 | | Auxin-regulated protein | | 0.2 | | 0.71 | | -2.22 |
| C | | TC45774 | | 1610591_at | | AAT47070 | | Auxin-regulated protein | | -0.46 | | 0.77 | | -0.8 |
| J | | TC38370 | | 1607503_s_at | | AAC62104 | | Auxin-repressed protein | | -1.91 | | -0.78 | | 1.11 |
| I | | CF371851 | | 1619658_at | | CAB38206 | | Auxin-responsive GH3 | | -0.25 | | -0.79 | | 0.66 |
| L | | TC41864 | | 1621201_at | | BAB02503 | | Auxin-responsive protein | | -0.28 | | 0.79 | | -1.16 |
| E | | TC40469 | | 1620224_at | | AAM65781 | | Auxin-responsive protein | | 0.51 | | 0.44 | | -2.17 |
| E | | TC46727 | | 1613717_a_at | | AAM65781 | | Auxin-responsive protein | | 0.58 | | 0.3 | | -1.86 |
| E | TC47268 | | 1619741_at | | AAM21317 | | Auxin-regulated protein | | 0.58 | | 0.18 | | -1.43 | |
| E | | TC42737 | | 1619751_at | | BAA20848 | | Aux22d | | 0.59 | | 0.22 | | -1.61 |
| L | | TC45938 | | 1614660_at | | AAA33944 | | Auxin-regulated protein (Aux22) | | 0.26 | | 0.46 | | -1.24 |
| L | | TC38800 | | 1613468_at | | AAD32146 | | Nt-iaa28 deduced protein | | -0.31 | | 0.98 | | -2.18 |
| C | | TC41908 | | 1616785_at | | AAQ74955 | | Gbiaa-Re | | -0.42 | | 0.79 | | -0.95 |
| I | | TC48926 | | 1616321_at | | NP_182046 | | Auxin-responsive protein-related | | -0.12 | | -0.83 | | 0.6 |
| F | | TC39930 | | 1622745_at | | CAD31838 | | Quinone oxidoreductase | | -0.92 | | 0.59 | | -0.06 |
| I | | TC50123 | | 1608477_at | | AAL13435 | | Ring box-1 protein | | -0.17 | | -0.6 | | 0.54 |
| Auxin transport | | | | | | | | | | | | | | |
| E | | TC48033 | | 1612060_at | | BAC98948 | | AUX1 auxin influx carrier | | 0.81 | | 0.15 | | -2.77 |
| E | | TC42135 | | 1614098_at | | CAC12996 | | AUX1permease | | 0.81 | | 0.15 | | -2.89 |
| J | | TC40827 | | 1617097_at | | NP_683316 | | Auxin efflux carrier | | -0.72 | | -0.4 | | 0.71 |
| L | | TC45718 | | 1617163_at | | NP_201399 | | Auxin efflux carrier | | 0.09 | | 0.43 | | -0.78 |
| K | | CF405646 | | 1620574_s_at | | BAC41319 | | PIN1-like auxin transport | | -2.47 | | 1.35 | | -1.88 |
| G | | TC50194 | | 1621946_at | | BAC41319 | | PIN1-like auxin transport protein | | 0.94 | | -0.35 | | -1.78 |
| C | | TC41939 | | 1611491_at | | AAM54033 | | PIN1-like auxin transport protein | | -0.43 | | 0.78 | | -0.9 |
| Auxin response factor | | | | | | | | | | | | | | |
| E | TC39051 | | 1606826_at | | AAP06759 | | Auxin response factor protein | | 0.59 | | 0.03 | | -1.08 | |
| L | | TC41676 | | 1616015_at | | AAC49751 | | Auxin response factor 1 | | 0.23 | | 0.56 | | -1.48 |
| E | | TC39051 | | 1606826_at | | AAP06759 | | Auxin response factor | | 0.59 | | 0.03 | | -1.08 |

Table 9. Tissue specific expression of mRNA encoding proteins involved in ABA biosynthesis, signaling and response. Values are expressed as log ratio from the average expression of mRNA among tissues. Vv indicates a significant match with a *Vitis vinifera* gene.

| **Cluster** | **Unigene** | **Affy Probe** | **Top match N°** | **Description** | **Pulp** | **Skin** | **Seed** |
| --- | --- | --- | --- | --- | --- | --- | --- |
| Abscisic acid biosynthesis | | | | | | | |
| G | TC43255 | 1610455_at | CAB07784 | Nine-cis-epoxycarotenoid dioxygenase | 1.07 | -0.51 | -2.31 |
| G | TC42536 | 1607029_at | CAC34025 | Nine-cis-epoxycarotenoid dioxygenase | 1.02 | -0.57 | -1.72 |
| G | TC48377 | 1608022_at | AAR11193 | Nine-cis-epoxycarotenoid dioxygenase (Vv) | 0.52 | -0.07 | -0.71 |
| Abscisic acid regulated | | | | | | | |
| E | CB982969 | 1619261_s_at | AAD31879 | ABA-responsive protein (HVA22a) | 0.42 | 0.4 | -1.54 |
| F | TC38296 | 1616426_at | AAQ22345 | BURP domain-containing protein | -5.86 | 1.17 | -0.46 |
| B | CB982875 | 1619802_at | BAA92225 | Similar to the BURP domain | -6.53 | -6.94 | 1.58 |
| B | TC41158 | 1620890_at | AAB39474 | Em protein (LEA protein) | -5.99 | -6.17 | 1.57 |
| K | TC38295 | 1621818_at | AAL26909 | Dehydration-responsive protein RD22 | -1.15 | 1.06 | -1.12 |
| F | TC38630 | 1619164_at | AAL67991 | Dehydration-induced protein RD22 | -0.94 | 0.67 | -0.18 |
| E | TC47836 | 1614372_at | CAC08335 | ABA-responsive protein | 0.72 | 0.11 | -1.86 |
| E | TC41345 | 1609270_at | AAM93433 | ACR8 | 0.6 | 0.41 | -2.69 |
| Abscisic acid signaling | | | | | | | |
| E | TC41302 | 1617441_at | AAO42812 | Calcium-dependent protein kinase ATCDPK1 | 0.52 | 0.35 | -1.77 |
| L | TC49237 | 1612443_at | NP_913237 | CBL-interacting protein kinase 20 (CIPK20) | -0.19 | 0.83 | -1.54 |
| L | TC50832 | 1618045_at | AAL23677 | Serine\/threonine Kinase | 0.21 | 0.47 | -1.12 |
| J | TC44877 | 1610938_at | AAG31326 | Serine\/threonine kinase GDBrPK (Vv) | -0.6 | -0.08 | 0.48 |
| E | TC50504 | 1607672_at | AAK92818 | ABI1 protein phosphatase | 0.54 | 0.32 | -1.75 |
| Abscisic acid response factors | | | | | | | |
| I | TC50572 | 1608885_at | XP_479538 | VP1\/ABI3 family regulatory protein | -0.66 | -1.22 | 0.95 |
| B | TC40343 | 1621346_at | CAA05921 | ABI3 | -4.59 | -4.79 | 1.55 |
| C | TC39047 | 1621556_s_at | AAF01764 | Homeodomain-leucine zipper | -0.25 | 0.65 | -0.78 |

Table 10. Tissue specific expression of mRNA encoding proteins involved in gibberellin biosynthesis, signaling and response. Values are expressed as log ratio from the average expression of mRNA among tissues. Vv indicates a significant match with a *Vitis vinifera* gene.

| **Cluster** | **Unigene** | **Affy Probe** | **Top match N°** | **Description** | **Pulp** | **Skin** | **Seed** |
| --- | --- | --- | --- | --- | --- | --- | --- |
| B | TC38517 | 1609893_at | AAB62947 | GAST1 | -4.38 | -4.93 | 1.55 |
| E | TC39759 | 1621228_at | AAR31213 | GAI protein | 0.51 | 0.46 | -2.31 |
| B | AY256862 | 1621507_s_at | AAP20096 | GAI1 (Vv) | -1.16 | -0.93 | 1.02 |
| B | AF378125 | 1606777_s_at | AAM19210 | GAI-like protein 1 (Vv) | -0.77 | -0.81 | 0.88 |
| G | TC49189 | 1618181_at | AAM96971 | AtGID1a | 0.62 | -0.35 | -0.57 |
| K | TC40495 | 1610607_at | CAD35176 | GASA4 | -0.62 | 0.61 | -0.29 |
| F | TC42201 | 1612627_at | AAQ93035 | Gibberellin 2-oxidase | -1.4 | 0.71 | -0.02 |
| I | TC47765 | 1618503_at | BAD17855 | Gibberellin 2-oxidase 1 | -0.26 | -0.66 | 0.61 |
| I | TC45582 | 1622414_at | AAT77035 | Oxidoreductase | -1.53 | -2.64 | 1.32 |
| E | TC46488 | 1618081_at | BAD72256 | Oxidoreductase | 0.38 | 0.26 | -0.99 |
| I | TC39970 | 1609749_at | AAG52269 | Oxidoreductase | -2.02 | -3.41 | 1.41 |
| E | TC45125 | 1606879_at | CAC44032 | Snakin-1 | 0.3 | 0.2 | -0.7 |
| G | TC45128 | 1617501_at | CAC44032 | Snakin-1 | 0.47 | -0.05 | -0.63 |

Table 11. Tissue specific expression of mRNA encoding proteins involved in jasmonate biosynthesis, signaling and response. Values are expressed as log ratio from the average expression of mRNA among tissues. Vv indicates a significant match with a *Vitis vinifera* gene.

| **Cluster** | **Unigene** | **Affy Probe** | **Top match N°** | **Description** | **Pulp** | **Skin** | **Seed** |
| --- | --- | --- | --- | --- | --- | --- | --- |
| L | TC40697 | 1616418_at | AAF44708 | Wound-inducible carboxypeptidase | 0.4 | 0.51 | -1.99 |
| E | TC38851 | 1615500_at | AAR82925 | Coronatine-insensitive 1 | 0.4 | 0.24 | -1 |
| B | TC39120 | 1609172_at | AAF04915 | Jasmonic acid 2 | -0.42 | -0.56 | 0.66 |
| E | TC46498 | 1615579_at | AAA33021 | Stearoyl-acyl-carrier protein desaturase | 0.4 | 0.35 | -1.3 |
| L | TC46783 | 1613353_at | AAN65180 | Mitogen-activated protein kinase 4 | -0.12 | 0.47 | -0.53 |
| L | TC43235 | 1617751_s_at | AAP83137 | Lipoxygenase | 0.11 | 0.74 | -1.99 |
| L | TC44915 | 1608892_at | AAP83137 | Lipoxygenase | 0.09 | 0.76 | -2.03 |
| L | CF405309 | 1617922_at | AAB65767 | Lipoxygenase D | -0.1 | 0.51 | -0.63 |
| E | TC49699 | 1612190_at | AAO72741 | Allene oxide synthase | 0.31 | 0.24 | -0.79 |
| J | TC41392 | 1619407_s_at | AAC78441 | 12-oxophytodienoate reductase OPR2 | -1.14 | -0.28 | 0.78 |
| B | TC46796 | 1610465_s_at | AAQ93070 | 3-ketoacyl-CoA thiolase | -1.34 | -1.67 | 1.19 |
| F | TC47275 | 1616227_at | AAD38147 | Desacetoxyvindoline 4-hydroxylase | -2.65 | -0.53 | 1.1 |
| J | TC47274 | 1622045_at | AAD38147 | Iron/Ascorbate oxidoreductase f | -1.83 | -0.58 | 1.04 |

Table 12. Tissue specific expression of mRNA encoding proteins involved in ethylene biosynthesis, signaling and response. Values are expressed as log ratio from the average expression of mRNA among tissues. Vv indicates a significant match with a *Vitis vinifera* gene.

| **Cluster** | **Unigene** | **Affy Probe** | **Top match N°** | **Description** | **Pulp** | **Skin** | **Seed** |
| --- | --- | --- | --- | --- | --- | --- | --- |
| Ethylene biosynthesis | | | | | | | |
| L | TC38334 | 1614836_s_at | CAA95856 | S-adenosyl-L-methionine synthetase 1 | -0.09 | 0.62 | -0.93 |
| L | TC38332 | 1607434_s_at | CAA95857 | S-adenosyl-L-methionine synthetase 2 | 0.28 | 0.51 | -1.49 |
| L | TC45908 | 1622147_at | AAA33697 | 1-aminocyclopropane-1-carboxylate oxidase | -0.1 | 0.49 | -0.6 |
| C | TC45908 | 1609995_s_at | AAA33697 | 1-aminocyclopropane-1-carboxylate oxidase | -0.35 | 0.65 | -0.63 |
| E | TC40150 | 1606941_at | CAB77753 | Ethylene-overproduction protein 1 | 0.4 | 0.18 | -0.87 |
| Ethylene signaling | | | | | | | |
| J | TC48190 | 1611310_at | CAB45083 | Protein kinase CTR1 | -2.13 | -1.31 | 1.24 |
| C | CD801620 | 1614827_at | AAR28378 | EIX receptor 2 | -0.78 | 0.98 | -1.16 |
| L | TC50911 | 1618518_at | AAD31397 | Ethylene receptor | 0.21 | 0.33 | -0.78 |
| E | TC39880 | 1611720_at | AAL60026 | EIN3-binding F-box protein | 0.46 | 0.24 | -1.15 |
| E | TC40020 | 1606439_s_at | AAL60026 | EIN3-binding F-box protein | 0.38 | 0.17 | -0.81 |
| Ethylene response | | | | | | | |
| E | TC40246 | 1611583_at | AAM63446 | Ap2/EREBP transcription factor | 0.44 | 0.45 | -1.84 |
| E | TC43562 | 1609559_at | AAK74017 | Ethylene-responsive element protein | 0.4 | 0.2 | -0.89 |
| L | TC45046 | 1617012_at | AAK74017 | Ethylene-responsive element | 0.21 | 0.61 | -1.67 |
| L | TC48122 | 1621745_at | AAL07027 | AP2/EREBP-like transcription factor | -0.5 | 0.94 | -1.4 |
| L | TC47351 | 1613177_at | AAM91103 | Ethylene-induced calmodulin-binding | 0.15 | 0.34 | -0.69 |
| L | TC47621 | 1609780_at | NP_918652 | ER6 protein | -0.01 | 0.51 | -0.77 |
| K | TC45177 | 1619178_at | NP_918652 | ER6 protein | -0.6 | 0.7 | -0.49 |
| C | TC39576 | 1618661_s_at | AAR37422 | Ethylene response factor 4 (va) | -1.28 | 1.14 | -1.4 |
| F | TC41585 | 1608511_at | AAR37423 | Ethylene response factor 5 (va) | -1.86 | 0.94 | -0.32 |
| B | AY395745 | 1611910_s_at | AAQ96342 | Ethylene response factor ERF3b (va) | -2.96 | -3.14 | 1.46 |
| B | TC39828 | 1609990_at | AAQ96342 | Ethylene response factor ERF3b (va) | -3.34 | -2.94 | 1.47 |
| B | TC45815 | 1621552_at | AAO44027 | Ethylene-responsive transcriptional coactivator, | -1.95 | -2.74 | 1.37 |
| G | CF215236 | 1613123_at | CAB43924 | Ethylene-responsive protein | 1.05 | -0.93 | -1.32 |
| J | TC47273 | 1616198_at | BAA97123 | Ethylene-responsive element binding | -1.88 | -0.45 | 1 |
| J | TC39567 | 1611124_at | AAA91063 | Ethylene-inducible protein | -0.64 | -0.07 | 0.49 |

Table 13. Tissue specific expression of mRNA encoding transcription factors. Values are expressed as log ratio from the average expression of mRNA among tissues. Vv indicates a significant match with a *Vitis vinifera* gene.

| **Cluster** | **Unigene** | | **Affy Probe** | **Top match N°** | | **Description** | **Pulp** | **Skin** | **Seed** |
| --- | --- | --- | --- | --- | --- | --- | --- | --- | --- |
| *Basic-helix-loop-helix transcription factors* | | | | | | | | | |
| B | | TC43519 | 1617411_at | | AAM63723 | BHLH transcription factor (bHLH027) | -2.93 | -2.07 | 1.4 |
| B | | TC41944 | 1612317_at | | CAB78042 | BHLH transcription factor (bHLH081) | -1.33 | -1.41 | 1.15 |
| B | | TC45246 | 1619012_s_at | | AAK96776 | BHLH transcription factor (bHLH105) | -0.83 | -0.67 | 0.86 |
| D | | TC51631 | 1611963_at | | AAP40407 | BHLH transcription factor (bHLH123) | 0.28 | -0.73 | 0.24 |
| E | | TC41749 | 1610882_s_at | | CAB43924 | BHLH transcription factor | 1.07 | -0.47 | -2.5 |
| E | | TC46396 | 1608066_at | | AAL32534 | BHLH transcription factor | 0.53 | 0.25 | -1.43 |
| E | | TC45556 | 1608432_s_at | | AAD56411 | BHLH transcription factor GBOF-1 | 0.48 | 0.33 | -1.54 |
| E | | TC40540 | 1620083_at | | AAG25728 | BZIP protein BZO2H2 | 0.38 | 0.24 | -0.96 |
| G | | TC41419 | 1609484_at | | XP_467243 | Basic-helix-loop-helix transcription factor | 0.89 | -0.68 | -0.92 |
| L | | TC48780 | 1608936_at | | NP_181843 | Basic helix-loop-helix (bHLH) family | -0.13 | 0.48 | -0.53 |
| *General transcription factors* | | | | | | | | | |
| B | | TC40987 | 1617362_at | | CAB10233 | CCAAT-binding transcription factor | -1.02 | -1.15 | 1.04 |
| B | | TC43487 | 1620399_at | | AAK62402 | CCAAT-binding transcription factor ( | -0.53 | -0.55 | 0.7 |
| B | | CB346385 | 1618496_at | | BAD15086 | CCAAT-box binding factor HAP2 | -1.23 | -0.85 | 1.01 |
| L | | TC39920 | 1620587_at | | AAM83224 | CCAAT-binding transcription factor | 0.26 | 0.28 | -0.78 |
| G | | TC43833 | 1609970_at | | NP_188898 | RNA pol II accessory factor Cdc73 family | 0.45 | -0.06 | -0.57 |
| J | | TC41471 | 1606707_at | | AAL73491 | General transcription factor TFIIB | -1.71 | -0.8 | 1.08 |
| J | | BQ792898 | 1618351_at | | AAR23731 | TBP-Binding protein | -0.61 | -0.41 | 0.67 |
| L | | TC43409 | 1606781_at | | BAD68649 | TAF14b | 0.1 | 0.55 | -1.1 |
| E | | TC48692 | 1609494_at | | NP_568218 | Transcription elongation factor | 0.43 | 0.13 | -0.84 |
| *Homeobox domain transcription factors* | | | | | | | | | |
| L | | TC41982 | 1616863_at | | AAN03626 | BEL1-related homeotic protein 29 [ | -0.1 | 0.66 | -1.04 |
| L | | TC38709 | 1617742_at | | AAN03626 | BEL1-related homeotic protein 29 [ | 0.25 | 0.5 | -1.32 |
| L | | TC38709 | 1619258_at | | AAN03626 | BEL1-related homeotic protein 29 | 0.24 | 0.59 | -1.7 |
| C | | TC40589 | 1607284_at | | AAP47023 | Bell-like homeodomain protein 3 | -0.18 | 0.52 | -0.56 |
| C | | TC41610 | 1618408_at | | AAM67191 | Helix-loop-helix protein homolog | -2.28 | 1.39 | -2.47 |
| E | | TC46371 | 1607494_at | | AAD00253 | Homeobox 2 protein | 0.33 | 0.29 | -0.95 |
| E | | TC49145 | 1617592_at | | AAD00253 | Homeobox 2 protein | 0.41 | 0.36 | -1.35 |
| G | | TC51295 | 1607122_at | | AAA56902 | Homeobox protein | 0.51 | -0.07 | -0.69 |
| J | | TC42789 | 1610494_at | | AAK59762 | homeobox protein | -1.42 | -0.15 | 0.79 |
| F | | TC40185 | 1617931_at | | CAB71896 | Homeobox-leucine zipper protein ATHB-12 | -1.72 | 0.31 | 0.54 |
| F | | TC47331 | 1618437_at | | AAK84886 | Homeodomain leucine zipper protein HDZ2 | -2.02 | 0.71 | 0.17 |
| K | | TC47908 | 1614235_at | | AAC79430 | Homeodomain protein | -3.41 | 1.4 | -1.95 |
| L | | TC41356 | 1615068_at | | CAB82976 | Homeodomain protein | 0.02 | 0.74 | -1.68 |
| L | | TC40127 | 1606639_at | | AAM97321 | Homeodomain protein GhHOX1 | 0.11 | 0.66 | -1.55 |
| L | | TC49946 | 1613196_at | | AAM97321 | Homeodomain protein GhHOX1 | 0.23 | 0.61 | -1.72 |
| K | | TC42148 | 1608182_at | | BAD73349 | HB2 homeodomain protein | -0.68 | 0.78 | -0.59 |
| *Miscellaneous transcription factors* | | | | | | | | | |
| K | | TC39603 | 1612525_at | | BAD62387 | 41 kD chloroplast nucleoid DNA binding | -2.57 | 1.33 | -1.62 |
| E | | TC40422 | 1617821_at | | AAG10141 | Activator subunit of SNF1 protein kinase | 0.42 | 0.05 | -0.66 |
| J | | TC49466 | 1609265_at | | BAC98494 | AG-motif binding protein-4 | -0.82 | 0.03 | 0.5 |
| L | | TC41479 | 1620641_at | | BAA08094 | Ascorbate oxidase promoter-binding protein | -0.02 | 0.63 | -1.1 |
| G | | TC46710 | 1620278_at | | NP_173638 | AP2 domain-containing transcription factor | 0.7 | -0.11 | -1.16 |
| J | | TC42225 | 1620170_at | | AAD22495 | APETALA2 protein homolog HAP2 | -0.65 | 0 | 0.45 |
| J | | TC49547 | 1621975_at | | BAD10062 | AT-hook DNA-binding protein | -1.12 | -0.06 | 0.66 |
| L | | TC47104 | 1622296_at | | AAN03468 | BZIP transcription factor ATB2 | 0.35 | 0.64 | -2.58 |
| E | | TC38784 | 1617396_at | | AAN03468 | BZIP transcription factor ATB2 | 0.43 | 0.36 | -1.45 |
| F | | TC45535 | 1622598_at | | CAB88994 | CCR4-associated factor 1 protein | -0.95 | 0.69 | -0.21 |
| K | | TC39604 | 1619775_at | | BAD12879 | Chloroplast nucleoid DNA-binding protein | -2.53 | 1.3 | -1.47 |
| E | | TC42490 | 1622845_at | | AAX19050 | Class III HD-Zip protein 1 | 0.33 | 0.17 | -0.7 |
| F | | TC40814 | 1618763_at | | CAC00657 | Common plant regulatory factor 6 | -0.8 | 0.5 | 0.02 |
| B | | TC47858 | 1621461_at | | AAB62864 | Contains region of similarity to SYT | -0.39 | -0.56 | 0.64 |
| B | | TC48912 | 1615970_at | | AAQ08000 | Dehydration responsive element binding | -0.65 | -0.8 | 0.84 |
| E | | TC45892 | 1621314_s_at | | AAO13360 | Dehydration-responsive element binding | 0.49 | 0.2 | -1.16 |
| J | | TC41529 | 1616745_at | | NP_187109 | DNA-binding family | -0.63 | 0.05 | 0.4 |
| L | | TC45685 | 1613097_at | | CAB71056 | DNA-binding protein | 0.1 | 0.5 | -0.97 |
| K | | TC40283 | 1610309_at | | AAK62408 | DNA-binding protein DF1 | -1.23 | 0.96 | -0.66 |
| E | | TC47722 | 1615083_at | | CAC36939 | DOF zinc finger protein | 0.44 | 0.07 | -0.74 |
| E | | TC47722 | 1611313_at | | CAC36939 | DOF zinc finger like protein | 0.9 | -0.01 | -2.87 |
| E | | TC41846 | 1608526_at | | AAO72549 | Dof zinc finger protein DOF3.3 (AtDOF3.3) | 0.48 | -0.01 | -0.69 |
| B | | TC40797 | 1620672_at | | NP_177685 | Forkhead-associated domain-containing protein | -0.58 | -0.5 | 0.7 |
| B | | TC40824 | 1620877_at | | CAB81839 | GATA transcription factor 4 | -0.61 | -0.77 | 0.81 |
| B | | TC47818 | 1615028_at | | CAA74000 | GATA-binding transcription factors | -2.12 | -1.98 | 1.33 |
| J | | TC49539 | 1611070_at | | AAO22666 | GRF1-interacting factor 1 | -1.22 | -0.6 | 0.93 |
| E | | TC47678 | 1612310_at | | BAA83710 | Heat shock factor | 0.69 | -0.07 | -1.19 |
| B | | TC38764 | 1610122_at | | CAA47870 | Heat stress transcription factor HSF30 | -0.84 | -0.87 | 0.92 |
| I | | TC38606 | 1609060_s_at | | AAL36161 | Heavy-metal-associated domain-containing | -0.2 | -0.52 | 0.52 |
| L | | TC41034 | 1620384_s_at | | NP_568436 | Heavy-metal-associated domain-containing | -0.04 | 0.59 | -0.95 |
| A | | CF206451 | 1610949_s_at | | NP_171656 | Heavy-metal-associated domain-containing | 0.59 | -0.59 | -0.26 |
| J | | TC40397 | 1606534_at | | AAP40501 | Leucine zipper protein | -1.22 | -0.07 | 0.69 |
| C | | TC38635 | 1620850_at | | AAR20445 | Leucine zipper protein | -1.88 | 1.36 | -2.67 |
| E | | TC45891 | 1620717_at | | AAM19847 | Leucine zipper-containing protein | 0.44 | 0.06 | -0.73 |
| G | | TC49667 | 1613418_at | | AAM19847 | Leucine zipper-containing protein | 0.52 | -0.06 | -0.71 |
| J | | TC50775 | 1612939_at | | BAD38193 | Mitochondrial transcription termination factor | -1.14 | -0.7 | 0.95 |
| B | | CF414688 | 1622718_at | | NP_173539 | Mitochondrial transcription termination factor | -0.27 | -0.51 | 0.55 |
| B | | TC40783 | 1613366_at | | AAO43102 | Mutant cincinnata | -0.37 | -0.57 | 0.64 |
| B | | TC49348 | 1614909_at | | AAQ14331 | MYC1 | -0.85 | -0.86 | 0.92 |
| L | | TC48362 | 1618504_at | | AAS86309 | myc anthocyanin regulatory protein | 0.05 | 0.39 | -0.62 |
| K | | TC42005 | 1610480_at | | AAK84883 | NAC domain protein NAC1 | -0.79 | 0.89 | -0.81 |
| K | | TC43527 | 1613141_at | | AAD22369 | NAM (no apical meristem | -0.91 | 0.98 | -1.04 |
| E | | TC40193 | 1611709_at | | AAK43925 | PCF2-like DNA binding protein | 0.47 | 0.07 | -0.83 |
| C | | TC43656 | 1614065_at | | AAD40953 | Phantastica | -0.22 | 0.57 | -0.6 |
| B | | TC51247 | 1621339_at | | BAB02165 | Photomorphogenesis repressor protein | -1.63 | -1.07 | 1.14 |
| G | | TC46080 | 1620512_at | | AAC99773 | Phytochrome-associated protein | 0.95 | -0.36 | -1.76 |
| L | | TC42554 | 1616581_at | | NP_850381 | Phytochrome-interacting factor 4 | 0.34 | 0.57 | -1.96 |
| E | | TC38893 | 1614418_at | | AAC13695 | PTD protein | 0.67 | 0.2 | -1.98 |
| K | | TC41747 | 1614735_at | | NP_915631 | DNA-binding protein | -0.71 | 0.83 | -0.7 |
| B | | TC43291 | 1609745_at | | AAC49474 | Regulator of MAT2 | -0.52 | -0.74 | 0.77 |
| E | | TC38913 | 1614779_s_at | | BAC79688 | Remorin | 0.36 | 0.16 | -0.74 |
| L | | TC44645 | 1621823_at | | CAB72174 | Response reactor 4 | 0.23 | 0.35 | -0.87 |
| E | | TC41348 | 1608140_at | | BAA34728 | Response regulator 6 | 0.52 | -0.03 | -0.78 |
| E | | TC47902 | 1613746_at | | AAC19296 | Scarecrow | 0.57 | 0.29 | -1.76 |
| B | | TC47632 | 1619334_at | | AAL32616 | SCARECROW gene regulator | -1.02 | -0.63 | 0.9 |
| E | | TC38975 | 1609074_at | | BAB10182 | SCARECROW transcriptional regulator | 0.36 | 0.22 | -0.86 |
| C | | TC40277 | 1613748_at | | AAP83390 | SEPALLATA1 MADS-box | -0.4 | 0.81 | -1.03 |
| L | | TC45888 | 1617580_at | | CAA77213 | Sigma factor | -0.08 | 0.56 | -0.78 |
| F | | TC42854 | 1619987_at | | BAA88922 | Sigma factor | -0.89 | 0.47 | 0.1 |
| B | | TC44421 | 1619830_at | | CAB56768 | Squamosa promoter binding protein | -0.68 | -0.91 | 0.88 |
| F | | TC50229 | 1620576_at | | BAD54038 | Squamosa promoter binding protein | -0.93 | 0.32 | 0.3 |
| L | | TC50043 | 1621951_at | | AAM63137 | TINY-like protein | 0.04 | 0.57 | -1.03 |
| B | | TC50340 | 1610171_at | | AAF14044 | Transcription factor | -0.43 | -0.4 | 0.59 |
| E | | TC42176 | 1613776_at | | AAO14628 | Transcription factor monopteros | 0.54 | -0.02 | -0.84 |
| L | | TC40974 | 1622859_at | | AAQ10678 | Type-B response regulator | 0.26 | 0.27 | -0.74 |
| E | | TC41404 | 1614298_at | | AAS10179 | YABBY2-like transcription factor YAB2 | 1.02 | -0.36 | -2.39 |
| L | | BQ797739 | 1618219_at | | AAD33717 | YABBY3 | -0.33 | 0.95 | -1.84 |
| *MADS box transcription factors* | | | | | | | | | |
| E | | AF265562 | 1619742_at | | AAK58564 | MAD-box transcripion factor (Vv) | 0.42 | 0.24 | -1.07 |
| E | | TC39943 | 1619977_at | | AAF22455 | MADS box protein | 0.48 | 0.23 | -1.23 |
| G | | TC41683 | 1611361_at | | AAQ83693 | MADS-box protein | 0.61 | -0.17 | -0.77 |
| G | | AF373601 | 1615428_at | | AAM21342 | MADS-box protein 2 (Vv) | 0.53 | -0.14 | -0.62 |
| G | | AF373602 | 1612606_at | | AAM21343 | MADS-box protein 3 (Vv) | 0.71 | -0.22 | -0.97 |
| B | | AF373604 | 1621836_at | | AAM21345 | MADS-box protein 5 (Vv) | -1.67 | -2.13 | 1.3 |
| L | | TC38620 | 1607973_at | | AAD03486 | MADS1 | 0.29 | 0.29 | -0.84 |
| *Myb transcription factors* | | | | | | | | | |
| C | | TC51437 | 1618514_at | | BAA21917 | MYB transcription factor | -0.79 | 0.98 | -1.18 |
| F | | TC39225 | 1614953_at | | AAB23528 | Myb oncoprotein | -1.99 | 0.61 | 0.29 |
| G | | TC41451 | 1618968_at | | NP_974216 | Myb family transcription | 0.64 | -0.28 | -0.69 |
| G | | TC48806 | 1614932_at | | CAI30890 | MYB transcription factor | 0.56 | -0.2 | -0.62 |
| L | | TC40617 | 1614416_at | | AAL78741 | MYB transcription factor DIVARICATA | 0.41 | 0.6 | -2.69 |
| L | | TC45686 | 1613486_at | | AAP21221 | MYB transcription factor | 0.05 | 0.8 | -2.18 |
| L | | TC45624 | 1617998_at | | AAN28287 | Myb transcription factor 6 | -0.78 | 1.21 | -3.22 |
| L | | TC49276 | 1613239_at | | AAN72013 | Myb transcription factor | 0.31 | 0.52 | -1.64 |
| I | | TC46393 | 1616094_at | | AAA82943 | MYB-like transcriptional factor MBF1 | -0.77 | -1.92 | 1.1 |
| I | | AB073014 | 1611091_s_at | | BAC07541 | Myb-related transcription factor VlMYBC (Vv) | -0.87 | -2.09 | 1.15 |
| I | | TC46978 | 1613318_at | | BAC53938 | MYB transcription factor | -0.18 | -0.81 | 0.63 |
| J | | TC39707 | 1614931_at | | AAB63650 | Myb-related transcription activator (MybSt1) | -1 | -0.41 | 0.81 |
| K | | AB073013 | 1615798_at | | BAC07540 | Myb transcription factor VlMYBA2 (Vv) | -1.25 | 1.09 | -1.15 |
| K | | TC48485 | 1620959_s_at | | BAD18978 | Myb transcription factor VvMYBA2 (Vv) | -4.61 | 1.44 | -2.02 |
| *WRKY transcription factor* | | | | | | | | | |
| C | | TC49651 | 1620022_at | | AAL61859 | WRKY transcription factor 57 | -0.78 | 1.06 | -1.57 |
| L | | TC49418 | 1620175_at | | AAQ57651 | WRKY 13 | -0.62 | 1.11 | -2.42 |
| K | | BQ800205 | 1619424_at | | AAK28441 | WRKY DNA-binding protein 21 | -0.9 | 1.01 | -1.14 |
| K | | TC46595 | 1611550_at | | AAR92477 | WRKY transcription factor 30 (Vv | -0.84 | 0.92 | -0.86 |
| F | | TC45602 | 1610064_at | | BAA06278 | SPF1 protein | -2.14 | -0.27 | 0.96 |
|  | |  |  | |  |  |  |  |  |
| E | | TC46341 | 1609130_at | | AAL35290 | WRKY transcription factor 48 | 0.34 | 0.31 | -1 |
| E | | TC40428 | 1622333_at | | AAO86686 | CaWRKY1 | 0.37 | 0.21 | -0.86 |
| F | | TC46952 | 1611650_at | | BAB16432 | WRKY transcription factor NtEIG-D48 | -0.68 | 0.49 | -0.03 |
| F | | TC40931 | 1616623_at | | AAL13047 | WRKY transcription factor 71 | -1.97 | 0.6 | 0.3 |
| F | | CA809370 | 1612649_s_at | | BAB16432 | WRKY transcription factor NtEIG-D48 | -1.63 | 0.55 | 0.28 |
| J | | TC47383 | 1606659_s_at | | AAT64024 | WRKY transcription factor | -1.24 | -0.12 | 0.73 |
| J | | CA809190 | 1611285_s_at | | CAE03880 | WRKY transcription factor 11 | -1.69 | -0.95 | 1.12 |
| J | | TC39121 | 1614806_s_at | | AAR37421 | WRKY4 transcription factor (Vv) | -0.96 | -0.14 | 0.66 |
| *Zinc finger transcription factors* | | | | | | | | | |
| K | | TC46405 | 1618741_at | | A84720 | B-box zinc finger protein (STH) | -0.55 | 0.78 | -0.75 |
| J | | TC42939 | 1612095_at | | AAK96816 | B-box zinc finger protein | -2.88 | -1.39 | 1.31 |
| I | | TC46819 | 1608942_at | | CAC43563 | C2H2-type zinc finger protein | -0.94 | -2.03 | 1.16 |
| L | | TC43370 | 1614388_at | | AAK73976 | C3HC4 zinc finger | 0.03 | 0.47 | -0.75 |
| E | | TC45839 | 1614469_at | | AAK59832 | CCCH-type zinc finger like protein | 0.41 | 0.31 | -1.23 |
| E | | TC47849 | 1611185_at | | E84847 | CCCH-type zinc finger protein | 0.56 | 0.29 | -1.71 |
| B | | TC40023 | 1613783_at | | AAM97061 | CCCH-type zinc finger protein | -0.66 | -0.78 | 0.84 |
| J | | TC40935 | 1613759_s_at | | AAM97061 | CCCH-type zinc finger protein | -0.99 | -0.21 | 0.71 |
| E | | TC42190 | 1617572_at | | CAB71076 | RING finger protein | 0.55 | 0.13 | -1.18 |
| B | | TC48700 | 1608201_at | | BAD53026 | Ring finger protein 1 | -0.91 | -0.68 | 0.88 |
| B | | TC51306 | 1608644_at | | AAO63320 | RING zinc finger like protein | -1.88 | -1.77 | 1.28 |
| E | | TC41835 | 1619965_at | | AAK76523 | RING zinc finger like protein | 0.38 | 0.13 | -0.74 |
| E | | TC41371 | 1613050_at | | AAF19568 | RING zinc finger protein | 0.36 | 0.28 | -0.99 |
| A | | TC48889 | 1608656_at | | AAM19707 | RING zinc finger protein | 0.57 | -0.51 | -0.31 |
| L | | TC44213 | 1612574_s_at | | BAB10906 | RING zinc finger protein | 0.14 | 0.51 | -1.08 |
| E | | TC50319 | 1620013_at | | BAD25417 | RING zinc finger protein | 0.62 | 0.04 | -1.21 |
| K | | TC40377 | 1606575_at | | AAO44037 | RING zinc finger protein | -0.74 | 0.79 | -0.59 |
| G | | TC40886 | 1619626_at | | AAO41888 | RING-H2 zinc finger protein RHA3a. | 0.71 | -0.27 | -0.89 |
| L | | TC39187 | 1611674_at | | AAO45753 | RING\/C3HC4\/PHD zinc finger | 0.08 | 0.57 | -1.14 |
| L | | TC48541 | 1620607_s_at | | AAC63643 | Zinc finger (B-box type) protein | -0.07 | 0.53 | -0.73 |
| L | | TC47716 | 1606951_at | | AAR24705 | Zinc finger (B-box type) protein | 0.21 | 0.61 | -1.65 |
| E | | TC46692 | 1619251_at | | BAD46368 | Zinc finger (B-box type) protein | 0.49 | 0.36 | -1.71 |
| B | | TC48460 | 1609411_at | | CAB86082 | Zinc finger (C2H2 type) protein | -0.8 | -0.61 | 0.82 |
| F | | TC47318 | 1607067_at | | AAL69457 | Zinc finger (C3HC4-type RING finger) | -1.28 | 0.78 | -0.2 |
| L | | TC48116 | 1611925_at | | BAB08414 | Zinc finger (C3HC4-type RING finger) | 0.24 | 0.45 | -1.15 |
| G | | TC40897 | 1609315_at | | NP_191431 | Zinc finger (C3HC4-type RING finger) family | 1.08 | -0.52 | -2.43 |
| H | | TC41884 | 1621663_at | | NP_189139 | Zinc finger (C3HC4-type RING finger) family | 0.37 | -0.68 | 0.11 |
| E | | TC43720 | 1614534_at | | CAB10287 | Zinc finger (DHHC type) protein | 0.53 | 0.12 | -1.09 |
| E | | TC44293 | 1615704_at | | CAB10287 | Zinc finger (DHHC type) protein | 0.48 | 0.15 | -1 |
| E | | TC46914 | 1611921_at | | AAL32640 | Zinc finger (GATA type) protein | 0.54 | 0.13 | -1.14 |
| J | | TC41320 | 1617518_at | | AAM65531 | Zinc finger protein | -0.59 | -0.03 | 0.44 |
| L | | TC41222 | 1606914_at | | AAG52592 | Zinc finger protein | 0.11 | 0.55 | -1.13 |
| K | | TC41271 | 1607310_at | | AAK92829 | Zinc finger protein | -1.12 | 0.94 | -0.7 |
| L | | TC41234 | 1610633_at | | AAM63627 | Zinc finger protein | -0.26 | 0.94 | -2 |
| E | | TC47511 | 1611684_at | | BAA97520 | Zinc finger protein | 0.42 | 0.01 | -0.62 |
| E | | TC49587 | 1613148_at | | AAK82515 | Zinc finger protein 2 | 0.47 | 0.02 | -0.74 |
| G | | TC48646 | 1613842_at | | BAD37964 | Zinc finger protein ID1 | 0.91 | -0.43 | -1.39 |
| J | | TC48064 | 1613740_at | | AAG51898 | Zinc finger protein | -0.58 | -0.02 | 0.43 |
| J | | TC42987 | 1619173_at | | AAF79306 | Zinc finger protein | -0.61 | -0.29 | 0.61 |
| E | | TC49955 | 1618123_at | | BAA21927 | ZPT3-3 [Petunia x hybrida] | 0.45 | 0.46 | -2 |
