# Supplementary material for: Tissue-specific mRNA expression profiling in grape berry tissues
Source: BMC Genomics. 2007 Jun 21;8:187. doi: 10.1186/1471-2164-8-187 (PMC1925093; doi:10.1186/1471-2164-8-187)
Supplement: Additional file 4 — Tables 14–21. Expression and putative function of relevant transcripts differentially expressed in one or more berry tissues according to water status. [file 1471-2164-8-187-S4.doc]

Table 14. mRNA encoding genes involved in the phenylpropanoid pathway differentially expressed in one or more tissues according to the water status. Values are expressed as log of the ratio between water-deficit stressed and well-watered intensity values in each tissue. Color code for tables 14-21: yellow: mRNAs showing significant over expression in water-deficit stressed tissue. Blue: mRNAs showing significant over expressed in well-watered tissue. Skin, SK; pulp, PL; and seed, SD.

| **Unigene** | **Affy Probe** | **First match N°** | **Description** | **Tissues** | **Cluster in WW study** | **Log ratio WD/WW pulp** | **Log ratio WD/WW skin** | **Log ratio WD/WW seed** |
| --- | --- | --- | --- | --- | --- | --- | --- | --- |
| *General phenylpropanoid pathway* | | | | | | | | |
| TC39071 | 1610206_at | BAA05643 | Phenylalanine ammonia-lyase | SK | F | 0.05 | **3.11** | 1.27 |
| TC38780 | 1610821_at | AAG10196 | Cinnamate-4-hydroxylase | SK | K | -0.04 | **1.01** | 0.37 |
| TC44994 | 1609307_at | AAC39366 | 4-coumarate:CoA ligase 1 | SK | F | 0.19 | **2.52** | 1.1 |
| AF418567 | 1616575_at | AAL09047 | Stilbene synthase 2 (Vv) | SK |  | -0.05 | **1.12** | 0.32 |
| *Lignin biosynthesis* | | | | | | | | |
| TC38507 | 1611542_at | CAA01887 | GPO1 PPO (Vv) | SK | K | 0.04 | **-1.62** | -0.01 |
| TC46387 | 1622651_at | AAU12257 | Polyphenol oxidase | SK | J | 0.33 | **2.21** | -0.62 |
| TC42415 | 1607475_s_at | AAG43822 | Caffeic acid O-methyltransferase | PL SK | J | **1.3** | **1.36** | -0.51 |
| TC45576 | 1619450_s_at | AAF44672 | Caffeic acid O-methyltransferase (Vv) | SK | L | 0.37 | **2.49** | 0.32 |
| AF239740 | 1616434_s_at | AAF44672 | Caffeic acid O-methyltransferase (Vv) | SK | L | 0.85 | **1.82** | -0.17 |
| CF207053 | 1620342_at | AAG43822 | Caffeic acid O-methyltransferase | PL SK | J | **1.43** | **1.43** | -0.65 |
| TC46166 | 1614643_at | CAA90969 | Caffeoyl-CoA O-methyltransferase (Vv) | SK | C | 0.62 | **2.11** | 0.19 |
| TC44676 | 1621728_at | CAD47830 | Hydroxycinnamoyl transferase | SK |  | 0.28 | **2.31** | 0.05 |
| TC44947 | 1614423_at | AAM34502 | Cinnamoyl CoA reductase | SK |  | -0.02 | **2.56** | -0.04 |
| TC39574 | 1619065_at | AAP55155 | Cinnamoyl-CoA reductase | PL | F | **1.04** | 0.38 | 0.14 |
| *Flavonoid biosynthesis* | | | | | | | | |
| X75969 | 1617019_at | CAA53583 | Chalcone synthase (Vv) | SD | F | 0.06 | 0.35 | **-1.01** |
| TC38410 | 1620424_at | AAL36093 | Chalcone isomerase | PL | F | **-1.15** | -0.33 | -0.99 |
| BQ798614 | 1612699_at | AAD43161 | Flavanone 3-hydroxylase | SK | C | -0.79 | **-1.23** | -0.58 |
| TC45490 | 1607607_s_at | AAT68774 | Flavanone 3-hydroxylase | PL | F | **-1.02** | -0.13 | -0.45 |
| TC46972 | 1608791_at | AAO63023 | Flavonol synthase | PL | H | **-1.04** | -0.75 | -0.81 |
| TC41648 | 1616474_at | AAQ65162 | Leucoanthocyanidin dioxygenase | PL |  | **-1.23** | 0.42 | -0.06 |
| TC46143 | 1618551_at | AAP86222 | Flavonol synthase (Vv) | SK | C | -0.75 | **1.35** | -0.31 |
| *Proanthocyanidin biosynthesis* | | | | | | | | |
| TC39641 | 1615174_s_at | AAX12185 | Leucoanthocyanidin reductase | SK | F | 0.11 | **1.69** | -0.11 |
| *Anthocyanidin biosynthesis* | | | | | | | | |
| TC38177 | 1615401_at | CAA54611 | UTP-glucose glucosyltransferase | SK |  | -0.02 | **2.49** | 0.24 |
| *Other* |  |  |  |  |  |  |  |  |
| TC48485 | 1620959_s_at | BAD18978 | Myb transcription factor VvMYBA2 (Vv) | PL | K | **2.47** | 0.01 | 0.7 |

Table 15. mRNA encoding cell wall metabolism enzymes differentially expressed in one or more tissues according to the water status. Values are expressed as log of the ratio between water-deficit and well-watered intensity values.

| **Unigene** | **Affy Probe** | **First match N°** | **Description** | **Tissues** | **Cluster in WW study** | **Log ratio WD/WW pulp** | **Log ratio WD/WW skin** | **Log ratio WD/WW seed** |
| --- | --- | --- | --- | --- | --- | --- | --- | --- |
| *Pectin metabolism* | | | | | | | | |
| TC47230 | 1611081_a_at | CAA23048 | Polygalacturonase | SK | L | -0.84 | **-1.17** | -0.95 |
| TC47738 | 1608756_at | AAP33475 | Polygalacturonase-like protein | PL | G | **-1.57** | -0.47 | 0.78 |
| TC49646 | 1621225_at | CAA23048 | Polygalacturonase | SK | L | -0.7 | **-1.04** | -0.29 |
| TC47054 | 1608799_at | NP_915049 | Pectin methylesterase | SK | C | -0.27 | **-1.41** | -0.15 |
|  |  |  |  |  |  |  |  |  |
| AF305093 | 1613339_at | AAK14075 | Polygalacturonase inhibiting protein (Vv) | PL SD | B | **1.72** | 1.49 | **-2.49** |
| AY043233 | 1620305_at | AAK81876 | Polygalacturonase PG1 (Vv) | SD | A | 0.06 | 0.8 | **2.49** |
| TC45605 | 1609278_at | BAA95794 | DC1.2 homologue | PL | F | **1.46** | 0.53 | 0.02 |
| *Xyloglucan metabolism* | | | | | | | | |
| TC38813 | 1617150_at | BAC66694 | Expansin (Vl x Vv) | PL SK | C | **-1.33** | **-1.24** | -0.83 |
| TC46110 | 1620840_at | AAM89261 | Expansin 3 | SK | C | -0.09 | **-3.1** | 0.02 |
| CF206328 | 1609909_s_at | AAO65151 | Xyloglucan endotransglycosylase | SD | B | -0.04 | -0.22 | **-1.36** |
| TC42884 | 1614426_at | AAA81350 | Xyloglucan endotransglycosylase | SD | I | -0.51 | -0.03 | **-1.17** |

Table 16. mRNA encoding transport facilitation proteins differentially expressed in one or more tissues according to the water status. Values are expressed as log of the ratio between water-deficit and well-watered intensity values.

| **Unigene** | **Affy Probe** | **First match N°** | **Description** | **Tissues** | **Cluster in WW study** | **Log ratio WD/WW pulp** | **Log ratio WD/WW skin** | **Log ratio WD/WW seed** |
| --- | --- | --- | --- | --- | --- | --- | --- | --- |
| TC45189 | 1622822_at | CAE53881 | Aquaporin | PL SK |  | **-1.7** | **-2.12** | -0.49 |
| TC38576 | 1615829_s_at | AAF78757 | Aquaporin TIP3 (Vb x Vr) | SK | K | -0.13 | **-1.77** | -0.09 |
| TC48358 | 1611614_at | BAB11467 | Na+/H+ antiporter-like protein | PL | E | **-1.43** | -1.24 | -0.27 |
| TC48913 | 1614920_at | AAK53759 | Potassium transporter HAK2p | PL |  | **-1.05** | -0.53 | 0.39 |
| TC47109 | 1615318_at | CAB40777 | Potassium transporter-like protein | SK | F | 0.76 | **2.15** | -0.64 |
| TC51669 | 1612678_at | AAO50638 | ABC transporter protein | PL | J | **1.05** | 0.45 | 0.17 |
| TC50042 | 1610363_at | AAC34225 | ABC transporter | SK | J | 0.2 | **2.39** | 1.22 |

Table 17. mRNA encoding carbohydrate accumulation-related proteins differentially expressed in one or more tissues according to the water status. Values are expressed as log of the ratio between water-deficit and well-watered intensity values.

| **Unigene** | **Affy Probe** | **First match N°** | **Description** | **Tissues** | **Cluster in WW study** | **Log ratio WD/WW pulp** | **Log ratio WD/WW skin** | **Log ratio WD/WW seed** |
| --- | --- | --- | --- | --- | --- | --- | --- | --- |
| TC40715 | 1611326_at | CAC00697 | Sugar transporter | PL | D | **-2.96** | -0.27 | -1.17 |
| TC45440 | 1611613_at | AAB47171 | Vacuolar invertase 1, (vv) | PL |  | **-1.45** | -0.41 | -0.02 |
| CF515277 | 1618478_at | BAC24804 | UDP-galactose 4-epimerase | PL SK |  | **1.04** | **1.12** | 0.28 |
| TC38597 | 1610527_at | AAM44082 | Sorbitol transporter | SK |  | -0.08 | **2.22** | 0.81 |
| TC45858 | 1612918_at | AAK43976 | Sorbitol dehydrogenase | PL SK | I | **1.32** | **1.79** | -0.43 |

Table 18. mRNA encoding proteins involved in hormone biosynthesis or signaling differentially expressed in one or more tissues according to the water status. Values are expressed as log of the ratio between water-deficit and well-watered intensity values.

| **Unigene** | **Affy Probe** | **First match N°** | **Description** | **Tissues** | **Cluster in WW study** | | **Log ratio WD/WW pulp** | | **Log ratio WD/WW skin** | | **Log ratio WD/WW seed** |
| --- | --- | --- | --- | --- | --- | --- | --- | --- | --- | --- | --- |
| *Ethylene* | | | | | | | | | | | |
| TC38334 | 1614836_s_at | CAA95856 | S-adenosyl-L-methionine synthetase 1 | SK | | L | 0.91 | **1.65** | | 0.49 | |
| TC45963 | 1619909_at | AAA81377 | S-adenosylmethionine synthetase | SK | |  | -0.19 | **1.05** | | 0.16 | |
| TC45908 | 1609995_s_at | AAA33697 | 1-aminocyclopropane-1-carboxylate oxidase | PL SK | | C | **1.68** | **1.5** | | 0.87 | |
| CF201799 | 1616698_at | BAB89351 | 1-aminocyclopropane-1-carboxylate oxidase] | PL SK | |  | **1.68** | **1.61** | | 0.83 | |
| TC47273 | 1616198_at | BAA97123 | Ethylene-responsive element binding factor | PL | | J | **1.42** | 0.9 | | 0.46 | |
| TC41585 | 1608511_at | AAR37423 | Ethylene response factor 5 (Va) | PL | | F | **1.85** | 1.29 | | 1.39 | |
| *Auxin* | | | | | | | | | | | |
| AY082522 | 1615728_at | AAL92850 | Auxin-induced protein IAA9 | SK | | E | -0.9 | **-1.03** | | -0.29 | |
| TC40543 | 1613054_at | CAA18855 | Auxin-regulated protein | SK | | K | 0.11 | **-1.94** | | 0.22 | |
| TC41864 | 1621201_at | BAB02503 | Auxin-responsive protein-related | PL SK SD | | L | **-2.52** | **-1.9** | | **-1.95** | |
| TC47591 | 1616717_at | CAC84712 | Aux/IAA protein | SK | | K | -0.37 | **-1.94** | | -1.08 | |
| TC46080 | 1620512_at | AAC99773 | Phytochrome-associated protein 2 | PL SK | | G | **-1.42** | **-1.9** | | -0.07 | |
| TC40686 | 1609591_at | AAO63268 | Auxin-induced protein 10A5 | SD | | E | 0.42 | 0.65 | | **1.16** | |
| TC41939 | 1611491_at | AAM54033 | PIN1-like auxin transport protein | PL SK | | C | **1.03** | **1.04** | | 0.39 | |
| *ABA* | | | | | | | | | | | |
| TC38630 | 1619164_at | AAL67991 | Dehydration-induced protein RD22 | SK | | F | -0.22 | **-1.17** | | -0.17 | |
| TC38295 | 1611818_at | AAL26909 | Dehydration-induced protein RD22 | SK | | K | -0.04 | **-1.85** | | 0.06 | |
| TC48377 | 1608022_at | AAR11193 | 9-cis-epoxycarotenoid dioxygenase 1 (Vv) | SK | | G | 0.87 | **1.47** | | 0.72 | |
| TC38517 | 1609893_at | AAB62947 | LTCOR11 | SK | | B | 2.03 | **2.24** | | -0.28 | |
| *Jasmonate* | | | | | | | | | | | |
| TC44915 | 1608892_at | AAP83137 | Lipoxygenase | PL | | L | **-1.35** | -1.06 | | -0.57 | |
| TC43235 | 1617751_s_at | AAP83137 | Lipoxygenase | PL | | L | **-1.37** | -1.1 | | -0.51 | |
| TC41392 | 1619407_s_at | AAC78441 | 12-oxophytodienoate reductase OPR2 | SD | | J | -0.59 | -0.46 | | **-1.19** | |
| CF405309 | 1617922_at | AAB65767 | Lipoxygenase | PL | | L | **1.12** | 1.01 | | 1.01 | |
| TC49699 | 1612190_at | AAO72741 | Allene oxide synthase | SK | | E | 1.1 | **1.46** | | 1.12 | |
| *Gibberellic acid* | | | | | | | | | | | |
| TC45582 | 1622414_at | AAT77035 | Gibberellin 20 oxidoreductase | PL SK | | I | **1.82** | **1.7** | | 0.12 | |

Table 19. mRNA encoding pathogenesis-related proteins differentially expressed in one or more tissues according to the water status. Values are expressed as log of the ratio between water-deficit and well-watered intensity values.

| **Unigene** | **Affy Probe** | **First match N°** | **Description** | **Tissues** | **Cluster in WW study** | **Log ratio WD/WW pulp** | **Log ratio WD/WW skin** | **Log ratio WD/WW seed** |
| --- | --- | --- | --- | --- | --- | --- | --- | --- |
| TC47573 | 1606625_at | AAM95447 | Class IV chitinase (Vv) | SK | L | -0.78 | **-1.24** | 0.43 |
| TC38484 | 1612444_at | CAC83581 | Major latex-like protein | SK | C | -0.03 | **-2.15** | -0.03 |
| TC38438 | 1622416_at | AAG29777 | Lipid transfer protein 3 | PL | K | **-2.13** | -0.07 | -0.44 |
| TC38608 | 1614230_at | CAH04984 | Type 1 non-specific lipid transfer protein | SK |  |  | **-1.38** | 0.06 |
| TC47234 | 1613636_at | AAS47036 | Major cherry allergen Pru av 1.0202 | SK | C | -0.46 | **-2.4** | 0.2 |
| TC38776 | 1607645_at | AAD38290 | Pathogenesis-related protein | SD | B | 0.18 | -0.15 | **-1.96** |
| TC48696 | 1611135_at | AAL36348 | Alpha-hydroxynitrile lyase | SD | B | -0.11 | 0.39 | **-1.26** |
| AF239617 | 1615595_at | AAF44667 | Beta-1,3-glucanase | PL SK | B | **3.66** | **3.14** | -0.1 |
| TC44117 | 1619916_s_at | AAR06588 | Beta-1,3-glucanase (Vr) | SK | B | 0.11 | **1.01** | -0.79 |
| TC38869 | 1613871_at | AAM95447 | Class IV chitinase (Vv) | SK |  | 0.29 | **3.07** | 0.31 |
| TC39952 | 1612050_at | CAA77656 | Acidic chitinase III | SK | B | -0.1 | **1.66** | -0.39 |
| CF202444 | 1611876_s_at | AAL01886 | Chitinase 3-like protein precursor | SK | B | -0.42 | **1.63** | -0.6 |
| TC39447 | 1606794_at | AAQ10092 | Thaumatin (Vv) | SK | F | 0.52 | **1.33** | 0.12 |
| TC38581 | 1616695_s_at | AAD55090 | Thaumatin (Vr) | PL | J | **2.28** | 1.3 | -0.63 |
| AF003007 | 1616413_at | AAB61590 | VVTL1 (Vv) | SK | B | -0.03 | **1.3** | 0.36 |
| TC45134 | 1613811_a_at | AAB61590 | VVTL1 (Vv) | SK | B | -0.29 | **1.11** | 0.14 |
| TC47283 | 1622760_at | AAG43551 | Avr9/Cf-9 rapidly elicited protein 146 | PL SK | G | **1.33** | **1.4** | 1.21 |
| TC41586 | 1620598_at | AAR36911 | Disease resistance gene | SK | G | 0.66 | **1.7** | 0.92 |
| TC41261 | 1608026_at | BAB10833 | Germin-like protein | PL SK | B | **2.31** | **3.26** | -0.2 |
| TC47633 | 1618038_at | AAM62411 | Disease resistance protein EDS0 | PL SK |  | **1.1** | **1.4** | -0.15 |

Table 20. mRNA encoding aroma biosynthesis enzymes differentially expressed in one or more tissues according to the water status. Values are expressed as log of the ratio between water deficit and well-watered intensity values.

| **Unigene** | **Affy Probe** | **First match N°** | **Description** | **Tissues** | **Cluster in WW study** | **Log ratio WD/WW pulp** | **Log ratio WD/WW skin** | **Log ratio WD/WW seed** |
| --- | --- | --- | --- | --- | --- | --- | --- | --- |
| CF206716 | 1608741_s_at | AAD56390 | 1-deoxy-D-xylulose-5-phosphate synthase | SK | C | -0.22 | **-1.08** | -0.03 |
| TC49937 | 1612338_at | AAO63403 | Alliin lyase | PL SK | E | **-1.97** | **-3.17** | -0.44 |
| TC50695 | 1608962_s_at | AAO63403 | Alliinase | SK | E | -1.64 | **-3.05** | -1.14 |
| TC40259 | 1616910_at | AAT86042 | E-beta-ocimene synthase | PL | G | **-1.15** | 0.97 | -0.09 |
| TC46045 | 1618595_at | AAU20370 | (-)-isopiperitenol dehydrogenase | PL SK |  | **-1.16** | **-1.32** | -0.76 |
| TC38916 | 1614808_at | AAG41438 | 4-methyl-5(b-hydroxyethyl)-thiazole monophosphate biosynthesis protein | PL |  | **-1.03** | -0.89 | 0.07 |
| TC39841 | 1619925_at | CAA65048 | Zeaxanthin epoxidase | SK | K | 0.06 | **-1.01** | -0.22 |
| CA818350 | 1612552_at | AAG51997 | S-adenosyl-L-methionine:salicylic acid carboxyl methyltransferase | SK | B | 0.22 | **1.85** | -0.03 |
| TC47414 | 1611452_at | CAC35167 | Arbutin synthase | SK |  | 0.1 | **1.91** | 0.64 |
| TC42069 | 1619371_at | AAM77007 | Beta-carotene hydroxylase (Vv) | PL | B | **1.62** | 1.43 | -0.01 |
| AF239740 | 1616434_s_at | AAF44672 | Caffeic acid O-methyltransferase (Vv) | SK | L | 0.85 | **1.82** | 0.17 |
| CF207053 | 1620342_at | AAG43822 | Caffeic acid O-methyltransferase | PL SK | J | **1.43** | **1.43** | -0.65 |
| TC42415 | 1607475_s_at | AAG43822 | Caffeic acid O-methyltransferase | PL SK | J | **1.3** | **1.36** | -0.51 |
| TC45576 | 1619450_s_at | AAF44672 | Caffeic acid O-methyltransferase (Vv) | SK | L | 0.37 | **2.49** | 0.32 |
| TC48824 | 1613542_at | BAC78827 | Caffeic acid O-methyltransferase | SD | B | -0.01 | 0.1 | **1.18** |
| CF206021 | 1612511_at | CAA71514 | Cytochrome P450 | SK | C | 0.16 | 1.97 | 0.59 |
| TC38220 | 1619263_at | T02955 | Cytochrome P450 | PL | J | 1.43 | 0.77 | 0.23 |

Table 21. mRNA encoding transcription factors differentially expressed in one or more tissues according to water status. Values are expressed as log of the ratio between water deficit and well-watered intensity values.

| **Unigene** | **Affy Probe** | **First match N°** | **Description** | **Tissues** | **Cluster in WW study** | **Log ratio WD/WW pulp** | **Log ratio WD/WW skin** | **Log ratio WD/WW seed** |
| --- | --- | --- | --- | --- | --- | --- | --- | --- |
| TC42939 | 1612095_at | AAK96816 | B-box zinc finger protein | SD | J | -0.29 | -0.67 | **-1.67** |
| TC41419 | 1609484_at | XP_467243 | Basic-helix-loop-helix transcription factor | PL | G | **-1.34** | -0.02 | 0.01 |
| TC40935 | 1613759_s_at | AAM97061 | CCCH-type zinc finger protein | PL | J | **-1.62** | -0.69 | -0.18 |
| TC40814 | 1618763_at | CAC00657 | Common plant regulatory factor 6 | SK | F | -0.06 | **-1.13** | -0.39 |
| TC40127 | 1606639_at | AAM97321 | Homeodomain protein GhHOX1 | PL | L | **-1.18** | -0.91 | -0.85 |
| TC49946 | 1613196_at | AAM97321 | Homeodomain protein GhHOX1 | PL | L | **-1.21** | -0.99 | -0.67 |
| AF373604 | 1621836_at | AAM21345 | MADS-box protein 5 (Vv) | SK | B | -0.48 | **-1.03** | -0.17 |
| TC42554 | 1616581_at | NP_850381 | Phytochrome-interacting factor 4 (PIF4) | PL | L | **-1.19** | -0.65 | -0.61 |
| TC42190 | 1617572_at | CAB71076 | RING finger protein | PL | E | **-1.98** | -0.29 | 0.07 |
| TC40377 | 1606575_at | AAO44037 | RING zinc finger protein | SK | K | -0.52 | **-1.05** | -0.69 |
| TC50319 | 1620013_at | BAD25417 | RING zinc finger protein | SK | E | -0.96 | **-1** | -0.29 |
| TC40359 | 1621301_at | AAG40395 | RNA-binding protein | SK | K | 0.07 | **-1.07** | -0.7 |
| TC43409 | 1606781_at | BAD68649 | TAF14b | SK | L | -0.95 | **-1.3** | 0.02 |
| BQ800205 | 1619424_at | AAK28441 | WRKY DNA-binding protein 21 | SK | K | 0.19 | **-1.07** | 0.22 |
| BQ797739 | 1618219_at | AAD33717 | YABBY3 | SK | L | -0.81 | **-1.21** | 0.21 |
| TC46692 | 1619251_at | BAD46368 | Zinc finger (B-box type) family protein | SK | E | -0.98 | **-1.19** | -0.3 |
| TC41545 | 1608141_at | AAP54875 | Zinc finger (C3HC4-type RING finger) | SK | K | 1.53 | **-1.2** | 0.24 |
| TC38863 | 1621220_at | AAF66242 | Dicyanin | PL SK | F | **1.23** | **1.35** | 0.56 |
| TC47722 | 1615083_at | CAC36939 | DOF zinc finger like protein | PL | E | **1** | 0.57 | 0.2 |
| TC43527 | 1613141_at | AAD22369 | NAM (no apical meristem)-like protein | SK | K | 0.07 | **2.52** | 0.31 |
| TC42530 | 1607620_at | AAF05865 | NAM-like protein (no apical meristem) | PL SK | B | **1.91** | **1.67** | -0.35 |
| TC45602 | 1610064_at | BAA06278 | SPF1 protein | PL | F | **1.23** | 0.68 | -0.1 |
| CA809190 | 1611285_s_at | CAE03880 | WRKY transcription factor 11 (WRKY11) | SK | J | 0.26 | **1.15** | 0.81 |
| CA809370 | 1612649_s_at | BAB16432 | WRKY transcription factor NtEIG-D48 | PL | F | **1.23** | 0.42 | 0.27 |
| CF206474 | 1610775_s_at | AAW66459 | WRKY transcription factor-b | PL SK | F | **2.1** | **1.05** | -0.19 |
| TC39121 | 1614806_s_at | AAR37421 | WRKY4 transcription factor (Va) | SK | J | 0.63 | **1.58** | 0.83 |
